# Detection of Atherosclerosis by Small RNA-Sequencing Analysis of Extracellular Vesicle Enriched Serum Samples

**DOI:** 10.3389/fcell.2021.729061

**Published:** 2021-10-12

**Authors:** Alex Hildebrandt, Benedikt Kirchner, Agnes S. Meidert, Florian Brandes, Anja Lindemann, Gero Doose, Alexander Doege, Rolf Weidenhagen, Marlene Reithmair, Gustav Schelling, Michael W. Pfaffl

**Affiliations:** ^1^Division of Animal Physiology and Immunology, School of Life Sciences Weihenstephan, Technical University of Munich, Freising, Germany; ^2^Department of Anesthesiology, University Hospital, Ludwig-Maximilians-University Munich, Munich, Germany; ^3^Institute of Human Genetics, University Hospital, Ludwig-Maximilians-University Munich, Munich, Germany; ^4^ecSeq Bioinformatics GmbH, Leipzig, Germany; ^5^Department of Vascular Surgery, Klinikum Neuperlach, Muenchen-Kliniken, Munich, Germany

**Keywords:** atherosclerosis, extracellular vesicles, small RNA-sequencing, biomarker, gene ontology

## Abstract

Atherosclerosis can occur throughout the arterial vascular system and lead to various diseases. Early diagnosis of atherosclerotic processes and of individual disease patterns would be more likely to be successful if targeted therapies were available. For this, it is important to find reliable biomarkers that are easily accessible and with little inconvenience for patients. There are many cell culture, animal model or tissue studies that found biomarkers at the microRNA (miRNA) and mRNA level describing atherosclerotic processes. However, little is known about their potential as circulating and liquid biopsy markers in patients. In this study, we examined serum-derived miRNA – profiles from 129 patients and 28 volunteers to identify potential biomarkers. The patients had four different atherosclerotic manifestations: abdominal aneurysm (*n* = 35), coronary heart disease (*n* = 34), carotid artery stenosis (*n* = 24) and peripheral arterial disease (*n* = 36). The samples were processed with an extracellular vesicle enrichment protocol, total-RNA extraction and small RNA-sequencing were performed. A differential expression analysis was performed bioinformatically to find potentially regulated miRNA biomarkers. Resulting miRNA candidates served as a starting point for an overrepresentation analysis in which relevant target mRNAs were identified. The Gene Ontology database revealed relevant biological functions in relation to atherosclerotic processes. In patients, expression of specific miRNAs changed significantly compared to healthy volunteers; 27 differentially expressed miRNAs were identified. We were able to detect a group-specific miRNA fingerprint: miR-122-5p, miR-2110 and miR-483-5p for abdominal aortic aneurysm, miR-370-3p and miR-409-3p for coronary heart disease, miR-335-3p, miR-381-3p, miR493-5p and miR654-3p for carotid artery stenosis, miR-199a-5p, miR-215-5p, miR-3168, miR-582-3p and miR-769-5p for peripheral arterial disease. The results of the study show that some of the identified miRNAs have already been associated with atherosclerosis in previous studies. Overrepresentation analysis on this data detected biological processes that are clearly relevant for atherosclerosis, its development and progression showing the potential of these miRNAs as biomarker candidates. In a next step, the relevance of these findings on the mRNA level is to be investigated and substantiated.

## Introduction

Atherosclerosis is a chronic arterial disease and a leading cause of vascular death worldwide. Although the vascular mortality risk has declined substantially over the last decades from 16% in 1980 to 4% in 2010 in high income countries, some countries (in particular Eastern Europe and parts of Asia) still report increases in mortality rates (Bennett et al., 2014; Moran et al., 2014). Despite these trends, atherosclerosis remains the leading cause of premature adult morbidity and mortality worldwide (GBD 2013 Mortality and Causes of Death Collaborators, 2015).

The pathophysiologic process leading to atherosclerosis starts with accumulation of low-density lipoproteins (LDL) in the intima (the innermost layer of arterial vessels) followed by activation of endothelial cells (ECs) and expression of adhesion molecules. Monocytes from the bloodstream attach to them and enter the intima. Here, monocytes mature into macrophages that devour lipoproteins and become foam cells (Vozenilek et al., 2018). During the inflammatory process, T lymphocytes also migrate into the intima and can trigger inflammatory processes that affect both ECs and smooth muscle cells (SMCs). It is believed that these immunological and cellular processes lead to the formation of the neointima, which causes plaque formation (Libby and Theroux, 2005). The growth of the neointima and the associated stenosis can lead to complete occlusion of the affected artery (Bentzon et al., 2014). The disease has a latency of many years and frequently coexists in more than one vascular bed. This leads to different clinical manifestations, which include ischemic heart disease, ischemic stroke, and peripheral arterial disease among others (Herrington et al., 2016). For the correct diagnosis of the various disease manifestations, it is necessary to find suitable biomarkers to apply a focused and optimized therapy. These should be easily accessible diagnostically by liquid biopsy and as specific as possible. Today, technological progress in molecular biology is leading to more and more knowledge in the context of circulating biomarkers, e.g., by analyzing extracellular vesicles (EVs) and the connected miRNAs of cardiovascular diseases. This makes EV-related miRNA biomarkers an interesting subject of investigation (Reithmair et al., 2017).

miRNAs are small single-stranded non-coding RNA molecules with a length of about 22 nucleotides. The biogenesis of miRNAs is a multistep process including endonucleolytic cleavages and hairpin formation before finally resulting in mature miRNA. These influence the synthesis of proteins by their interactions with mRNAs (Bartel, 2004). A changed expression of miRNAs can thus contribute to disease-relevant processes. Most miRNAs are located in the cell but they can also be present extracellularly in various biological fluids (circulating or extracellular miRNAs). In biofluids such as blood they can be found as cargo of EVs or bound to high-density lipoprotein cholesterol particles or Argonaut 2 proteins (Murillo et al., 2019). In this context, they are better protected from circulating RNAses and can be obtained through liquid biopsy and put into a diagnostic context.

EVs are considered to have great diagnostic potential because of their prospective role as signal transmitters in numerous physiological and pathological processes (Properzi et al., 2013; Zhang et al., 2019). It was noted that the miRNA level in EVs differs from that in the intercellular environment they were expelled from. Consequently, miRNAs are selectively packed into EVs and may regulate disease-specific mechanisms (Simeone et al., 2020).

A large number of miRNAs which control various actors and pathways involved in atherosclerosis are described (Lu et al., 2018). For instance, ECs can be influenced by suppressing the expression of the antisenescence factor SIRT1 by overexpression of, e.g., miR-34a (Deng et al., 2017). Thereby, EC senescence is associated with an increased likelihood of atherogenesis (Menghini et al., 2009). miR-217 and miR-146a are mentioned in this context as well (Sun et al., 2013; Kumar et al., 2014). Additionally, inflammatory processes can be induced by miRNAs within the endothelial layer which is enriching for the atherosclerotic environment (Libby, 2012). Smooth muscle cells can be dysregulated in their differentiation and proliferation behaviour by miRNAs like miR-22 which can cause a synthetic nature instead of a contractile one by suppressing important vascular genes and promoting disease progression (Leeper and Maegdefessel, 2018; Yang et al., 2018). Also, leukocytes such as macrophages are dysregulated by miRNAs like miR-33, leading to impaired lipid phagocytosis, cholesterol efflux, fatty acid oxidation and favouring the formation of foam cells (Karunakaran and Rayner, 2016; Ouimet et al., 2016; Ouimet et al., 2017). Some studies also point to the ability of individual miRNAs to control multiple biological processes relevant in progression of atherosclerosis. miR-21 is associated with the infiltration of macrophages into the intimate, with inflammatory reactions, proliferation of SMCs and senescence (Fan et al., 2014). Lipid uptake and inflammatory cytokine secretion are associated with miRNA-29a (Fan et al., 2014). The proliferation of SMCs and contractile gene transcription is linked to miR-221/222 (Fan et al., 2014). These and other studies suggest that a modified and disease-promoting expression level of miRNAs may be used to identify potential biomarkers for diagnosis and disease-monitoring.

The aim of this study was on the one hand to identify circulating miRNAs that can serve as biomarker candidates for atherosclerosis; on the other hand, to investigate whether a subgroup unique miRNA-profile can be determined for the four different atherosclerotic manifestations. Therefore, blood samples of 129 patients with atherosclerosis and of 28 healthy volunteers were processed with an EV enrichment protocol. The study sample included patients with abdominal aneurysm (aneu), coronary heart disease (chd), carotid stenosis (cs) and peripheral arterial disease (pad). To detect atherosclerotic processes early on and to be able to make a statement which manifestation of the disease is present could help to enable individual therapeutic approaches at an early disease stage.

## Materials and Methods

### Patient Recruitment

This study was comprised of 157 individuals, including 28 healthy volunteers (control) serving as a control group and 129 patients diagnosed with atherosclerosis (athero). The patients were recruited from the Department of Vascular Surgery of the Neuperlach Community Hospital of Munich and the University Hospital, Ludwig-Maximilians-University Munich as well as the Department of cardiac surgery of the University Hospital, Ludwig-Maximilians-University Munich.

The attending physician was responsible for the diagnosis and followed all respective guidelines. Patients were identified after the attending physician made the diagnosis of atherosclerotic disease and categorized the patients according to the presence of the following manifestations of the disorder: 34 patients had coronary heart disease (chd), 36 patients had peripheral artery disease (pad), 24 patients had carotid stenosis (cs), 35 patients had abdominal aortic aneurysm (aneu) severe enough to require surgical intervention. Patients were included into the study after evaluation for inclusion and exclusion criteria (see Table 1) and patients consent. For a comparison of the study population please see Table 2.

**TABLE 1:** Inclusion and exclusion criteria of patients participating in this study.

**Inclusion**	**Exclusion**
Cause of admission:	- No consent given
- PAD (independent of stadium)	- Under the age of 18
- Carotis stenosis (independent of stadium)	- HIV, Hepatitis B/C infection
- Thoracal or abdominal aortic aneurysm	- active inflammatory focus
- CHD (independent of stadium)	- active malign tumor disease
	- limited life expectancy of less than 6 month independent of the acute atherosclerotic disease
	- immunosuppression
	- limited ability to give consent (e.g., because of mental disability)

*Patients need to meet one of the inclusion criteria and none of the exclusion criteria to be eligible.*

**TABLE 2:** Overview of the demographic, risk factors and medication of all participants in this study.

**Demographics**	**Atherosclerosis**	**Healthy volunteer**	**P-value**
N	129	28	
Age	71.0 (65.0 – 78.0)	39.0 (34.8 – 50.5)	< 0.001
BMI	28.0 (24.8 – 31.2)	25.5 (23.9 – 26.6)	0.003
Gender (Female / Male)	33 / 96	10 / 18	0.392
**Study course**			
ICU stay (No / Yes)	53 / 76	N/A	
Death while in study (No / Yes)	128 / 1	N/A	
**Risk factors**			
Smoking (No / Yes)	80 / 49	28 / 0	< 0.001
Alcohol (No / Yes)	120 / 9	28 / 0	0.322
Hypertension (No / Yes)	30 / 99	28 / 0	< 0.001
Diabetes (No / Yes)	79 / 50	28 / 0	< 0.001
Kidney insufficiency (No / Yes)	104 / 25	28 / 0	0.024
Liver insufficiency (No / Yes)	128 / 1	28 / 0	0.399
**Medication**			
Aspirin (No / Yes)	37 / 92	28 / 0	< 0.001
Direct anticoagulation (No / Yes)	116 / 13	28 / 0	0.169
Cumarines (No / Yes)	120 / 9	28 / 0	0.322
Dual antiplatelet (No / yes)	117 / 12	28 / 0	0.198
Oral anti-diabetics No / Yes)	90 / 39	28 / 0	0.002
Insulin (No / Yes)	120 / 9	28 / 0	0.322
Betablockers (No / Yes)	63 / 66	28 / 0	< 0.001
ACE Inhibitors (No / Yes)	59 / 70	28 / 0	< 0.001
Antidepressives (No / Yes)	118 / 11	28 / 0	0.232
Statins (No / Yes)	37 / 92	28 / 0	<0.001

*All values are mean values and 25% and 75% quartiles.*

Most of the included patients had more than one atherosclerotic lesion. For all patients, their medical history was evaluated and noted. Please see Table 3 for a detailed summary of secondary diagnosis besides the cause of admission.

**TABLE 3:** Detailed overview of all participating patients of the Arteriosclerosis cohort.

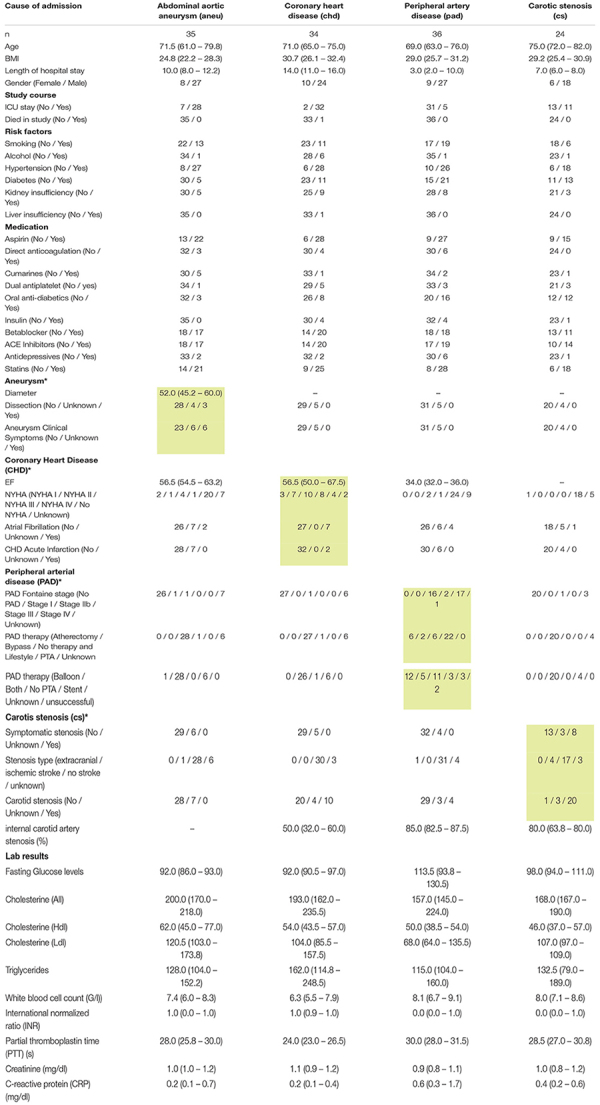

All values are mean values and 25% and 75% quartiles. The highlighted fields correspond to the cause of admission. *Patients with another cause of admission had this disease in the past patient history, but did not contribute to the current patient stay.

Comparisons of the clinical and demographics data between volunteers and patients were done with either Chi^2^-test for categorial comparisons or with Kruskal–Wallis one-way analysis of variance. A p-value < 0.05 was considered statistically significant. Statistical analysis was performed utilizing. Python Version 3.8 (Python Software Foundation, Beaverton, OR, United States). Libraries used in this study included: Numpy, Pandas and Scipy.

As very few studies on EVs and their miRNA cargo were previously performed and possible differences in EV miRNA expression levels between the different organ manifestations of atherosclerosis were not available, we had to base the sample size estimation in the statistical plan on a single but somewhat comparable study. In this study, which investigated the role of circulating extracellular vesicles (EVs), proteins, and microRNAs in ischaemic stroke, the inclusion of 81 patients with ischaemic stroke and 22 healthy controls resulted in a significant difference between expression values of a number of miRNAs between patients and healthy controls. We therefore assumed, that a further increase in the overall sample size to 129 patients and 28 volunteers would also result in statistically differences in expression values of selected miRNAs.

### Blood Sampling, Sample Preparation and Sequencing

Blood samples were drawn from patients and volunteers via venipuncture. Serum was obtained by using 9 ml serum tubes (S-Monovette, Sarstedt, Germany), allowed to clot for 30 min and subsequently centrifuged at 3400 g for 10 min at 4°C. The samples were aliquoted and stored at −80°C. The enrichment of EVs was performed by a precipitation method according to the manufacturer’s instruction (miRCURY Exosome Serum/Plasma Kit Qiagen, Venlo, the Netherlands). 1 ml of serum was used as starting volume. Cell-free total RNA was obtained with the NucleoSpin miRNA (Macherey-Nagel, Düren, Germany) in an elution volume of 30 μl. RNA yield and size distribution were determined using the RNA 6000 Pico Kit on the 2100 Bioanalyzer (Agilent Technologies, Santa Clara, United States). Total RNA was resubstituted in 8 μl of nuclease-free water after vacuum-induced centrifugal evaporation. The libraries for small RNA-sequencing were prepared with the NEBNext Multiplex Small RNA Library Prep Set for Illumina (New England Biolabs Inc, Ipswich, United States). cDNA amplification product was purified using the Monarch PCR&DNA Cleanup Kit (New England Biolabs Inc, Ipswich, United States). Yield was subsequently determined using the DNA 1000 Kit on the 2100 Bioanalyzer (Agilent Technologies, Santa Clara, United States). For each sequencing run the same amount of cDNA was pooled for electrophoretic size selection step. The extraction from the gel was carried out with the Monarch DNA Gel Extraction Kit (New England Biolabs Inc, Ipswich, United States). Bands harbouring the miRNA fraction occurred at about 147 bp and were excised. Yield and size distribution of pooled samples were assessed using the High Sensitivity DNA Kit on the 2100 Bioanalyzer (Agilent Technologies, Santa Clara, United States). Sequencing of all samples was performed in four single-end sequencing runs in 50 cycles on the HiSeq2500 (Illumina Inc. San Diego, United States). A summary about the sample composition of each sequencing run is given in Table 4.

**TABLE 4:** Summary of sequenced and analysed samples per sequencing run.

**Group**	**First sequencing run before | after filtering**	**Second sequencing run before | after filtering**	**Third sequencing run before | after filtering**	**Fourth sequencing run before | after filtering**
Abdominal Aneurysm	13 | 13	8 | 7	5 | 4	9 | 6
Coronary Heart Disease	13 | 13	7 | 7	7 | 6	7 | 6
Carotid Stenosis	10 | 10	9 | 9	2 | 2	3 | 3
Peripheral Artery Disease	11 | 11	7 | 5	7 | 7	11 | 6
Control	0 | 0	16 | 16	4 | 4	8 | 5

*Number on the left shows sequenced samples and number on the right analysed ones. Non-analysed samples did not meet the thresholds for a sufficient sequencing quality (A minimum of 500,000 reads altogether and 7% of mapped miRNA in relation to total library size).*

### Bioinformatic Analysis of Small RNA-Sequencing Data

#### Data Processing

FastQC (version 0.11.9) was used to quality check each sequencing dataset. Adaptor sequences of reads were trimmed with btrim32 (version 0.3.0). Reads without any adaptor were removed as well as reads with less than 16 nucleotides in length. The mapping of reads was performed with bowtie (version 1.2.3). The cut off for reads was set to maximum one mismatch. Additional parameters that limit alignment to the sense strand (–norc) and output to the single best match in terms of mismatch quality (–best) were applied. References of non-coding RNA sequences for ribosomal RNA (rRNA), small nuclear RNA (snRNA), small nucleolar RNA (snoRNA) and transfer RNA (tRNA) were downloaded from RNACentral (release 12). miRNA references were obtained from miRBase (release 22.1). Mapping was performed sequential. First sequences of rRNA and tRNA were mapped and eliminated from the dataset. Subsequently miRNAs as well as snoRNAs and snRNAs were identified and counted. By mapping directly on mature sequences of the small RNA transcriptome, read counts were generated by calling the sum of reads matching each mature sequence. The miRNA analysis pipeline was frequently and successfully applied in various biomarker studies (Spornraft et al., 2014; Buschmann et al., 2016, 2018; Reithmair et al., 2017).

#### Differential Gene Expression Analysis

A differential gene expression (DGE) analysis was performed using R (version 4.0.3; R Core Team, 2020) and the DESeq2 (version 1.28.1; Love et al., 2014) package. Since we used four sequencing runs to collect all samples and each run had different sample distribution per group, in addition to different library sizes of the individual data sets, a normalization method and batch correction was used. All these possible biases were first balanced out by the normalization of raw reads by calculating a sample specific scaling factor using the mean of ratios methods while any batch effects were accounted for through linear modelling. The algorithms are implemented in the DESeq2 package. The Benjamin-Hochberg method was applied to correct for false discovery. Two result sets were obtained; a default set by filtering for p-value ≤ 0.1 and a more stringent one by filtering additionally for an absolute log_2_ fold change (| log2FC|) ≥ 1 and base mean ≥ 50.

#### Overlap Analysis

Both the result tables with the default cut-off and the stricter filter criteria of the DGE analysis were used for the overlap analysis. The analysis was carried out using R (version 4.0.3; R Core Team, 2020) and the veccompare (version 0.1.0; Levernier and Wacha, 2017) package.

#### Unsupervised and Supervised Clustering

Unsupervised clustering was performed using principal component analysis (PCA) using R (version 4.0.3; R Core Team, 2020), stats (version 4.0.3, R Core Team, 2020) and ggplot2 (version 3.3.2, Wilkinson, 2011) packages for calculation and plotting. The dataset was also filtered to the 500 most variant miRNAs of the whole dataset. Supervised clustering was done by sparse partial-least-squares discriminant analysis (sPLS-DA) using R (version 4.0.3; R Core Team, 2020) and mixOmics (version 6.12.2, Rohart et al., 2017) package. PCA as well as sPLS-DA were carried out in two ways. First, the atherosclerotic group (*n* = 129) was compared with the control group (*n* = 28). Second, all four atherosclerotic subgroups and the control were given as input. To find optimal number of components for the sPLS-DA the distance was measured by three algorithms and ranked with the balanced error rate (BER) and the total error rate. Subsequently, optimal numbers of features (miRNAs) for each component were determined.

#### Overrepresentation Analysis

Overrepresentation analysis was carried out with miRNAs resulting from the DGE analysis with the stricter filter criteria using R and clusterProfiler (version 3.16.1, Wu et al., 2021) package. The mRNA targets of miRNAs were determined using the miRTarBase database (release 8.0). The annotation of targets was supported by strong experimental evidences (reporter assay or western blot). Pathways and processes that targeted genes contributed to were identified using the Gene Ontology (GO) database for biological processes. To reduce redundant GO terms in the result, the *simplify* function implemented in the package was applied and filtering steps with the GO.db (version 3.11.4, Carlson, 2019) package for R 4.0.3 (R Core Team, 2020) were carried out.

### Ethics Approval and Patient Consent for Study Participation

The study was approved by the Ethics Committee of the Medical Faculty of the University of Munich (protocol #17-572). The study was carried out according to the World Medical Association Declaration of Helsinki and all study samples were pseudonymized during analysis. Written informed consent for publication of blinded individual personal data was obtained from each participant.

## Results

### Sequencing Quality and Mapping Distribution

**FIGURE 1:**
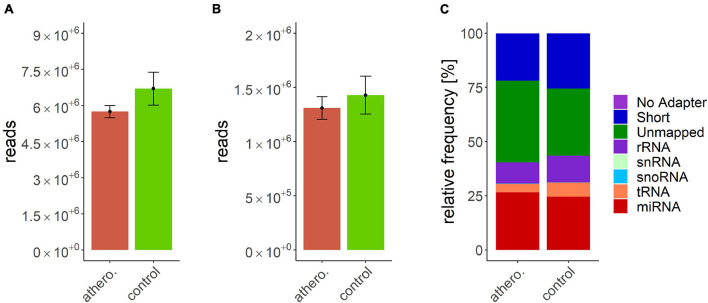
**(A)** mean library size: athero: 5.74 ± 0.25 M.; control: 6.7 ± 0.69 M.; **(B)** mean of mapped miRNAs: athero: 1.31 ± 0.11 M.; controlr: 1.43 ± 0.17 M.; **(C)** mean relative frequency of mapped read counts. athero = atherosclerotic group; control = control group; No Adapter = reads without adapter; Short = reads smaller than 16 nt; Unmapped = reads which are not mapped to reference; rRNA = reads mapped as rRNA; snRNA = reads mapped as snRNA; snoRNA = reads mapped as snoRNA; tRNA = reads mapped as tRNA; miRNA = reads mapped as miRNA.

**FIGURE 2:**
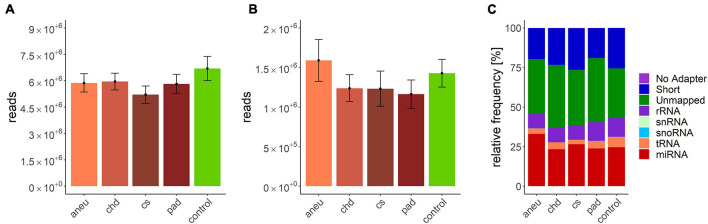
**(A)** mean library size: aneu: 5.88 ± 0.53 M.; chd: 5.96 ± 0.47 M.; cs: 5.21 ± 0.49 M.; pad: 5.83 ± 0.54 M.; control: 6.7 ± 0.69; **(B)** mean of mapped miRNAs: aneu: 1.6 ± 0.26 M.; chd: 1.24 ± 0.17 M.; cs: 1.23 ± 0.22 M.; pad: 1.16 ± 0.18 M.; control: 1.43 ± 0.17; **(C)** mean relative frequency of mapped read counts. aneu = abdominal aneurysm; chd = coronary heart disease; cs = carotid stenosis; pad = peripheral artery disease; control = control group; No Adapter = reads without adapter; Short = reads smaller than 16 nt; Unmapped = reads which are not mapped to reference; rRNA = reads mapped as rRNA; snRNA = reads mapped as snRNA; snoRNA = reads mapped as snoRNA; tRNA = reads mapped as tRNA; miRNA = reads mapped as miRNA.

Data processing resulted in a count table with the dimensions of 2165 miRNAs and 157 samples. For each miRNA, at least one read was counted in one sample. Next, samples with an insufficient sequencing result were determined and taken out from further analysis. A minimum of 500000 reads altogether and 7% of mapped miRNAs in relation to total library size were set as thresholds. This reduced the dimensions for further analysis to 2165 miRNAs and 140 samples. A summary of included samples of each sequencing run is given in Table 4. For all data sets the per-base sequence quality had a Phred score over 32. Highest mean library size was observed in the control group with 6.7 M reads. Fewer reads were assigned to the other groups (athero 5.7 M, aneu 5.9 M, chd 6 M, cs 5.2 M, pad 5.8 M) (Figures 1A, 2A). Mean of reads mapped to miRNA reference for the athero and control group was nearly the same (athero 1.3 M, control 1.4 M) (Figure 1B). The aneu group showed with 1.6 M the highest number of mapped reads on average. In the other subgroups, 0.2 – 0.4 M reads less were mapped on average (Figure 2B). The relative mapping distributions of mapped read counts to different RNA species for the athero group and for the control group (Figure 1C) as well as for the individual subgroups (Figure 2C) were comparable. Beside different RNA species the reads were also classified as reads shorter than 16nt (Short), as reads without an adaptor (No Adapter) and as unmapped reads (Unmapped) (Figures 1C, 2C). The aneu group showed the highest relative frequency of mapped miRNAs with 33.1%, followed by the cs and athero group with 26.6%, the control group with 24.6%, the pad group with 23.9% and the chd group with 23.4% (Supplementary Table 1). A detailed assignment of the individual components of the relative mapping distribution and its percentage of the standard error are given in the Supplementary Tables 1,2.

### Bioinformatic Analysis of Small RNA-Sequencing Data

#### Differential Gene Expression Analysis With DESeq2

**FIGURE 3:**
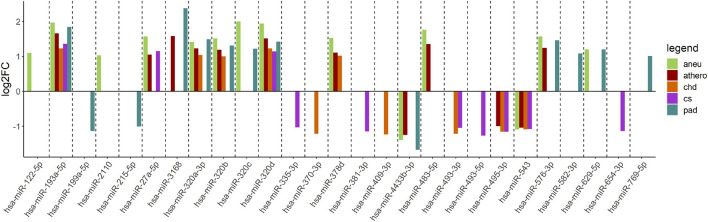
Log2FC of summarized differentially expressed miRNAs from the comparison of each group vs. control. The log2FC is given on the y-axis. aneu = abdominal aneurysm, athero = atherosclerosis, chd = coronary heart disease, cs = carotid stenosis, pad = peripheral artery disease, log2FC = log2 fold change.

When comparing the athero group with the control group, the DGE with the default cut-off (adjusted p-value ≤ 0.1) resulted in 114 differentially expressed miRNAs (Supplementary Table 3). Filtering the results (| log2FC| ≥ 1, adjusted p-value ≤ 0.1 and base mean ≥ 50) yielded 12 differentially expressed miRNAs (Supplementary Table 4). The mean log2FC of all differentially expressed and filtered miRNAs for all group comparisons against the control was 1.38 ± 0.31. The highest | log2FC| was 2. When comparing the individual subgroups with the control group, the following numbers of differentially expressed miRNAs within filtering criteria were found: aneu vs. control (*n* = 14), chd vs. control (*n* = 10), cs vs. control (*n* = 10), pad vs. control (*n* = 13) (Supplementary Tables 5–8). The filtered results of the DGE analysis are summarized in Figure 3. A total of 27 miRNAs which were expressed differentially in atherosclerotic groups compared to the control group were found. miR-193-5p and miR-320d were differentially expressed in all groups compared with the control.

#### Overlap Analysis of the Differentially Expressed miRNAs of the Individual Subgroups

**FIGURE 4:**
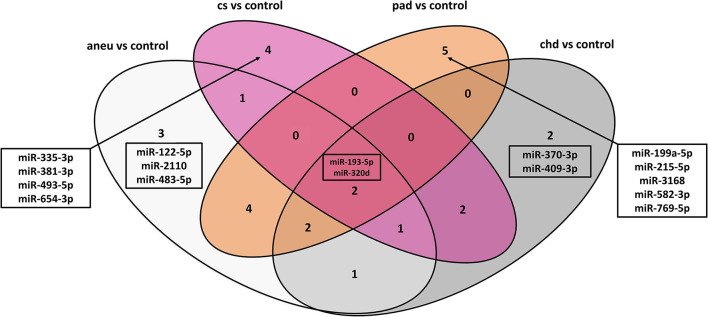
Venn-diagram representing the result of the overlap analysis; aneu = abdominal aneurysm; chd = coronary heart disease; cs = carotid stenosis; pad = peripheral artery disease.

The following overlap analysis was carried out with the resulting miRNAs of the DGE analysis applying stricter filter criteria (| log2FC| ≥ 1, adjusted p-value ≤ 0.1 and base mean ≥ 50). Overlapping and group-specific as well as uniquely differentially expressed miRNAs are summarized in Table 5 and plotted as a Venn-diagram in Figure 4. Group-specific differentially expressed miRNAs were the ones that resulted under stricter filter criteria but were also found in other groups and had a adjusted *p*-value below 0.1. Uniquely differentially expressed miRNAs were the ones that were found exclusively in one group. miR-122-5p, miR-2110 and miR-483-5p were found to be group-specific to patients with aneu whereas miR-122-5p and miR-483-5p were exclusive. miR-335-3p, miR-381-3p, miR-493-5p and miR-654-3p were found in patients with cs. miR-654-3p was exclusive for this group. In patients with chd no unique miRNA was found but miR-370-3p and miR-409-3p were group-specific. miR-199a-5p, miR-215-5p, miR-3168, miR-582-3p and miR-769-5p were found in patients with pad whereas miR-3168, miR582-3p and miR-769-5p were found to be uniquely differentially expressed for this group. Common miRNAs of all groups which met the stricter filter criteria were miR-193-5p and miR-320d.

**TABLE 5:** Differential expression of unique miRNAs for each atherosclerotic subgroup.

	**Unique for coronary heart disease**			**Unique for carotid stenosis**			**Unique for abdominal aneurysm**			**Unique for peripheral arteria disease**		
**Gene**	**group specific**	**log2FC**	**padj.**	**group specific**	**log2FC**	**padj.**	**group specific**	**log2FC**	**padj.**	**group specific**	**log2FC**	**padj.**
miR-370-3p	**yes**	–1.220	0.007	**no**	–0.972	0.076	**no**	–	–	**no**	–	–
miR-409-3p	**yes**	–1.241	0.006	**no**	–0.996	0.056	**no**	–	–	**no**	–	–
miR-335-3p	**no**	–	–	**yes**	–1.031	0.036	**no**	–0.828	0.042	**no**	–	–
miR-381-3p	**no**	–0.921	0.037	**yes**	–1.149	0.036	**no**	–0.782	0.081	**no**	–	–
miR-493-5p	**no**	–	–	**yes**	–1.271	0.045	**no**	–0.921	0.096	**no**	–	–
**miR-654-3p**	**no**	–	–	**yes**	–1.137	0.039	**no**	–	–	**no**	–	–
**miR-122-5p**	**no**	–	–	**no**	–	–	**yes**	1.098	0.050	**no**	–	–
miR-2110	**no**	–	–	**no**	–	–	**yes**	1.028	< 0.001	**no**	0.837	0.014
**miR-483-5p**	**no**	–	–	**no**	–	–	**yes**	1.765	0.027	**no**	–	–
miR-199a-5p	**no**	–0.820	0.036	**no**	–0.833	0.064	**no**	–0.730	0.061	**yes**	1.136	0.003
miR-215-5p	**no**	–0.988	0.007	**no**	–	–	**no**	–0.677	0.074	**yes**	1.006	0.005
**miR-3168**	**no**	–	–	**no**	–	–	**no**	–	–	**yes**	–2.380	0.004
**miR-582-3p**	**no**	–	–	**no**	–	–	**no**	–	–	**yes**	–1.081	0.013
**miR-769-5p**	**no**	–	–	**no**	–	–	**no**	–	–	**yes**	–1.012	0.052

*Input for the overlap analysis was the resulted differentially expressed miRNAs of the DGE analysis with stricter filter criteria (adjusted p-value ≤ 0.1, log_2_ fold change (| log2FC|) ≥ 1, base mean ≥ 50). Group specific miRNAs meet the stricter filter criteria. Genes in bold were only found in the corresponding group as significant differentially expressed (padj. < 0.1).*

When using differentially expressed miRNAs that resulted with default cut-offs (adjusted p-value ≤ 0.1), 29 group-specific miRNAs were found for aneu, 16 for chd, 15 for cs and 34 for pad (Supplementary Table 9). 11 miRNAs (miR-125a-3p, miR-1306-5p, miR-193a-5p, miR-199a-5p, miR-22-3p, miR-22-5p, miR-320d, miR-378i, miR-543, miR-548ad-5p, miR-576-3p) were commonly differentially expressed in all subgroups. Some miRNAs could be assigned in both overlap analyses (default cut-offs and stricter filter criteria) to one group. miR-654-3p was found in both analyses for the cs group. miR-122-5p and miR-483-5p were found for the aneu group, miR-3168, miR-583-3p, and miR-769-5p for the pad group and for the chd group the result did not overlap.

#### Unsupervised and Supervised Clustering

**FIGURE 5:**
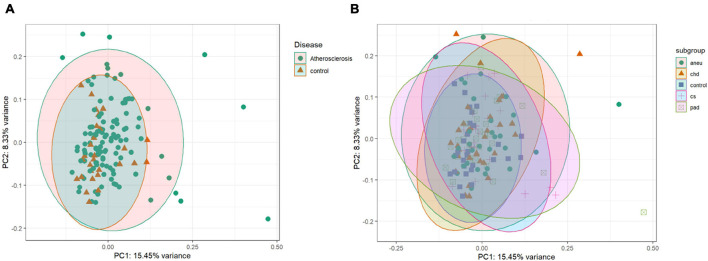
Unsupervised clustering results: **(A)** is the principal component analysis (PCA) result of the comparison of the athero and control group. **(B)** is showing the results of the subgroups and control comparison. Dataset was reduced to the 500 most variant miRNAs; PC1 = principal component 1; PC2 = principal component 2; aneu = abdominal aneurysm; chd = coronary heart disease; cs = carotid stenosis; pad = peripheral artery disease.

The unsupervised clustering was done by PCA. The data points of the different groups overlap in both comparisons. Even by reducing the dataset to the 500 most variant miRNAs as input for the analysis the expression variance between groups is overall too small. The results of the PCA analysis are shown as a plot in Figure 5. Figure 5A (PC1 and PC2) represents the comparison of atherosclerotic against control while Figure 5B (PC1 and PC2) displays the comparison of all subgroups and the control.

**FIGURE 6:**
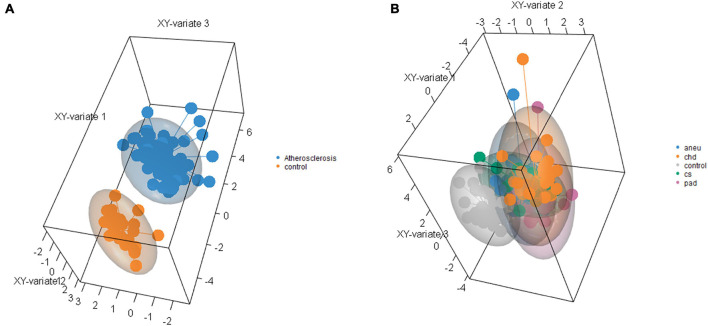
Results of sparse partial-least-squares discriminant analysis for two components. **(A)** Comparing athero group and control group and **(B)** comparing subgroups and control; aneu = abdominal aneurysm; chd = coronary heart disease; cs = carotid stenosis; pad = peripheral artery disease.

Supervised clustering of the multivariate dataset was carried out by a sPLS-DA. The analysis resulted in a differentiation between the athero and control group by nine miRNAs as discriminator (Figure 6A). One of the miRNAs (miR-193a-5p) was also determined by the DGE analysis with DESeq2. To differentiate between the individual subgroups and the control, 34 miRNAs (Supplementary Table 11) were determined by the sPLS-DA (Figure 6B). Three (miR-193a-5p, 199a-5p, 215-5p) out of them were also obtained in the DGE analysis. Both results, one with the weighted coefficient of each miRNA and the other one with the principal component (PC) it is assigned to are summarized in Supplementary Tables 10,11.

#### Overrepresentation Analysis

Differentially expressed miRNAs which were detected in the DGE analysis were the starting point to determine mRNA targets (Table 6). Only annotations supported by strong experimental evidence from the miRTarBase database were used.

**TABLE 6:** Differentially expressed miRNAs and their targeted mRNAs.

**miRNA**	**Log2FC**	**Adjusted p-value (FDR)**	**Targeted mRNAs**
**Atherosclerosis vs. Control**			
miR-193a-5p	1.66	<0.001	ERBB2, IGF2BP1, ING5, MTOR, NLN, PIK3R3, SRR, TFAP2A, TP73, WT1
miR-27a-5p	1.05	0.027	EGFR, GREM1, MXI1, PEBP1, PPARA, SFRP1 SNAP25, TXN2
miR-320b	1.19	0.002	DLX5, MYC, NOD2
miR-320d	1.51	<0.001	GNAI1, RBFOX2
miR-483-5p	1.35	0.045	CKB, ALCAM, FAM160B2, MAPK3, RHOA, SRF, NOTCH3
miR-495-3p	–1	0.009	AKT1, ATP7A, BMI1, RUNX3, FOXC1, HSPA5, MAT1A, MEIS1, PBX3, ABCB1, CCL2, SOX9, HMGA2, SMR3B, PTP4A3, TBC1D9, MTA3
miR-543	–1.04	0.003	KRAS, MMP7, NOS3, PTK2, TWIST1, HMGA2, MTA1, SIRT1, FBXO11
miR-576-3p	1.24	<0.001	CCND1
**Abdominal aneurysm vs. Control**			
**miR-122-5p**	1.10	0.050	ADAM10, ALDOA, ANK2, ANXA11, RHOA, ATP1A2, AXL, BAX, BCL2L1, BCL2L2, CCNG1, CDK4, CREB1, CYP7A1, DUSP2, EGFR, FUT8, GYS1, HMOX1, IGF1R, IL1A, MECP2, MEF2D, NCAM1, P4HA1, PDK4, PKM, PRKAB1, MAPK11, PTPN1, RAC1, SLC7A1, SRF, ADAM17, TPD52L2, VEGFC, WNT1, PRKRA, SOCS1, TBX19, NUMBL, ENTPD4, AKT3, TRIB1, SPRY2, AP3M2, FOXJ3, PEG10, LPIN1, XPO6, CTDNEP1, SLC7A11, DSTYK, CLIC4, FOXP1, NT5C3A, RAB6B, GALNT10, UBAP2, ZNF395, NOD2, AACS, FUNDC2, RAB11FIP1, NFATC2IP, G6PC3, EGLN3, ACVR1C, FAM117B
miR-193a-5p	1.97	<0.001	ERBB2, IGF2BP1, ING5, MTOR, NLN, PIK3R3, SRR, TFAP2A, TP73, WT1
miR-27a-5p	1.57	0.002	EGFR, GREM1, MXI1, PEBP1, PPARA, SFRP1 SNAP25, TXN2
miR-320b	1.51	0.002	DLX5, MYC, NOD2
miR-320c	2.00	<0.001	PRDM1, EZH2, GNAI1, IRF4, SMARCC1, XBP1, NOD2
miR-320d	1.94	<0.001	GNAI1, RBFOX2
**miR-483-5p**	1.76	0.027	CKB, ALCAM, FAM160B2, MAPK3, RHOA, SRF, NOTCH3
miR-543	–1.09	0.026	KRAS, MMP7, NOS3, PTK2, TWIST1, HMGA2, MTA1, SIRT1, FBXO11
miR-576-3p	1.57	<0.001	CCND1
miR-629-5p	1.2	<0.001	HNF4A, TRIM33
**Coronary heart disease vs. Control**			
miR-193a-5p	1.23	0.003	ERBB2, IGF2BP1, ING5, MTOR, NLN, PIK3R3, SRR, TFAP2A, TP73, WT1
miR-320d	1.23	0.019	GNAI1, RBFOX2
**miR-370-3p**	–1.22	0.007	MAP3K8, CPT1A, CTNNB1, GADD45A, FOXM1, FOXO1, FOXO1, MGMT
**miR-409-3p**	–1.24	0.006	AKT1, ANG, CTNND1, ELF2, FGA, FGB, FGG, GAB1, IFNG, MET, MGMT
miR-493-3p	–1.22	0.017	RHOC, MXI1, MAP2K7, FZD4, DKK1
miR-495-3p	–1.16	0.018	AKT1, ATP7A, BMI1, RUNX3, FOXC1, HSPA5, MAT1A, MEIS1, PBX3, ABCB1, CCL2, SOX9, HMGA2, SMR3B, PTP4A3, TBC1D9, MTA3
miR-543	–1.09	0.017	KRAS, MMP7, NOS3, PTK2, TWIST1, HMGA2, MTA1, SIRT1, FBXO11
**Carotid stenosis vs. Control**			
miR-193a-5p	1.36	0.005	ERBB2, IGF2BP1, ING5, MTOR, NLN, PIK3R3, SRR, TFAP2A, TP73, WT1
miR-27a-5p	1.15	0.087	EGFR, GREM1, MXI1, PEBP1, PPARA, SFRP1 SNAP25, TXN2
miR-320d	1.14	0.067	GNAI1, RBFOX2
**miR-335-3p**	–1.03	0.036	ESR1, NOS3, PAX6
**miR-381-3p**	–1.15	0.036	CD1C, GJA1, ID1, NFKBIA, P2RX5, TWIST1, WEE1, HDAC4, TBC1D9, ANO1
miR-493-3p	–1.05	0.084	RHOC, MXI1, MAP2K7, FZD4, DKK1
miR-495-3p	–1.16	0.045	AKT1, ATP7A, BMI1, RUNX3, FOXC1, HSPA5, MAT1A, MEIS1, PBX3, ABCB1, CCL2, SOX9, HMGA2, SMR3B, PTP4A3, TBC1D9, MTA3
miR-543	–1.08	0.045	KRAS, MMP7, NOS3, PTK2, TWIST1, HMGA2, MTA1, SIRT1, FBXO11
**miR-654-3p**	–1.14	0.039	CDKN1A
**Peripheral artery disease vs. Control**			
miR-193a-5p	1.84	<0.001	ERBB2, IGF2BP1, ING5, MTOR, NLN, PIK3R3, SRR, TFAP2A, TP73, WT1
**miR-199a-5p**	–1.14	0.002	ACVR1B, JAG1, APOE, DDR1, CAV1, CD44, CDH1, CDH2, CDKN1C, CTSC, CLTC, CCR7, EDN1, ERBB2, ERBB3, ERN1, ETS1, EZH2, GSK3B, HIF1A, HK2, HSPA5, IKBKB, ITGA3, JUNB, KRAS, LDLR, LIF, SMAD3, SMAD4, MAP3K11, NFKB1, PDE4D, PIK3CD, PSMD9, PTGS2, NECTIN1, SMARCA2, SNAI1, SULT1E1, TGFB2, TGFBR1, UNG,, WNT2, LIN7A, VEGFA, WNT2, FZD4, FZD6, MAP4K3, BECN1, LIN7A, KL, MAFB, PIAS3, ATF6, RAB21, SIRT1, SIRT1, GPR78, RND1, SETD2, DRAM1, DNAJA4, WNK1, ARHGAP12, TMEM54, OSCP1, SLC27A1
**miR-215-5p**	–1.01	0.005	ACVR2B, ALCAM, XIAP, RUNX1, NID1, RB1, ZEB2, PTPRT, SIGLEC8, CTNNBIP1, WNK1, KDM1B
miR-320b	1.31	0.004	DLX5, MYC, NOD2
miR-320c	1.22	0.045	PRDM1, EZH2, GNAI1, IRF4, SMARCC1, XBP1, NOD2
miR-320d	1.42	0.007	GNAI1, RBFOX2
miR-576-3p	1.46	<0.001	CCND1
**miR-582-3p**	1.08	0.011	SFRP1, AXIN2, DKK3
miR-629-5p	1.2	<0.001	HNF4A, TRIM33
**miR-769-5p**	1.01	0.052	GSK3B, TRAPPC2B

*Targets were determined using the miRTarBase database with annotations supported by strong experimental evidence. Log2FC and FDR were calculated by DGE analysis with DESeq2. Bold-marked miRNAs are exclusively differentially expressed. Log2FC = log_2_ fold change; B-H p-value = adjusted p-value (Benjamini & Hochberg); FDR = false discovery rate.*

**FIGURE 7:**
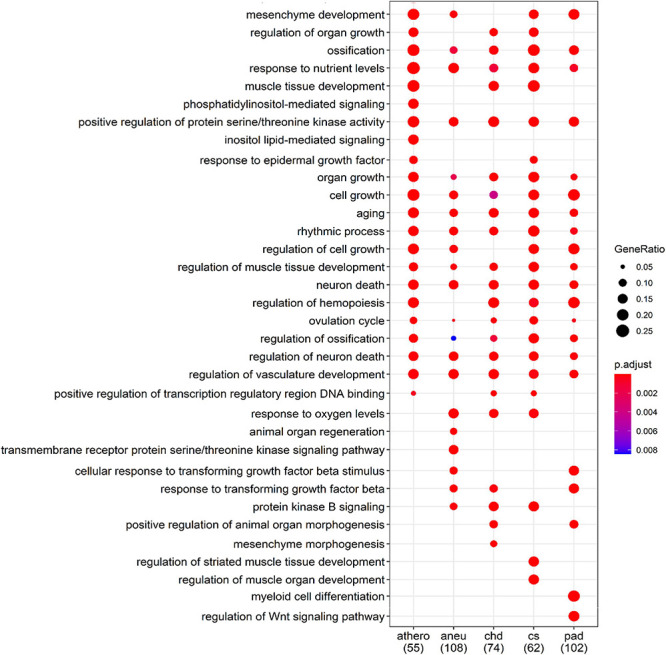
Comparison of the first 34 most enriched GO terms across groups. Gene Ratio indicates the percentage of GO term annotated genes in the gene set. The numbers in brackets indicate the size of the gene set per group; athero = atherosclerosis; aneu = abdominal aneurysm; chd = coronary heart disease; cs = carotid stenosis; pad = peripheral artery disease.

The biological processes controlled by these genes were determined using the GO database. The filtered results of the ORA for each group are summarized in Supplementary Tables 12–16. Part of the ORA result with the best ranked enriched GO terms of all individual groups is illustrated in a dot plot (Figure 7). Here 13 regulated biological processes were assigned to all groups. Group specific processes were found, too: 2 for the athero group, 2 for the aneu group, 1 for the chd group and 2 for the cs and pad group. Most of the GO terms presented in the dot plot could be linked to disease related processes of atherosclerosis.

For a better overview of the dot plot, further GO terms from the ORA analysis have been summarised in Table 7. Here, the individual GO terms were assigned to seven different biological processes that are associated with the development and progression of atherosclerosis. These are processes related to the growth and development of endothelial cells which play an important role in the development of atherosclerosis, processes indicating immunological involvement and the presence of messenger substances, processes contributing to the remodeling and mineralization and hardening of tissue, processes related to the blood vessel system in general, processes related to the presence of oxidative stress, processes referred to the metabolism of lipids and glucose and generally to aging.

**TABLE 7:** Biological processes that can be associated with atherosclerosis and the associated gene ontology (GO) terms of the overrepresentation analysis (ORA).

**Biological processes related to atherosclerosis**	**GO Term found in ORA**
Processes related to the growth and development of endothelial cells which play an important role in the development of atherosclerosis.	• cellular response to vascular endothelial growth factor stimulus
	• regulation of endothelial cell migration
	• vascular endothelial growth factor signaling pathway
	• endothelial cell differentiation
	• endothelial cell differentiation
	• endothelial cell activation
Processes indicating immunological involvement and the presence of messenger substances.	• regulation of leukocyte apoptotic process
	• leukocyte cell-cell adhesion
	• positive regulation of leukocyte chemotaxis
	• regulation of leukocyte migration
	• regulation of leukocyte differentiation
	• regulation of leukocyte apoptotic process
	• T cell differentiation
	• positive regulation of T cell activation
	• T cell differentiation
	• alpha-beta T cell activation involved in immune response
	• negative regulation of lymphocyte migration
	• lymphocyte differentiation
	• cytokine secretion
	• positive regulation of cytokine production
	• cytokine secretion involved in immune response
	• cellular response to interleukin-1
	• cellular response to interleukin-6
	• cellular response to transforming growth factor beta stimulus
	• cellular response to tumor necrosis factor
	• negative regulation of cellular response to growth factor stimulus
Processes that contribute to the remodeling and mineralization and hardening of tissue.	• regulation of ossification
	• osteoblast differentiation
	• regulation of osteoblast differentiation
	• regulation of osteoblast proliferation
	• negative regulation of osteoblast differentiation
	• regulation of biomineralization for plaque calcification
	• tissue remodeling
	• connective tissue development
	• regulation of tissue remodeling
	• regulation of muscle tissue development
	• tissue homeostasis
Processes related to the blood vessel system in general.	• regulation of vasculature development
	• cellular response to vascular endothelial growth factor stimulus
	• regulation of vasculature development
	• vasculogenesis
	• coronary vasculature morphogenesis
	• regulation of vascular permeability
	• artery morphogenesis, artery development
Processes related to the presence of oxidative stress.	• cellular response to oxidative stress
	• cellular response to hypoxia
	• negative regulation of oxidative stress-induced cell death
Processes referred the metabolism of lipids and glucose.	• positive regulation of cholesterol efflux
	• cholesterol homeostasis
	• cholesterol transport regulation of fatty acid beta-oxidation
	• fatty acid homeostasis
	• response to fatty acid
	• regulation of fatty acid metabolic process
	• positive regulation of lipid metabolic process
	• regulation of lipid storage
	• regulation of phospholipid metabolic process
	• regulation of lipid kinase activity
	• regulation of triglyceride metabolic process
	• triglyceride homeostasis
	• cellular response to glucose stimulus
	• regulation of glucose metabolic process
	• cellular glucose homeostasis
	• regulation of fat cell differentiation
Processes generally concerned to aging.	• aging • cellular senescence
	• negative regulation of cell aging
	• regulation of cell aging
	• regulation of cellular senescence
	• cellular senescence

## Discussion

In this study, the transcriptional serum-derived miRNA fingerprint from EVs obtained from four atherosclerotic subgroups and a control group was analysed. These EV-associated miRNAs were investigated to determine a miRNA set serving as potential circulating biomarkers for the identification of atherosclerotic processes and distinguishing between different manifestations. On the one hand, the transcriptional profile of the atherosclerotic group was compared (*n* = 129) with the control group (*n* = 28) and on the other hand, each of the four subgroups was compared between each other and with the control group.

Differentially expressed miRNAs were found in the DGE analysis (filter criteria: | log2FC| ≥ 1, adjusted p-value ≤ 0.1 and base mean ≥ 50) between the individual subgroups and the control, indicating that a differentiation of the varying manifestations based on the miRNA profiles is possible. Furthermore, the overlap analysis revealed the presence of group-specific and uniquely differentially expressed miRNAs that can be used to characterize individual manifestations. These could be used as circulating candidate signatures to diagnostically assign individual patients to certain subgroups of atherosclerosis and thus to be able to pursue more targeted therapeutic approaches. Some of the differentially expressed miRNAs could also be determined in the supervised clustering, supporting the results of the DGE analysis. We did a cross validation (M-fold validation) within the sPLS-DA analysis. This serves to increase the statistical significance of the results. Other validation strategies are also possible. One option would be to collect independent samples with same manifestations and use RT-qPCR analysis with potential biomarkers from this study to check if they are present. Another way is to divide the samples from this study into a training set to identify biomarker candidates and a test set to validate the results using RT-qPCR analyses. However, this would reduce the statistical power of the DGE by reducing the number of samples for each group.

The relevance of this finding is confirmed by published studies describing miRNAs found in our study as biomarkers for atherosclerosis. miR-320b, which was differentially expressed in all group comparisons except for cs in our study, was associated as a potential biomarker for ischemic stroke (Zhang et al., 2016). miR-27a-5p appeared in our results as discriminator when comparing the aneu and cs group with the control. This miRNA was linked to atherosclerotic processes in various ways (Chen et al., 2012). miR-483-5p was found to be upregulated in patients with acute myocardial infarction (Li et al., 2019) and we assigned it uniquely differentially expressed in the aneu group. In a cell culture study with vascular smooth muscle cells it was shown that a reduced level of miR-381-3p could be associated with the atherosclerotic environment, e.g., in inflammatory reaction, oxidative stress, proliferation and migration of immune cells (Zhu et al., 2021). We found this miRNA differentially expressed in the cs group. In addition to these potential miRNA biomarkers for atherosclerotic processes mentioned in the literature, we were able to identify new candidates. For the aneu group miR-122-5p, miR-193a-5p, miR-543, miR-576-3p, and miR-629-5p were differentially expressed. miR-193a-5p, miR-370-3p, miR-409-3p, miR-493-3p, miR-495-3p and miR-543 were found for the chd group. In the cs group miR-193a-5p, miR-493-3p, miR-495-3p, miR-543 and miR-654-3p were found to be differentially expressed. And in the pad group miR-193a-5p, miR-199a-5p, miR-215-5p, miR-576-3p, miR-582-3p, miR-629-5p and miR-769-5p were found.

In addition to the results of the DGE analysis with more stringent filter criteria (log2FC ≥ | 1|, adjusted p-value ≤ 0.1 and base mean ≥ 50), further potential miRNA candidates were found using default filter settings (adjusted p-value ≤ 0.1). The relevance of these miRNAs to atherosclerotic processes is shown in the following by means of selected literature references. miR-22-5p and -3p were found to be differentially expressed in the comparison of all subgroups against the control. This miRNA is described to play a role in the formation of the neointima through the regulation of artery vascular SMCs (Huang et al., 2017; Yang et al., 2018). In functional *in-vitro* studies it could be shown that the reduced expression of miR-335-5p had a regulative effect on macrophages that was beneficial for plaque formation (Sun et al., 2021). In our case, this miRNA was expressed differentially compared to the aneu group. Other miRNAs such as miR-132 were associated with inducing proliferation in SMCs (Reddy et al., 2016) and were assigned in our study to the aneu group (miR-132-3p) (Supplementary Table 3). It is also believed that miR-21 targets TPM1. A downregulation of that gene is associated with the regulation of the shape of SMCs which influence the cytoskeletal stability (Wang et al., 2011). We found this miRNA (miR-21-5p) (Supplementary Table 3) to be upregulated in the comparison of the chd group to the control. Upregulated miR-191 is linked to the use of antiplatelet therapy (prasugrel or aspirin) (Willeit et al., 2013) and could be found in all groups (miR-193-3p) except the aneu.

The ORA results showed and confirmed the plausibility of the miRNAs found in relation to atherosclerotic processes. Several of the thereby identified biological processes that are triggered by mRNAs targeted by the specifically differentially regulated miRNAs found in our patients could be linked to the formation and progression of atherosclerosis. One example is the formation of atherosclerotic plaques. It is initiated by the sub endothelial accumulation of lipoproteins (Libby et al., 2019). Identified GO terms which are linked to this are describing the homeostasis, transport and metabolism of lipoproteins (positive regulation of cholesterol efflux, cholesterol homeostasis and positive regulation of lipid metabolic process). Subsequently, the accumulation of lipoproteins in the endothelium results in an immunological reaction. Immune cells and their messenger substances create inflammation (Linton and Fazio, 2003; Libby et al., 2010). Gene Ontology terms linked to this process refer on the one hand to cellular immune response such as the differentiation and regulation of leukocytes and t-cells and on the other to activation of messenger substances such as interleukins and growth factors. Furthermore, endothelial–mesenchymal transition plays an important role in the development of atherosclerosis (Souilhol et al., 2018; Wesseling et al., 2018; Hao et al., 2019), as do aging and mineralization (Menghini et al., 2009; Shioi and Ikari, 2018). The associated annotations of the ORA relate to the proliferation, differentiation and regulation of endothelial cells (e. g. *regulation of endothelial cell migration, endothelial cell activation*), but also generally to the blood vessel system and the remodelling of tissue (e.g., *regulation of tissue remodeling, regulation of vasculature development, vasculogenesis, artery development*). Interestingly, several annotations are linked to ossification as histologic process behind calcification of atherosclerotic plaques.

Samples from four sequencing runs were included in this study. Care was taken to achieve an approximately equal distribution of the groups for each run to avoid batch effects. It should be noted that no control group was available in the first run. Both the variation due to the groups within the individual sequencing and the sequencing runs itself were considered and corrected using a suitable algorithm of the DESeq2 package.

One limitation of this study is that the analysed miRNA is not a “pure EV miRNA,” as the EV precipitation method may also contain other miRNAs that are bound to other circulating co-isolates (Murillo et al., 2019). Co-isolates may include high- and low-density lipoproteins (HDL and LDL), Argonaut-2 protein complexes and other proteins binding circulating nucleic acids, including miRNAs. But as shown in previous studies, the EV precipitation methods result in the most abundant miRNA expression profiles with most stable biomarker signatures (Spornraft et al., 2014; Buschmann et al., 2016, 2018; Reithmair et al., 2017).

A further limitation of our study results from the fact that some of the identified miRNAs have low differential expression values between groups and that a second patient group for biologic validation of our findings was not available. Our study was designed as a hypothesis generating study, however, and aimed to identify only potential biomarkers for different vascular manifestations of systemic atherosclerosis and did not intend to present a full biomarker panel which need to be characterized in further studies. As any given miRNA can potentially regulate a high number of mRNA transcripts and even small differences in miRNA expression values can have large biologic consequences, we believe that some of the identified miRNAs may indeed be useful for screening patients with clinically suspected atherosclerosis based on the presence of easily identifiable risk factor (e.g., the metabolic syndrome).

## Conclusion

This study showed that different manifestations of atherosclerosis can be identified by differentiated miRNAs compared to the control group. In addition, group-specific miRNAs were found. The consistency of the results at the miRNA level is to be confirmed in a next step by additional differential mRNA expression analysis.

## Data Availability Statement

The miRNA-Seq. datasets generated and analyzed for this study can be found in https://www.ncbi.nlm.nih.gov/, with the accession number PRJNA739836.

## Ethics Statement

The studies involving human participants were reviewed and approved by Ethics Committee of the Medical Faculty of the University of Munich (protocol #17-572). The patients/participants provided their written informed consent to participate in this study.

## Author Contributions

MP, GS, and MR: initiating the research idea and applying for funding. FB, AD, RW, and AM: patient recruitment and metadata collection. AH, MR, and AL: laboratory processing of samples. BK, AH, and GD: bioinformatic processing and evaluation of sequencing data. AH: wrote the first draft of the manuscript. AM, GS, MP, and BK: support the interpretation and classification of results. All authors discussed the results and contributed to the final manuscript.

## Conflict of Interest

GD is employed by ecSeq Bioinformatics GmbH. The remaining authors declare that the research was conducted in the absence of any commercial or financial relationships that could be construed as a potential conflict of interest.

## Publisher’s Note

All claims expressed in this article are solely those of the authors and do not necessarily represent those of their affiliated organizations, or those of the publisher, the editors and the reviewers. Any product that may be evaluated in this article, or claim that may be made by its manufacturer, is not guaranteed or endorsed by the publisher.
