# Supplementary material for: Detection of Atherosclerosis by Small RNA-Sequencing Analysis of Extracellular Vesicle Enriched Serum Samples
Source: Front Cell Dev Biol. 2021 Oct 12;9:729061. doi: 10.3389/fcell.2021.729061 (PMC8546328; doi:10.3389/fcell.2021.729061)
Supplement: Supplementary file 1 [file Data_Sheet_1.docx]

**Supplementary material**

## Bioinformatical concepts and methods:

**Processing sequencing data**

Generated raw sequencing data was checked with FastQC (version 0.11.9) first. We evaluated the per base sequence quality which had to be above 30 for the first 28 nt. The overall phred score was expected to be above 30 and the proportion of undetermined bases had to be minimal. The sequence length distribution of our miRNAs study was expected to have a dominating peak at 21-22nt.

To generate read counts of individual miRNAs each dataset was processed as follows: First the adaptor sequences from the library preparation were removed in each sequence with btrim32 (version 0.3.0). Then, sequences with less than 16 nucleotides in length were removed from datasets. To quantify different RNA species, we performed a transcriptome mapping. References were downloaded from RNACentral (release 12) and miRBase (release 22.1). Mapping was done with bowtie (version 1.2.3). We mapped rRNA, snRNA, snoRNA, tRNA, rRNA and miRNA. The cut off for reads was set to maximum one mismatch. Mapping distribution of RNA species as well as other analytical plots and tables were generated with R (version 4.0.3; R Core Team, 2020) and scripts in awk (version 1.3.3, Nov 1996). The generated read counts for miRNAs were used for further analyses.

**Differential Gene Expression (DGE) analysis**

The expression profile of the control group was compared with different groups of atherosclerosis. To perform this, we used the DESeq2 (version 1.28.1) package for R. Potentially confounding factors such as different sequencing runs or library sizes were accounted by the mathematical model employed by DESeq2. The normalization of raw reads is carried out by calculating a sample specific scaling factor using the mean of ratio method. The significance of potential biomarker miRNAs was checked with a Wald-test. Since we tested multiple comparisons, we used the adjusted p-value as a criterion for rejecting our hypothesis. Significant miRNA candidates were filtered by adjusted p-value (≤0.1), |log_2_ fold change| (≥1) and the base mean expression rate (≥50 reads).

**Overlap analysis**

The overlap analysis was carried out to find group-specific differentially expressed miRNAs. For this we used the veccompare (version 0.1.0) package for R. As group-specific miRNAs were those that were present in only one group. Thereby the input for this analysis were miRNAs from the DGE analysis which resulted with stricter filter criteria (|log2FC| ≥ 1, adjusted p-value ≤ 0.1 and base mean ≥ 50). Additionally, we looked for miRNAs which were found exclusive in the individual groups. This means that they were not significantly regulated (padj. > 0.1) in other groups.

**Unsupervised and supervised clustering**

Unsupervised and supervised clustering are methods which analyse the expression data based on its inherent variance. If the variance between individual groups is big enough, clearly separated clusters can be visualized. In this context, unsupervised clustering differs from supervised clustering in the sense that no information about group membership is made in advance. For the unsupervised clustering, we performed a principal component analysis (PCA) using the DESeq2 (version 1.28.1), stats (version 4.0.3) and ggplot2 (version 3.3.2) packages for R. The PCA uses statistical techniques to determine key features of a high dimensional dataset. Thereby the variance within the datasets is determined in different components by reducing the dimensionality. By visualizing the components with a group specific colouring of the datasets it can be seen if the expression profile can be clustered group specific. Raw count data was first transformed using the variance stabilizing transformation algorithm included in DESeq2. Then the calculation was done by a singular value decomposition of the centered and scaled data.

Unsupervised clustering was carried out using the sparse partial least squares discriminant analysis (sPLS-DA). This method is used to enable the selection of the most discriminative features (miRNAs) in the data that help to classify data sets to a group. For this purpose, the data sets are assigned in advance to a group based on their clinical phenotype. We used the mixOmics (version 6.12.2) package for R to perform this analysis. The M-fold cross-validation subsets data sets into training and test sets to make a prediction on the subset left out of the calculation. During the cross-validation, this process is repeated several times because the results will be highly dependent on the random sub setting of data. To create a well fitted model, the optimal number of components and the optimal number of features (genes) need to be determined. The optimal number of components to display the variance of the data sets is determined by three distance measures (maximum, centroids and mahalanobis distance) and ranked with the balanced error rate. After determining the optimal number of components, the optimal number of features (miRNAs) for each component is calculated. This result can be used, to find potential miRNA biomarkers, which maximizes variance between groups.

**Overrepresentation analysis**

The overrepresentation analysis was carried out to find biological processes that were significantly enriched in targets (genes) of our differentially expressed miRNAs. We used the clusterProfiler (version 3.16.1) package for R to perform this analysis. First, we determined targets using the miRTarBase (release 8.0) database. To find biological processes that belong to the targets we used the Gene Ontology (GO) database. The overrepresentation analysis determines whether known biological processes are overrepresented in the target list of genes controlled by our found miRNAs. These biological processes are described as GO terms in the GO database. Finally, the GO terms found were assigned manually to disease specific processes.

Table S1: Percentage of the assigned read counts of the relative mapping distribution; No Adapter = reads without adapter; Short = reads smaller than 16 nt; Unmapped = reads that do not map to reference; rRNA = reads mapped as rRNA; snRNA = reads mapped as snRNA; snoRNA = reads mapped as snoRNA; tRNA = reads mapped as tRNA; miRNA = reads mapped as miRNA; athero. = atherosclerosis group; aneu = abdominal aneurysm; chd = coronary heart disease; cs = carotid stenosis; pad = peripheral artery disease; control = control group

|  | **[%] no adapter** | **[%] short** | **[%] unmapped** | **[%] rRNA** | **[%] snRNA** | **[%] snoRNA** | **[%] tRNA** | **[%] miRNA** |
| --- | --- | --- | --- | --- | --- | --- | --- | --- |
| aneu | 0.15 | 19.57 | 34.47 | 9.12 | 0.04 | 0.07 | 3.52 | 33.07 |
| chd | 0.13 | 23.13 | 39.78 | 9.11 | 0.03 | 0.04 | 4.37 | 23.41 |
| cs | 0.16 | 26.33 | 35.24 | 8.97 | 0.04 | 0.06 | 2.62 | 26.58 |
| pad | 0.11 | 18.87 | 40.53 | 11.73 | 0.04 | 0.06 | 4.78 | 23.89 |
| control | 0.00 | 25.52 | 30.94 | 12.34 | 0.03 | 0.04 | 6.50 | 24.64 |
| athero. | 0.14 | 21.74 | 37.72 | 9.77 | 0.04 | 0.06 | 3.91 | 26.62 |

Table S2: Percentage of the standard error of the assigned read counts of the relative mapping distribution; No Adapter = reads without adapter; Short = reads smaller than 16 nt; Unmapped = reads which are not mapped to reference; rRNA = reads mapped as rRNA; snRNA = reads mapped as snRNA; snoRNA = reads mapped as snoRNA; tRNA = reads mapped as tRNA; miRNA = reads mapped as miRNA; athero. = atherosclerosis group; aneu = abdominal aneurysm; chd = coronary heart disease; cs = carotid stenosis; pad = peripheral artery disease; control = control group

|  | **[%] no adapter** | **[%] short** | **[%] unmapped** | **[%] rRNA** | **[%] snRNA** | **[%] snoRNA** | **[%] tRNA** | **[%] miRNA** |
| --- | --- | --- | --- | --- | --- | --- | --- | --- |
| aneu | 0.04 | 2.99 | 4.76 | 1.53 | 0.01 | 0.01 | 1.58 | 4.82 |
| chd | 0.04 | 2.19 | 5.41 | 1.23 | 0.00 | 0.01 | 1.31 | 3.07 |
| cs | 0.05 | 2.73 | 5.51 | 1.41 | 0.00 | 0.01 | 0.81 | 4.30 |
| pad | 0.03 | 1.86 | 5.44 | 1.51 | 0.00 | 0.01 | 1.31 | 3.07 |
| volunteer | 0.00 | 3.80 | 3.31 | 1.18 | 0.00 | 0.00 | 0.84 | 2.64 |
| atherio. | 0.02 | 1.23 | 2.65 | 0.72 | 0.00 | 0.00 | 0.67 | 1.92 |

Table S3: DGE with default cut-offs of DESeq2 (adjusted p-value ≤ 0.1). Comparison of atherosclerosis group vs. control group. Log2FC = log2 fold change.

| **gene** | **Log2FC** | **p-value** | **p-adjusted-value** | **group mean atherosclerosis** | **group mean control** |
| --- | --- | --- | --- | --- | --- |
| let-7d-3p | -0.439 | 1.48E-02 | 9.63E-02 | 1708.84 | 2498.34 |
| let-7e-5p | -0.582 | 4.15E-03 | 4.45E-02 | 164.16 | 242.84 |
| miR-100-5p | 0.613 | 2.53E-03 | 3.25E-02 | 969.76 | 551.56 |
| miR-10399-3p | -0.474 | 9.89E-03 | 7.40E-02 | 14.22 | 15.88 |
| miR-10527-5p | 0.507 | 1.74E-03 | 2.65E-02 | 17.33 | 11.40 |
| miR-122-3p | 1.209 | 1.60E-02 | 9.84E-02 | 2.02 | 0.80 |
| miR-122-5p | 0.792 | 1.42E-02 | 9.44E-02 | 87668.46 | 60848.07 |
| miR-125a-3p | 1.348 | 9.17E-09 | 3.19E-06 | 28.20 | 10.72 |
| miR-1260b | -0.559 | 7.21E-03 | 5.97E-02 | 18.71 | 21.10 |
| miR-128-3p | -0.436 | 1.74E-04 | 5.26E-03 | 4722.18 | 6873.40 |
| miR-1285-3p | 0.424 | 1.54E-02 | 9.77E-02 | 43.46 | 35.92 |
| miR-1291 | 1.214 | 1.97E-03 | 2.79E-02 | 4.98 | 1.38 |
| miR-1299 | 2.007 | 1.83E-04 | 5.30E-03 | 50.52 | 13.15 |
| miR-1303 | 0.659 | 1.14E-02 | 8.16E-02 | 7.94 | 6.40 |
| miR-1306-5p | -1.040 | 9.89E-05 | 3.82E-03 | 3.61 | 7.62 |
| miR-130b-3p | 0.408 | 6.56E-03 | 5.68E-02 | 21.34 | 16.97 |
| miR-130b-5p | -0.676 | 2.78E-03 | 3.40E-02 | 137.46 | 241.54 |
| miR-146a-5p | 0.356 | 9.89E-03 | 7.40E-02 | 5051.87 | 3792.92 |
| miR-148a-3p | 0.436 | 1.10E-02 | 7.96E-02 | 137189.80 | 108424.17 |
| miR-151a-3p | -0.466 | 1.32E-02 | 9.03E-02 | 19684.31 | 29691.80 |
| miR-152-3p | 0.320 | 8.71E-03 | 6.97E-02 | 176.22 | 139.57 |
| miR-185-5p | 0.436 | 5.14E-03 | 5.26E-02 | 7261.71 | 5768.54 |
| miR-188-5p | 0.780 | 1.56E-02 | 9.77E-02 | 2.69 | 1.86 |
| miR-190b-5p | 1.060 | 7.91E-05 | 3.44E-03 | 6.84 | 2.80 |
| miR-191-3p | -0.619 | 2.13E-04 | 5.81E-03 | 11.64 | 18.89 |
| miR-193a-5p | 1.664 | 1.23E-11 | 8.55E-09 | 611.45 | 227.06 |
| miR-193b-5p | 1.362 | 1.54E-04 | 4.88E-03 | 45.29 | 24.53 |
| miR-194-5p | 0.581 | 3.45E-04 | 8.57E-03 | 756.73 | 628.98 |
| miR-195-5p | 0.768 | 6.37E-04 | 1.18E-02 | 7.96 | 5.24 |
| miR-199a-5p | -0.883 | 6.48E-05 | 3.01E-03 | 260.85 | 440.39 |
| miR-20b-5p | 0.534 | 6.69E-03 | 5.68E-02 | 68.04 | 51.20 |
| miR-21-5p | 0.319 | 1.38E-02 | 9.25E-02 | 15865.40 | 10874.77 |
| miR-2110 | 0.761 | 1.26E-04 | 4.61E-03 | 215.96 | 131.61 |
| miR-215-5p | -0.679 | 1.91E-03 | 2.77E-02 | 199.41 | 353.48 |
| miR-22-3p | 0.639 | 3.76E-06 | 4.36E-04 | 6017.18 | 4052.20 |
| miR-22-5p | 0.531 | 5.04E-05 | 3.01E-03 | 287.96 | 268.73 |
| miR-222-3p | 0.244 | 6.05E-03 | 5.49E-02 | 511.99 | 388.77 |
| miR-2355-3p | -0.501 | 8.98E-03 | 6.98E-02 | 12.42 | 17.00 |
| miR-26a-5p | -0.485 | 5.65E-03 | 5.46E-02 | 13730.85 | 17714.59 |
| miR-27a-5p | 1.045 | 1.76E-03 | 2.65E-02 | 275.68 | 168.02 |
| miR-27b-3p | 0.437 | 6.65E-03 | 5.68E-02 | 3138.62 | 2464.75 |
| miR-30d-5p | 0.333 | 8.26E-04 | 1.44E-02 | 7631.18 | 3578.75 |
| miR-3125 | 1.175 | 6.08E-03 | 5.49E-02 | 10.22 | 3.14 |
| miR-3127-5p | 0.963 | 1.19E-02 | 8.45E-02 | 1.65 | 0.76 |
| miR-3157-3p | 0.692 | 1.63E-02 | 9.98E-02 | 4.81 | 3.47 |
| miR-3168 | 1.581 | 1.35E-03 | 2.28E-02 | 4396.49 | 1452.08 |
| miR-320a-3p | 1.230 | 5.95E-06 | 4.60E-04 | 18484.90 | 10944.49 |
| miR-320b | 1.194 | 1.15E-05 | 8.00E-04 | 1087.52 | 554.24 |
| miR-320c | 0.795 | 5.43E-03 | 5.33E-02 | 333.22 | 147.25 |
| miR-320d | 1.505 | 1.22E-06 | 1.69E-04 | 188.06 | 76.94 |
| miR-320e | 1.573 | 2.17E-04 | 5.81E-03 | 6.87 | 1.38 |
| miR-323a-3p | -0.831 | 6.05E-03 | 5.49E-02 | 20.45 | 26.61 |
| miR-323a-5p | -1.331 | 2.61E-03 | 3.25E-02 | 1.48 | 3.97 |
| miR-329-3p | -0.924 | 1.24E-02 | 8.62E-02 | 7.54 | 11.98 |
| miR-335-3p | -0.689 | 3.78E-03 | 4.31E-02 | 99.00 | 170.29 |
| miR-338-5p | 0.601 | 1.39E-03 | 2.28E-02 | 93.45 | 58.88 |
| miR-339-3p | 0.515 | 5.27E-03 | 5.31E-02 | 107.28 | 81.13 |
| miR-340-3p | -0.592 | 6.63E-03 | 5.68E-02 | 71.95 | 104.94 |
| miR-3614-5p | 0.840 | 5.01E-03 | 5.21E-02 | 8.87 | 4.61 |
| miR-369-5p | -0.937 | 1.79E-03 | 2.65E-02 | 11.22 | 26.01 |
| miR-370-3p | -0.785 | 3.80E-03 | 4.31E-02 | 355.24 | 746.26 |
| miR-377-3p | -1.022 | 1.46E-02 | 9.58E-02 | 1.45 | 3.45 |
| miR-378a-3p | 0.980 | 5.30E-07 | 9.23E-05 | 1312.76 | 527.99 |
| miR-378c | 0.687 | 2.32E-03 | 3.10E-02 | 25.06 | 13.90 |
| miR-378d | 1.108 | 9.63E-05 | 3.82E-03 | 72.11 | 53.81 |
| miR-378i | 1.132 | 4.43E-06 | 4.40E-04 | 39.86 | 17.30 |
| miR-381-3p | -0.710 | 5.38E-03 | 5.33E-02 | 194.94 | 444.53 |
| miR-409-3p | -0.896 | 4.72E-04 | 9.95E-03 | 711.53 | 1112.69 |
| miR-4259 | 1.657 | 8.00E-03 | 6.48E-02 | 1.47 | 0.23 |
| miR-431-5p | -1.117 | 6.31E-04 | 1.18E-02 | 11.25 | 26.50 |
| miR-432-5p | -0.698 | 7.59E-03 | 6.21E-02 | 166.19 | 300.27 |
| miR-4433b-3p | -1.250 | 3.34E-04 | 8.57E-03 | 268.47 | 783.63 |
| miR-4433b-5p | -0.650 | 1.09E-02 | 7.96E-02 | 65.77 | 88.52 |
| miR-4446-3p | -0.628 | 9.19E-03 | 7.03E-02 | 34.46 | 51.56 |
| miR-452-5p | 0.713 | 5.72E-03 | 5.46E-02 | 20.52 | 13.77 |
| miR-455-5p | 1.087 | 3.88E-04 | 9.31E-03 | 19.78 | 11.84 |
| miR-483-5p | 1.349 | 4.11E-03 | 4.45E-02 | 110.32 | 69.93 |
| miR-485-3p | -1.259 | 4.21E-04 | 9.46E-03 | 10.14 | 26.51 |
| miR-485-5p | -0.861 | 2.27E-03 | 3.10E-02 | 39.25 | 72.37 |
| miR-493-3p | -0.857 | 3.94E-03 | 4.35E-02 | 82.06 | 198.07 |
| miR-495-3p | -1.002 | 4.20E-04 | 9.46E-03 | 47.03 | 109.77 |
| miR-500a-3p | 0.446 | 8.84E-03 | 6.98E-02 | 24.98 | 16.22 |
| miR-501-3p | 0.395 | 6.07E-03 | 5.49E-02 | 217.15 | 137.85 |
| miR-5010-5p | -0.864 | 9.03E-03 | 6.98E-02 | 4.21 | 7.99 |
| miR-502-3p | 0.437 | 2.14E-03 | 2.98E-02 | 56.27 | 46.66 |
| miR-543 | -1.041 | 6.35E-05 | 3.01E-03 | 203.74 | 500.62 |
| miR-548ad-5p=miR-548ae-5p | 0.810 | 6.45E-05 | 3.01E-03 | 19.99 | 14.29 |
| miR-548az-5p | 1.150 | 2.49E-03 | 3.25E-02 | 1.21 | 0.56 |
| miR-548d-5p | -0.844 | 6.78E-03 | 5.68E-02 | 1.60 | 2.42 |
| miR-574-5p | -1.670 | 1.47E-04 | 4.88E-03 | 19.97 | 66.40 |
| miR-576-3p | 1.243 | 5.57E-08 | 1.29E-05 | 117.95 | 52.28 |
| miR-589-5p | 0.603 | 5.59E-04 | 1.14E-02 | 36.14 | 21.81 |
| miR-615-3p | -1.027 | 7.13E-04 | 1.27E-02 | 20.26 | 40.64 |
| miR-625-5p | 0.687 | 5.74E-04 | 1.14E-02 | 7.81 | 5.51 |
| miR-629-5p | 0.942 | 5.43E-06 | 4.60E-04 | 862.94 | 453.26 |
| miR-654-3p | -0.716 | 6.64E-03 | 5.68E-02 | 106.29 | 187.35 |
| miR-654-5p | -0.877 | 2.59E-03 | 3.25E-02 | 30.47 | 48.06 |
| miR-664b-5p | -1.322 | 1.67E-03 | 2.63E-02 | 2.18 | 4.89 |
| miR-671-3p | -0.787 | 1.44E-04 | 4.88E-03 | 32.18 | 50.86 |
| miR-6785-5p | -1.461 | 1.35E-02 | 9.12E-02 | 0.98 | 2.78 |
| miR-6869-5p | 1.317 | 3.84E-03 | 4.31E-02 | 15.32 | 6.34 |
| miR-766-5p | -0.635 | 1.58E-02 | 9.84E-02 | 4.71 | 6.07 |
| miR-7854-3p | 0.675 | 1.55E-02 | 9.77E-02 | 5.19 | 3.04 |
| miR-877-5p | -0.891 | 3.24E-03 | 3.89E-02 | 5.39 | 11.96 |
| miR-885-5p | 1.449 | 6.20E-05 | 3.01E-03 | 15.46 | 7.36 |
| miR-9903 | -0.979 | 1.09E-02 | 7.96E-02 | 0.75 | 1.61 |
| miR-99a-5p | 0.485 | 1.26E-02 | 8.67E-02 | 10063.11 | 8525.33 |
| miR-216b-5p | 1.323 | 1.41E-03 | 2.28E-02 | 4.24 | 2.50 |
| miR-6850-5p | 2.183 | 4.47E-04 | 9.73E-03 | 5.48 | 0.29 |
| miR-3130-3p | 1.964 | 3.69E-03 | 4.31E-02 | 9.43 | 2.30 |
| miR-122b-5p | 1.175 | 1.53E-02 | 9.77E-02 | 1.91 | 0.85 |
| miR-5588-5p | 1.951 | 6.43E-04 | 1.18E-02 | 1.48 | 0.15 |
| miR-8485 | 1.606 | 1.20E-02 | 8.45E-02 | 2.49 | 0.25 |
| miR-194-3p | 1.811 | 4.55E-03 | 4.80E-02 | 1.83 | 0.06 |

Table S4: DGE with stricter filter criteria (adjusted p-value ≤ 0.1, |log_2_ fold change| ≥ 1, base mean ≥ 50). Comparison of atherosclerosis group vs. control group. Log2FC = log2 fold change. Grey-written miRNAs were not found under the filter criteria. They are used for statistical comparison with the other groups. Empty lines indicate that this miRNA was not expressed significantly different (padj. was smaller 0.1) in this comparison.

| **gene** | **Log2FC** | **p-value** | **p-adjusted-value** | **group mean atherosclerosis** | **group mean control** |
| --- | --- | --- | --- | --- | --- |
| miR-193a-5p | 1.66 | 1.23E-11 | 8.55E-09 | 611.5 | 227.1 |
| miR-27a-5p | 1.05 | 1.76E-03 | 2.65E-02 | 275.7 | 168 |
| miR-3168 | 1.58 | 1.35E-03 | 2.28E-02 | 4396.5 | 1452.1 |
| miR-320a-3p | 1.23 | 5.95E-06 | 4.60E-04 | 18484.9 | 10944.5 |
| miR-320b | 1.19 | 1.15E-05 | 8.00E-04 | 1087.5 | 554.2 |
| miR-320d | 1.51 | 1.22E-06 | 1.69E-04 | 188.1 | 76.9 |
| miR-378d | 1.11 | 9.63E-05 | 3.82E-03 | 72.1 | 53.8 |
| miR-4433b-3p | -1.25 | 3.34E-04 | 8.57E-03 | 268.5 | 783.6 |
| miR-483-5p | 1.35 | 4.11E-03 | 4.45E-02 | 110.3 | 69.9 |
| miR-495-3p | -1 | 4.20E-04 | 9.46E-03 | 47 | 109.8 |
| miR-543 | -1.04 | 6.35E-05 | 3.01E-03 | 203.7 | 500.6 |
| miR-576-3p | 1.24 | 5.57E-08 | 1.29E-05 | 117.9 | 52.3 |
|  |  |  |  |  |  |
| miR-122-5p | 0.792 | 1.42E-02 | 9.44E-02 | NA | NA |
| miR-199a-5p | -0.883 | 6.48E-05 | 3.01E-03 | NA | NA |
| miR-2110 | 0.761 | 1.26E-04 | 4.61E-03 | NA | NA |
| miR-215-5p | -0.679 | 1.91E-03 | 2.77E-02 | NA | NA |
| miR-320c | 0.795 | 5.43E-03 | 5.33E-02 | NA | NA |
| miR-335-3p | -0.689 | 3.78E-03 | 4.31E-02 | NA | NA |
| miR-370-3p | -0.785 | 3.80E-03 | 4.31E-02 | NA | NA |
| miR-381-3p | -0.710 | 5.38E-03 | 5.33E-02 | NA | NA |
| miR-409-3p | -0.896 | 4.72E-04 | 9.95E-03 | NA | NA |
| miR-493-3p | -0.857 | 3.94E-03 | 4.35E-02 | NA | NA |
| miR-493-5p | - | - | - | - | - |
| miR-582-3p | - | - | - | - | - |
| miR-629-5p | 0.942 | 5.43E-06 | 4.60E-04 | NA | NA |
| miR-654-3p | -0.716 | 6.64E-03 | 5.68E-02 | NA | NA |
| miR-769-5p | - | - | - | - | - |

Table S5: DGE with stricter filter criteria (adjusted p-value ≤ 0.1, |log_2_ fold change| ≥ 1, base mean ≥ 50). Comparison of abdominal aneurysm group vs. control group. Log2FC = log2 fold change. Grey-written miRNAs were not found under the filter criteria. They are used for statistical comparison with the other groups.Empty lines indicate that this miRNA was not expressed significantly differentiated (padj. was smaller 0.1) in this comparison.

| **gene** | **Log2FC** | **p-value** | **p-adjusted-value** | **group mean atherosclerosis** | **group mean control** |
| --- | --- | --- | --- | --- | --- |
| miR-122-5p | 1.1 | 5.43E-03 | 5.04E-02 | 108703.5 | 60848.1 |
| miR-193a-5p | 1.97 | 2.40E-11 | 1.25E-08 | 825.4 | 227.1 |
| miR-2110 | 1.03 | 1.87E-05 | 7.30E-04 | 233.9 | 131.6 |
| miR-27a-5p | 1.57 | 7.75E-05 | 1.92E-03 | 432.1 | 168 |
| miR-320a-3p | 1.41 | 2.11E-05 | 7.30E-04 | 17259.3 | 10944.5 |
| miR-320b | 1.51 | 4.45E-06 | 2.94E-04 | 1409.9 | 554.2 |
| miR-320c | 2 | 5.63E-07 | 5.85E-05 | 587 | 147.3 |
| miR-320d | 1.94 | 2.48E-07 | 3.22E-05 | 282.6 | 76.9 |
| miR-378d | 1.53 | 6.97E-06 | 4.03E-04 | 119.3 | 53.8 |
| miR-4433b-3p | -1.4 | 9.69E-04 | 1.68E-02 | 174.8 | 783.6 |
| miR-483-5p | 1.76 | 2.18E-03 | 2.70E-02 | 152.3 | 69.9 |
| miR-543 | -1.09 | 7.03E-04 | 1.26E-02 | 191.5 | 500.6 |
| miR-576-3p | 1.57 | 7.13E-09 | 1.24E-06 | 142.9 | 52.3 |
| miR-629-5p | 1.2 | 1.18E-06 | 1.02E-04 | 990.5 | 453.3 |
|  |  |  |  |  |  |
| miR-199-5p | -0.730 | 7.03e-03 | 6.09e-02 | NA | NA |
| miR-215-5p | -0.677 | 9.06e-03 | 7.36e-02 | NA | NA |
| miR-3168 | - | - | - | - | - |
| miR-335-3p | -0.828 | 4.00e-03 | 4.16e-02 | NA | NA |
| miR-370-3p | - | - | - | - | - |
| miR-381-3p | -0.782 | 1.08e-02 | 8.13e-02 | NA | NA |
| miR-409-3p | - | - | - | - | - |
| miR-493-3p | -0.910 | 1.16e-02 | 8.41e-02 | NA | NA |
| miR-493-5p | -0.921 | 1.47e-02 | 9.58e-02 | NA | NA |
| miR-495-3p | -0.897 | 1.03e-02 | 7.87e-02 | NA | NA |
| miR-582-3p | - | - | - | - | - |
| miR-654-3p | - | - | - | - | - |
| miR-769-5p | - | - | - | - | - |

Table S6: DGE with stricter filter criteria (adjusted p-value (Benjamini & Hochberg) ≤ 0.1, |log_2_ fold change| ≥ 1, base mean ≥ 50). Comparison of coronary heart disease group vs. control group. Log2FC = log2 fold change. Grey-written miRNAs were not found under the filter criteria. They are used for statistical comparison with the other groups. Empty lines indicate that this miRNA was not expressed significantly differentiated (padj. was smaller 0.1) in this comparison.

| **gene** | **Log2FC** | **p-value** | **p-adjusted-value** | **group mean atherosclerosis** | **group mean control** |
| --- | --- | --- | --- | --- | --- |
| miR-193a-5p | 1.23 | 1.93E-05 | 0.00289 | 409.7 | 227.1 |
| miR-320a-3p | 1.04 | 1.31E-03 | 0.02696 | 19718 | 10944.5 |
| miR-320b | 1 | 1.93E-03 | 0.03586 | 754 | 554.2 |
| miR-320d | 1.23 | 8.42E-04 | 0.01865 | 110.3 | 76.9 |
| miR-370-3p | -1.22 | 1.48E-04 | 0.0074 | 250.3 | 746.3 |
| miR-378d | 1.02 | 2.40E-03 | 0.03954 | 47.8 | 53.8 |
| miR-409-3p | -1.24 | 5.08E-05 | 0.00607 | 584.1 | 1112.7 |
| miR-493-3p | -1.22 | 5.39E-04 | 0.01666 | 56.5 | 198.1 |
| miR-495-3p | -1.16 | 7.12E-04 | 0.01793 | 41.4 | 109.8 |
| miR-543 | -1.09 | 5.57E-04 | 0.01666 | 189.2 | 500.6 |
|  |  |  |  |  |  |
| miR-122-5p | - | - | - | - | - |
| miR-199a-5p | -0.820 | 1.98e-03 | 0.03586 | NA | NA |
| miR-2110 | - | - | - | - | - |
| miR-215-5p | -0.988 | 1.04e-04 | 0.00740 | NA | NA |
| miR-27a-5p | - | - | - | - | - |
| miR-3168 | - | - | - | - | - |
| miR-320c | - | - | - | - | - |
| miR-335-3p | - | - | - | - | - |
| miR-381-3p | -0.921 | 2.19e-03 | 0.03745 | NA | NA |
| miR-4433b-3p | - | - | - | - | - |
| miR-483-5p | - | - | - | - | - |
| miR-493-5p | - | - | - | - | - |
| miR-576-3p | 0.924 | 5.40e-04 | 0.01666 | NA | NA |
| miR-582-3p | - | - | - | - | - |
| miR-629-5p | - | - | - | - | - |
| miR-654-3p | - | - | - | - | - |
| miR-769-5p | - | - | - | - | - |

Table S7: DGE with stricter filter criteria (adjusted p-value (Benjamini & Hochberg) ≤ 0.1, |log_2_ fold change| ≥ 1, base mean ≥ 50). Comparison of carotid stenosis group vs. control group. Log2FC = log2 fold change. Grey-written miRNAs were not found under the filter criteria. They are used for statistical comparison with the other groups. Empty lines indicate that this miRNA was not expressed significantly differentiated (padj. was smaller 0.1) in this comparison.

| **gene** | **Log2FC** | **p-value** | **p-adjusted-value** | **group mean atherosclerosis** | **group mean control** |
| --- | --- | --- | --- | --- | --- |
| miR-193a-5p | 1.36 | 7.11E-06 | 0.00542 | 475.3 | 227.1 |
| miR-27a-5p | 1.15 | 5.00E-03 | 0.08657 | 259.8 | 168 |
| miR-320d | 1.14 | 3.24E-03 | 0.06682 | 134.7 | 76.9 |
| miR-335-3p | -1.03 | 5.22E-04 | 0.03615 | 73.5 | 170.3 |
| miR-381-3p | -1.15 | 2.77E-04 | 0.03615 | 153.9 | 444.5 |
| miR-493-3p | -1.05 | 4.72E-03 | 0.08358 | 87.1 | 198.1 |
| miR-493-5p | -1.27 | 1.17E-03 | 0.04529 | 35.2 | 74.7 |
| miR-495-3p | -1.16 | 1.39E-03 | 0.04529 | 45.3 | 109.8 |
| miR-543 | -1.08 | 1.13E-03 | 0.04529 | 208.8 | 500.6 |
| miR-654-3p | -1.14 | 6.19E-04 | 0.03931 | 81.2 | 187.3 |
|  |  |  |  |  |  |
| miR-122-5p | - | - | - | - | - |
| miR-199a-5p | -0.833 | 2.84e-03 | 0.06362 | NA | NA |
| miR-2110 | - | - | - | - | - |
| miR-215-5p | - | - | - | - | - |
| miR-3168 | - | - | - | - | - |
| miR-320a-3p | - | - | - | - | - |
| miR-320b | - | - | - | - | - |
| miR-320c | - | - | - | - | - |
| miR-370-3p | -0.972 | 4.00e-03 | 0.07620 | NA | NA |
| miR-378d | - | - | - | - | - |
| miR-409-3p | -0.996 | 1.95e-03 | 0.05569 | NA | NA |
| miR-4433b-3p | - | - | - | - | - |
| miR-483-5p | - | - | - | - | - |
| miR-576-3p | 0.833 | 3.11e-03 | 0.06682 | NA | NA |
| miR-582-3p | - | - | - | - | - |
| miR-629-5p | - | - | - | - | - |
| miR-769-5p | - | - | - | - | - |

Table S8: D with stricter filter criteria (adjusted p-value (Benjamini & Hochberg) ≤ 0.1, |log_2_ fold change| ≥ 1, base mean ≥ 50). Comparison of peripheral arterial disease group vs. control group. Log2FC = log2 fold change. Grey-written miRNAs were not found under the filter criteria. They are used for statistical comparison with the other groups.Empty lines indicate that this miRNA was not expressed significantly differentiated (padj. was smaller 0.1) in this comparison.

| **gene** | **Log2FC** | **p-value** | **p-adjusted-value** | **group mean atherosclerosis** | **group mean control** |
| --- | --- | --- | --- | --- | --- |
| miR-193a-5p | 1.84 | 6.22E-10 | 3.98E-07 | 737.1 | 227.1 |
| miR-199a-5p | -1.14 | 3.31E-05 | 2.65E-03 | 196.9 | 440.4 |
| miR-215-5p | -1.01 | 1.27E-04 | 5.09E-03 | 157.3 | 353.5 |
| miR-3168 | 2.38 | 7.20E-05 | 4.19E-03 | 12795.9 | 1452.1 |
| miR-320a-3p | 1.49 | 7.07E-06 | 7.54E-04 | 24586.5 | 10944.5 |
| miR-320b | 1.31 | 8.15E-05 | 4.35E-03 | 1402.6 | 554.2 |
| miR-320c | 1.22 | 2.45E-03 | 4.49E-02 | 357.4 | 147.3 |
| miR-320d | 1.42 | 1.81E-04 | 6.51E-03 | 224.6 | 76.9 |
| miR-4433b-3p | -1.68 | 9.01E-05 | 4.44E-03 | 215.7 | 783.6 |
| miR-576-3p | 1.46 | 1.07E-07 | 3.41E-05 | 148.2 | 52.3 |
| miR-582-3p | 1.08 | 4.23E-04 | 1.13E-02 | 264.6 | 130.3 |
| miR-629-5p | 1.2 | 1.35E-06 | 2.15E-04 | 1057 | 453.3 |
| miR-769-5p | 1.01 | 3.36E-03 | 5.24E-02 | 156.6 | 57.3 |
|  |  |  |  |  |  |
| miR-122-5p | - | - | - | - | - |
| miR-2110 | 0.837 | 5.72e-04 | 1.36e-02 | NA | NA |
| miR-27a-5p | - | - | - | - | - |
| miR-335-3p | - | - | - | - | - |
| miR-370-3p | - | - | - | - | - |
| miR-378d | - | - | - | - | - |
| miR-381-3p | - | - | - | - | - |
| miR-409-3p | - | - | - | - | - |
| miR-483-5p | - | - | - | - | - |
| miR-493-3p | - | - | - | - | - |
| miR-493-5p | - | - | - | - | - |
| miR-495-3p | - | - | - | - | - |
| miR-543 | -0.944 | 3.60e-03 | 5.36e-02 | NA | NA |
| miR-654-3p | - | - | - | - | - |

Table S9: Uniquely differentially expressed miRNAs of the subgroups. Input for the overlap analysis was the miRNAs resulting from the DGE analysis with default cut-offs (adjusted p-value ≤ 0.1).

| **unique for coronial heart disease** | | | **unique for carotid stenosis** | | | **unique for abdominal aneurysm** | | | **unique for peripheral arteria disease** | | |
| --- | --- | --- | --- | --- | --- | --- | --- | --- | --- | --- | --- |
| **gene** | **log2FC** | **padj.** | **gene** | **log2FC** | **padj.** | **gene** | **log2FC** | **padj.** | **gene** | **log2FC** | **padj.** |
| **hsa-let-7d-5p** | 0.333 | 0.09381 | **hsa-miR-1229-3p** | -2.126 | 0.04529 | **hsa-let-7e-5p** | -0.799 | 1.94E-02 | **hsa-miR-106b-5p** | -0.543 | 7.21E-02 |
| **hsa-let-7e-3p** | 1.204 | 0.06587 | **hsa-miR-188-5p** | 1.183 | 0.06362 | **hsa-miR-122-5p** | 1.098 | 5.04E-02 | **hsa-miR-1260b** | -0.725 | 6.11E-02 |
| **hsa-miR-100-5p** | 0.758 | 0.03586 | **hsa-miR-222-3p** | 0.356 | 0.04529 | **hsa-miR-1277-5p** | -0.773 | 8.87E-02 | **hsa-miR-1285-3p** | 0.542 | 9.89E-02 |
| **hsa-miR-122-3p** | 1.598 | 0.06732 | **hsa-miR-3173-5p** | -1.186 | 0.03615 | **hsa-miR-1292-5p** | 0.689 | 5.90E-02 | **hsa-miR-1301-3p** | -0.594 | 3.39E-02 |
| **hsa-miR-1303** | 0.854 | 0.07254 | **hsa-miR-323a-3p** | -1.083 | 0.09108 | **hsa-miR-130b-3p** | 0.581 | 2.04E-02 | **hsa-miR-1307-3p** | 0.873 | 5.24E-02 |
| **hsa-miR-146b-5p** | -0.442 | 0.07131 | **hsa-miR-323a-5p** | -2.158 | 0.03615 | **hsa-miR-132-3p** | 0.842 | 9.58E-02 | **hsa-miR-148a-3p** | 0.632 | 4.58E-02 |
| **hsa-miR-200a-5p** | -1.137 | 0.06782 | **hsa-miR-329-3p** | -1.415 | 0.06362 | **hsa-miR-141-3p** | -1.083 | 3.16E-02 | **hsa-miR-151a-3p** | -0.693 | 4.58E-02 |
| **hsa-miR-203a-3p** | 0.927 | 0.07131 | **hsa-miR-377-3p** | -1.493 | 0.09726 | **hsa-miR-146a-5p** | 0.623 | 3.91E-03 | **hsa-miR-181a-5p** | 0.306 | 6.73E-02 |
| **hsa-miR-21-5p** | 0.476 | 0.03671 | **hsa-miR-411-3p** | -1.298 | 0.06144 | **hsa-miR-152-3p** | 0.402 | 6.09E-02 | **hsa-miR-181b-5p** | 0.442 | 2.18E-02 |
| **hsa-miR-27b-3p** | 0.694 | 0.01112 | **hsa-miR-6516-5p** | -1.215 | 0.04511 | **hsa-miR-193b-5p** | 1.935 | 4.53E-04 | **hsa-miR-182-5p** | 0.63 | 7.68E-02 |
| **hsa-miR-342-5p** | -0.788 | 0.03954 | **hsa-miR-654-3p** | -1.137 | 0.03931 | **hsa-miR-200a-3p** | -0.886 | 3.53E-02 | **hsa-miR-27a-3p** | 0.496 | 5.36E-02 |
| **hsa-miR-452-5p** | 1.135 | 0.0074 | **hsa-miR-6741-3p** | -0.928 | 0.09726 | **hsa-miR-20b-5p** | 0.739 | 2.65E-02 | **hsa-miR-3127-3p** | -1.214 | 5.57E-02 |
| **hsa-miR-548ar-3p** | 0.939 | 0.0946 | **hsa-miR-766-5p** | -0.931 | 0.09726 | **hsa-miR-218-5p** | -1.223 | 8.52E-03 | **hsa-miR-3127-5p** | 1.384 | 4.58E-02 |
| **hsa-miR-92a-1-5p** | -1.262 | 0.05456 | **hsa-miR-3940-5p** | 2.982 | 0.06894 | **hsa-miR-26a-5p** | -0.675 | 2.18E-02 | **hsa-miR-3168** | 2.380 | 4.19E-03 |
| **hsa-miR-216b-5p** | 1.804 | 0.0074 | **hsa-miR-3130-3p** | 2.620 | 0.05569 | **hsa-miR-30c-2-3p** | -1.382 | 6.09E-02 | **hsa-miR-326** | -0.615 | 6.73E-02 |
| **hsa-miR-194-3p** | 2.011 | 0.09511 | - | - | - | **hsa-miR-3157-3p** | 1.018 | 3.86E-02 | **hsa-miR-4306** | -0.831 | 9.74E-02 |
| - | - | - | - | - | - | **hsa-miR-335-5p** | 0.686 | 2.59E-02 | **hsa-miR-4433b-5p** | -0.99 | 3.28E-02 |
| - | - | - | - | - | - | **hsa-miR-339-3p** | 0.939 | 6.81E-04 | **hsa-miR-4446-3p** | -0.86 | 5.57E-02 |
| - | - | - | - | - | - | **hsa-miR-378c** | 0.95 | 1.06E-02 | **hsa-miR-450b-5p** | 0.742 | 5.88E-02 |
| - | - | - | - | - | - | **hsa-miR-411-5p** | -0.954 | 8.33E-02 | **hsa-miR-4791** | 1.315 | 8.55E-02 |
| - | - | - | - | - | - | **hsa-miR-412-5p** | 1.522 | 7.87E-02 | **hsa-miR-542-3p** | 0.686 | 9.89E-02 |
| - | - | - | - | - | - | **hsa-miR-423-5p** | 0.975 | 4.49E-02 | **hsa-miR-548az-5p** | 1.459 | 2.87E-02 |
| - | - | - | - | - | - | **hsa-miR-429** | -1.044 | 5.04E-02 | **hsa-miR-548d-5p** | -1.157 | 5.88E-02 |
| - | - | - | - | - | - | **hsa-miR-4443** | 1.395 | 9.58E-02 | **hsa-miR-548k** | 0.973 | 5.57E-02 |
| - | - | - | - | - | - | **hsa-miR-483-5p** | 1.765 | 2.70E-02 | **hsa-miR-581** | -0.913 | 9.89E-02 |
| - | - | - | - | - | - | **hsa-miR-500a-3p** | 0.52 | 8.53E-02 | **hsa-miR-582-3p** | 1.081 | 1.13E-02 |
| - | - | - | - | - | - | **hsa-miR-664a-5p** | 0.499 | 9.58E-02 | **hsa-miR-584-5p** | -0.362 | 9.00E-02 |
| - | - | - | - | - | - | **hsa-miR-7854-3p** | 0.895 | 6.77E-02 | **hsa-miR-642a-3p** | -0.977 | 7.21E-02 |
| - | - | - | - | - | - | **hsa-miR-204-3p** | 1.619 | 3.60E-02 | **hsa-miR-671-5p** | 1.261 | 8.55E-02 |
| - | - | - | - | - | - | - | - | - | **hsa-miR-6818-5p** | 1.410 | 8.09E-02 |
| - | - | - | - | - | - | - | - | - | **hsa-miR-769-5p** | 1.012 | 5.24E-02 |
| - | - | - | - | - | - | - | - | - | **hsa-miR-877-5p** | -1.495 | 4.78E-03 |
| - | - | - | - | - | - | - | - | - | **hsa-miR-93-3p** | -1.079 | 1.44E-02 |
| - | - | - | - | - | - | - | - | - | **hsa-miR-4738-3p** | 1.349 | 5.17E-02 |

Table S10: miRNAs used by the sPLS-DA to differentiate between atherosclerotic and control group; PC = principal component

| **gene** | **weighted coefficient** | **PC** |
| --- | --- | --- |
| miR-509-3-5p | -0.626 | PC1 |
| miR-193a-5p | 0.488 | PC1 |
| miR-1229-3p | -0.462 | PC1 |
| miR-125a-3p | 0.313 | PC1 |
| miR-1306-5p | -0.195 | PC1 |
| miR-22-3p | 0.096 | PC1 |
| miR-378a-3p | 0.086 | PC1 |
| miR-671-3p | -0.058 | PC1 |
| miR-30d-5p | -1.000 | PC2 |

Table S11: miRNAs used by the sPLS-DA to differentiate between all atherosclerotic subgroups and control group; PC = principal component

| **gene** | **weighted coefficient** | **PC** |
| --- | --- | --- |
| miR-125a-3p | 0.505 | PC1 |
| miR-193a-5p | 0.439 | PC1 |
| miR-576-3p | 0.241 | PC1 |
| miR-378a-3p | 0.205 | PC1 |
| miR-629-5p | 0.190 | PC1 |
| miR-22-3p | 0.110 | PC1 |
| miR-128-3p | -0.032 | PC1 |
| miR-199a-5p | -0.038 | PC1 |
| miR-615-3p | -0.052 | PC1 |
| miR-671-3p | -0.148 | PC1 |
| miR-1306-5p | -0.235 | PC1 |
| miR-1229-3p | -0.394 | PC1 |
| miR-509-3-5p | -0.408 | PC1 |
| miR-885-5p | 0.966 | PC2 |
| miR-27b-3p | 0.260 | PC2 |
| miR-215-5p | 0.608 | PC3 |
| miR-516a-5p | 0.429 | PC3 |
| miR-6815-3p | 0.347 | PC3 |
| miR-4707-3p | 0.317 | PC3 |
| miR-5589-3p | 0.226 | PC3 |
| miR-708-3p | 0.168 | PC3 |
| miR-574-5p | 0.075 | PC3 |
| miR-5588-3p | 0.064 | PC3 |
| miR-181a-2-3p | 0.059 | PC3 |
| miR-548ar-3p | -0.366 | PC3 |
| miR-181b-5p | 0.702 | PC4 |
| miR-124-3p | 0.526 | PC4 |
| miR-4804-3p | 0.294 | PC4 |
| miR-3908 | 0.262 | PC4 |
| miR-504-5p | 0.203 | PC4 |
| miR-6823-5p | 0.128 | PC4 |
| miR-3152-5p | 0.122 | PC4 |
| miR-4756-3p | 0.051 | PC4 |
| miR-4648 | 0.014 | PC4 |

Table S12: Result table of the overrepresentation analysis for the group atherosclerosis. GeneRatio: involved targeted genes/all targeted genes; BgRatio: number of genes in that GOterm/total number of genes in all GOterms; geneID: involved genes.

| **ID** | **Description** | **GeneRatio** | **BgRatio** | **p-value** | **p-adjusted-value** | **q-value** | **geneID** |
| --- | --- | --- | --- | --- | --- | --- | --- |
| GO:0060485 | **mesenchyme development** | 11/55 | 279/18670 | 4.55563E-10 | 3.29372E-07 | 1.5657E-07 | MTOR/WT1/SFRP1/GREM1/MYC/MAPK3/FOXC1/SOX9/HMGA2/NOS3/TWIST1 |
| GO:0046620 | **regulation of organ growth** | 8/55 | 113/18670 | 1.3466E-09 | 6.54248E-07 | 3.11003E-07 | MTOR/TP73/WT1/PPARA/AKT1/FOXC1/MEIS1/PTK2 |
| GO:0048015 | **phosphatidylinositol-mediated signaling** | 9/55 | 181/18670 | 2.68509E-09 | 7.85071E-07 | 3.7319E-07 | ERBB2/EGFR/RHOA/MAPK3/AKT1/SOX9/PTK2/TWIST1/SIRT1 |
| GO:0071902 | **positive regulation of protein serine/threonine kinase activity** | 11/55 | 334/18670 | 3.02233E-09 | 7.85071E-07 | 3.7319E-07 | ERBB2/TP73/EGFR/NOD2/RHOA/MAPK3/AKT1/HMGA2/KRAS/SIRT1/CCND1 |
| GO:2000027 | **regulation of animal organ morphogenesis** | 9/55 | 253/18670 | 4.93613E-08 | 3.66033E-06 | 1.73997E-06 | TFAP2A/WT1/SFRP1/GREM1/MYC/RHOA/FOXC1/SOX9/TWIST1 |
| GO:0071364 | **cellular response to epidermal growth factor stimulus** | 5/55 | 43/18670 | 1.62814E-07 | 1.00183E-05 | 4.76227E-06 | ERBB2/EGFR/AKT1/FOXC1/SOX9 |
| GO:0031998 | **regulation of fatty acid beta-oxidation** | 4/55 | 18/18670 | 1.99995E-07 | 1.15677E-05 | 5.49881E-06 | MTOR/PPARA/AKT1/TWIST1 |
| GO:0001558 | **regulation of cell growth** | 10/55 | 416/18670 | 3.23568E-07 | 1.671E-05 | 7.94324E-06 | ERBB2/MTOR/WT1/EGFR/PPARA/SFRP1/RHOA/SRF/AKT1/SIRT1 |
| GO:1901861 | **regulation of muscle tissue development** | 7/55 | 155/18670 | 3.44774E-07 | 1.74927E-05 | 8.31532E-06 | MTOR/TP73/NLN/PPARA/GREM1/MEIS1/TWIST1 |
| GO:0045926 | **negative regulation of growth** | 8/55 | 249/18670 | 6.34074E-07 | 2.82114E-05 | 1.34105E-05 | TP73/WT1/ING5/PPARA/SFRP1/MEIS1/PTK2/SIRT1 |
| GO:0051341 | **regulation of oxidoreductase activity** | 6/55 | 107/18670 | 7.10007E-07 | 3.06469E-05 | 1.45682E-05 | EGFR/NOD2/AKT1/ATP7A/KRAS/NOS3 |
| GO:0051052 | **regulation of DNA metabolic process** | 9/55 | 351/18670 | 7.86038E-07 | 3.29452E-05 | 1.56608E-05 | WT1/EGFR/GREM1/MYC/MAPK3/AKT1/HMGA2/TWIST1/SIRT1 |
| GO:1903706 | **regulation of hemopoiesis** | 10/55 | 475/18670 | 1.08376E-06 | 4.18638E-05 | 1.99003E-05 | ERBB2/MTOR/TP73/SFRP1/MYC/RBFOX2/RHOA/RUNX3/FOXC1/MEIS1 |
| GO:0045927 | **positive regulation of growth** | 8/55 | 270/18670 | 1.16626E-06 | 4.32412E-05 | 2.05551E-05 | ERBB2/MTOR/WT1/EGFR/SFRP1/RHOA/SRF/AKT1 |
| GO:0042698 | **ovulation cycle** | 5/55 | 69/18670 | 1.79368E-06 | 6.2498E-05 | 2.97089E-05 | EGFR/HSPA5/MMP7/NOS3/SIRT1 |
| GO:0071456 | **cellular response to hypoxia** | 7/55 | 207/18670 | 2.4054E-06 | 7.99589E-05 | 3.80092E-05 | MTOR/SFRP1/MYC/AKT1/ATP7A/TWIST1/SIRT1 |
| GO:0045792 | **negative regulation of cell size** | 3/55 | 10/18670 | 2.86084E-06 | 9.29614E-05 | 4.419E-05 | MTOR/RHOA/AKT1 |
| GO:1901214 | **regulation of neuron death** | 8/55 | 313/18670 | 3.50321E-06 | 0.000109187 | 5.19028E-05 | MTOR/TFAP2A/PPARA/RHOA/AKT1/CCL2/KRAS/SIRT1 |
| GO:1901342 | **regulation of vasculature development** | 9/55 | 422/18670 | 3.58082E-06 | 0.000109187 | 5.19028E-05 | ERBB2/WT1/SFRP1/RHOA/FOXC1/HMGA2/NOS3/TWIST1/SIRT1 |
| GO:0038083 | **peptidyl-tyrosine autophosphorylation** | 4/55 | 37/18670 | 4.14096E-06 | 0.000119757 | 5.69273E-05 | EGFR/GREM1/MAPK3/PTK2 |
| GO:0045834 | **positive regulation of lipid metabolic process** | 6/55 | 146/18670 | 4.36204E-06 | 0.000123677 | 5.87908E-05 | MTOR/PPARA/NOD2/AKT1/PTK2/TWIST1 |
| GO:0002683 | **negative regulation of immune system process** | 9/55 | 435/18670 | 4.58173E-06 | 0.000128644 | 6.11522E-05 | ERBB2/SFRP1/GREM1/MYC/AKT1/RUNX3/MEIS1/CCL2/SOX9 |
| GO:0001894 | **tissue homeostasis** | 7/55 | 229/18670 | 4.68805E-06 | 0.000130364 | 6.19696E-05 | EGFR/NOD2/SRF/FOXC1/SOX9/KRAS/NOS3 |
| GO:0031669 | **cellular response to nutrient levels** | 7/55 | 237/18670 | 5.87365E-06 | 0.000151666 | 7.20957E-05 | MTOR/SFRP1/TXN2/MAPK3/SRF/HSPA5/SIRT1 |
| GO:1902893 | **regulation of pri-miRNA transcription by RNA polymerase II** | 4/55 | 41/18670 | 6.29429E-06 | 0.000158288 | 7.52435E-05 | WT1/PPARA/SRF/SOX9 |
| GO:0043255 | **regulation of carbohydrate biosynthetic process** | 5/55 | 90/18670 | 6.69323E-06 | 0.000164041 | 7.79782E-05 | MTOR/NLN/PPARA/AKT1/SIRT1 |
| GO:0006352 | **DNA-templated transcription, initiation** | 7/55 | 249/18670 | 8.11307E-06 | 0.000188671 | 8.96862E-05 | PPARA/NOTCH3/MAPK3/SRF/SOX9/TWIST1/CCND1 |
| GO:0110149 | **regulation of biomineralization** | 5/55 | 94/18670 | 8.28533E-06 | 0.000188671 | 8.96862E-05 | TFAP2A/GREM1/SOX9/NOS3/TWIST1 |
| GO:0034614 | **cellular response to reactive oxygen species** | 6/55 | 168/18670 | 9.77E-06 | 0.000215686 | 0.000102528 | EGFR/MAPK3/AKT1/ATP7A/NOS3/SIRT1 |
| GO:0002042 | **cell migration involved in sprouting angiogenesis** | 5/55 | 98/18670 | 1.01595E-05 | 0.000217775 | 0.000103521 | PIK3R3/GREM1/RHOA/SRF/AKT1 |
| GO:0051147 | **regulation of muscle cell differentiation** | 6/55 | 181/18670 | 1.49362E-05 | 0.000284734 | 0.000135351 | MTOR/NLN/PPARA/GREM1/SRF/SIRT1 |
| GO:0003179 | **heart valve morphogenesis** | 4/55 | 52/18670 | 1.64271E-05 | 0.000298788 | 0.000142031 | MTOR/SOX9/NOS3/TWIST1 |
| GO:2000772 | **regulation of cellular senescence** | 4/55 | 52/18670 | 1.64271E-05 | 0.000298788 | 0.000142031 | HMGA2/KRAS/TWIST1/SIRT1 |
| GO:0010906 | **regulation of glucose metabolic process** | 5/55 | 112/18670 | 1.94525E-05 | 0.000340949 | 0.000162073 | MTOR/NLN/PPARA/AKT1/SIRT1 |
| GO:0090068 | **positive regulation of cell cycle process** | 7/55 | 298/18670 | 2.59037E-05 | 0.000423241 | 0.000201191 | TP73/EGFR/RHOA/AKT1/HMGA2/MTA3/CCND1 |
| GO:0031396 | **regulation of protein ubiquitination** | 6/55 | 204/18670 | 2.93492E-05 | 0.000464984 | 0.000221034 | MTOR/NOD2/AKT1/BMI1/HSPA5/MTA1 |
| GO:0001933 | **negative regulation of protein phosphorylation** | 8/55 | 429/18670 | 3.44187E-05 | 0.000525579 | 0.000249838 | MTOR/PEBP1/SFRP1/GREM1/MYC/AKT1/TWIST1/SIRT1 |
| GO:0009612 | **response to mechanical stimulus** | 6/55 | 210/18670 | 3.45297E-05 | 0.000525579 | 0.000249838 | EGFR/RHOA/MAPK3/AKT1/SOX9/MMP7 |
| GO:0055007 | **cardiac muscle cell differentiation** | 5/55 | 132/18670 | 4.28957E-05 | 0.00060811 | 0.00028907 | MTOR/WT1/PPARA/GREM1/SRF |
| GO:0045665 | **negative regulation of neuron differentiation** | 6/55 | 225/18670 | 5.07469E-05 | 0.000695545 | 0.000330633 | TP73/RHOA/NOTCH3/MEIS1/SOX9/PTK2 |
| GO:2000679 | **positive regulation of transcription regulatory region DNA binding** | 3/55 | 25/18670 | 5.31416E-05 | 0.000711507 | 0.000338221 | FOXC1/HMGA2/TWIST1 |
| GO:0062013 | **positive regulation of small molecule metabolic process** | 5/55 | 141/18670 | 5.87592E-05 | 0.000762025 | 0.000362235 | PPARA/AKT1/NOS3/TWIST1/SIRT1 |
| GO:1903959 | **regulation of anion transmembrane transport** | 3/55 | 26/18670 | 5.99479E-05 | 0.000769797 | 0.000365929 | MTOR/AKT1/ABCB1 |
| GO:0038127 | **ERBB signaling pathway** | 5/55 | 142/18670 | 6.07681E-05 | 0.000770795 | 0.000366404 | ERBB2/EGFR/AKT1/SOX9/PTK2 |
| GO:0097746 | **regulation of blood vessel diameter** | 5/55 | 143/18670 | 6.28297E-05 | 0.000788021 | 0.000374593 | EGFR/RHOA/AKT1/FOXC1/NOS3 |
| GO:0030217 | **T cell differentiation** | 6/55 | 240/18670 | 7.25697E-05 | 0.000881814 | 0.000419178 | ERBB2/MTOR/RHOA/SRF/ATP7A/RUNX3 |
| GO:0033688 | **regulation of osteoblast proliferation** | 3/55 | 28/18670 | 7.52198E-05 | 0.000895208 | 0.000425545 | SFRP1/GREM1/RHOA |
| GO:0048872 | **homeostasis of number of cells** | 6/55 | 246/18670 | 8.31582E-05 | 0.00095434 | 0.000453653 | RBFOX2/SRF/AKT1/SOX9/KRAS/NOS3 |
| GO:0043271 | **negative regulation of ion transport** | 5/55 | 157/18670 | 9.77686E-05 | 0.001058977 | 0.000503394 | MTOR/AKT1/ATP7A/NOS3/TWIST1 |
| GO:0043467 | **regulation of generation of precursor metabolites and energy** | 5/55 | 157/18670 | 9.77686E-05 | 0.001058977 | 0.000503394 | MTOR/PPARA/RHOA/AKT1/ATP7A |
| GO:0030198 | **extracellular matrix organization** | 7/55 | 368/18670 | 9.81748E-05 | 0.001059409 | 0.000503599 | WT1/GREM1/ATP7A/FOXC1/SOX9/MMP7/PTK2 |
| GO:0010975 | **regulation of neuron projection development** | 8/55 | 499/18670 | 9.93808E-05 | 0.001064756 | 0.000506141 | MTOR/SFRP1/SNAP25/RHOA/SRF/AKT1/HSPA5/PTK2 |
| GO:0048145 | **regulation of fibroblast proliferation** | 4/55 | 83/18670 | 0.000104213 | 0.001088024 | 0.000517201 | EGFR/SFRP1/MYC/BMI1 |
| GO:0060218 | **hematopoietic stem cell differentiation** | 4/55 | 83/18670 | 0.000104213 | 0.001088024 | 0.000517201 | TP73/SFRP1/SRF/FOXC1 |
| GO:0090257 | **regulation of muscle system process** | 6/55 | 259/18670 | 0.00011034 | 0.001131575 | 0.000537904 | MTOR/PPARA/RHOA/SRF/NOS3/PTK2 |
| GO:0043276 | **anoikis** | 3/55 | 34/18670 | 0.000135689 | 0.001353147 | 0.00064323 | MTOR/AKT1/PTK2 |
| GO:0060173 | **limb development** | 5/55 | 179/18670 | 0.00018078 | 0.001691959 | 0.000804287 | TFAP2A/GREM1/DLX5/SOX9/TWIST1 |
| GO:0071347 | **cellular response to interleukin-1** | 5/55 | 179/18670 | 0.00018078 | 0.001691959 | 0.000804287 | SFRP1/NOD2/MAPK3/CCL2/SOX9 |
| GO:2001022 | **positive regulation of response to DNA damage stimulus** | 4/55 | 97/18670 | 0.00019059 | 0.001733286 | 0.000823932 | EGFR/MYC/HMGA2/SIRT1 |
| GO:0048661 | **positive regulation of smooth muscle cell proliferation** | 4/55 | 101/18670 | 0.00022264 | 0.001933561 | 0.000919135 | MTOR/EGFR/NOTCH3/AKT1 |
| GO:0019233 | **sensory perception of pain** | 4/55 | 104/18670 | 0.000249101 | 0.00209419 | 0.000995491 | MTOR/MAPK3/CCL2/SMR3B |
| GO:2000377 | **regulation of reactive oxygen species metabolic process** | 5/55 | 195/18670 | 0.000268914 | 0.002228364 | 0.001059272 | MTOR/TFAP2A/EGFR/RHOA/AKT1 |
| GO:0002685 | **regulation of leukocyte migration** | 5/55 | 196/18670 | 0.00027534 | 0.002255761 | 0.001072295 | GREM1/RHOA/MAPK3/AKT1/CCL2 |
| GO:0050870 | **positive regulation of T cell activation** | 5/55 | 202/18670 | 0.000316385 | 0.002499958 | 0.001188376 | NOD2/RHOA/AKT1/RUNX3/CCL2 |
| GO:0052547 | **regulation of peptidase activity** | 7/55 | 452/18670 | 0.000346134 | 0.002676525 | 0.001272309 | PEBP1/MYC/RHOA/MAPK3/AKT1/SMR3B/SIRT1 |
| GO:2000109 | **regulation of macrophage apoptotic process** | 2/55 | 10/18670 | 0.000377682 | 0.002824642 | 0.001342717 | NOD2/SIRT1 |
| GO:0046850 | **regulation of bone remodeling** | 3/55 | 48/18670 | 0.000380916 | 0.002824642 | 0.001342717 | EGFR/SFRP1/GREM1 |
| GO:0007409 | **axonogenesis** | 7/55 | 468/18670 | 0.000426567 | 0.003077149 | 0.001462749 | ERBB2/DLX5/ALCAM/RHOA/MAPK3/SRF/PTK2 |
| GO:0045668 | **negative regulation of osteoblast differentiation** | 3/55 | 51/18670 | 0.000455781 | 0.003180086 | 0.001511681 | SFRP1/GREM1/TWIST1 |
| GO:0051496 | **positive regulation of stress fiber assembly** | 3/55 | 51/18670 | 0.000455781 | 0.003180086 | 0.001511681 | MTOR/SFRP1/RHOA |
| GO:1905477 | **positive regulation of protein localization to membrane** | 4/55 | 122/18670 | 0.000457556 | 0.003180086 | 0.001511681 | ERBB2/TP73/EGFR/AKT1 |
| GO:0045727 | **positive regulation of translation** | 4/55 | 127/18670 | 0.000532542 | 0.003598394 | 0.001710527 | ERBB2/MTOR/RHOA/MAPK3 |
| GO:0043551 | **regulation of phosphatidylinositol 3-kinase activity** | 3/55 | 55/18670 | 0.000569426 | 0.003717335 | 0.001767067 | PIK3R3/NOD2/PTK2 |
| GO:0043500 | **muscle adaptation** | 4/55 | 130/18670 | 0.000581516 | 0.003779203 | 0.001796476 | MTOR/PPARA/NOS3/PTK2 |
| GO:0045598 | **regulation of fat cell differentiation** | 4/55 | 132/18670 | 0.000615893 | 0.00394936 | 0.001877362 | MTOR/SFRP1/AKT1/SIRT1 |
| GO:0048588 | **developmental cell growth** | 5/55 | 234/18670 | 0.000618884 | 0.003959761 | 0.001882306 | MTOR/PPARA/ALCAM/SRF/SOX9 |
| GO:0033127 | **regulation of histone phosphorylation** | 2/55 | 13/18670 | 0.000650945 | 0.004119326 | 0.001958157 | MAPK3/TWIST1 |
| GO:0007050 | **cell cycle arrest** | 5/55 | 237/18670 | 0.000655561 | 0.004139481 | 0.001967738 | MTOR/TP73/MYC/HMGA2/CCND1 |
| GO:0031331 | **positive regulation of cellular catabolic process** | 6/55 | 362/18670 | 0.000663431 | 0.004175251 | 0.001984741 | PPARA/MAPK3/AKT1/PTK2/TWIST1/SIRT1 |
| GO:0051402 | **neuron apoptotic process** | 5/55 | 239/18670 | 0.000680903 | 0.004243905 | 0.002017376 | TFAP2A/RHOA/HSPA5/CCL2/KRAS |
| GO:0051893 | **regulation of focal adhesion assembly** | 3/55 | 60/18670 | 0.000735054 | 0.004465914 | 0.00212291 | SFRP1/RHOA/PTK2 |
| GO:0032922 | **circadian regulation of gene expression** | 3/55 | 62/18670 | 0.000809011 | 0.004804228 | 0.002283731 | PPARA/MTA1/SIRT1 |
| GO:0048857 | **neural nucleus development** | 3/55 | 62/18670 | 0.000809011 | 0.004804228 | 0.002283731 | RHOA/CKB/HSPA5 |
| GO:0040013 | **negative regulation of locomotion** | 6/55 | 381/18670 | 0.00086586 | 0.004989359 | 0.002371734 | SFRP1/GREM1/RHOA/SRF/AKT1/CCL2 |
| GO:0000098 | **sulfur amino acid catabolic process** | 2/55 | 15/18670 | 0.000872965 | 0.004989359 | 0.002371734 | TXN2/MAT1A |
| GO:0043518 | **negative regulation of DNA damage response, signal transduction by p53 class mediator** | 2/55 | 15/18670 | 0.000872965 | 0.004989359 | 0.002371734 | TWIST1/SIRT1 |
| GO:0051770 | **positive regulation of nitric-oxide synthase biosynthetic process** | 2/55 | 15/18670 | 0.000872965 | 0.004989359 | 0.002371734 | NOD2/CCL2 |
| GO:0071380 | **cellular response to prostaglandin E stimulus** | 2/55 | 16/18670 | 0.000995791 | 0.005495855 | 0.002612501 | SFRP1/AKT1 |
| GO:0035924 | **cellular response to vascular endothelial growth factor stimulus** | 3/55 | 68/18670 | 0.001058748 | 0.005777171 | 0.002746227 | AKT1/FOXC1/PTP4A3 |
| GO:0035855 | **megakaryocyte development** | 2/55 | 17/18670 | 0.001126433 | 0.006077695 | 0.002889084 | SRF/MEIS1 |
| GO:0019827 | **stem cell population maintenance** | 4/55 | 157/18670 | 0.001176021 | 0.006286602 | 0.00298839 | IGF2BP1/SFRP1/SOX9/HMGA2 |
| GO:0000082 | **G1/S transition of mitotic cell cycle** | 5/55 | 279/18670 | 0.001358812 | 0.007029849 | 0.003341699 | EGFR/MYC/AKT1/CCL2/CCND1 |
| GO:0032095 | **regulation of response to food** | 2/55 | 19/18670 | 0.001410984 | 0.007167222 | 0.003407 | MTOR/PPARA |
| GO:1902176 | **negative regulation of oxidative stress-induced intrinsic apoptotic signaling pathway** | 2/55 | 19/18670 | 0.001410984 | 0.007167222 | 0.003407 | AKT1/SIRT1 |
| GO:0090288 | **negative regulation of cellular response to growth factor stimulus** | 4/55 | 166/18670 | 0.001444369 | 0.007277203 | 0.00345928 | SFRP1/GREM1/HSPA5/SIRT1 |
| GO:0048844 | **artery morphogenesis** | 3/55 | 76/18670 | 0.00146068 | 0.00732112 | 0.003480157 | NOTCH3/SRF/FOXC1 |
| GO:0060828 | **regulation of canonical Wnt signaling pathway** | 5/55 | 286/18670 | 0.00151567 | 0.00752662 | 0.003577843 | EGFR/SFRP1/GREM1/DLX5/SOX9 |
| GO:0006809 | **nitric oxide biosynthetic process** | 3/55 | 77/18670 | 0.001516744 | 0.00752662 | 0.003577843 | MTOR/AKT1/NOS3 |
| GO:0002374 | **cytokine secretion involved in immune response** | 2/55 | 20/18670 | 0.001564803 | 0.007549621 | 0.003588777 | NOD2/MAPK3 |
| GO:0060216 | **definitive hemopoiesis** | 2/55 | 20/18670 | 0.001564803 | 0.007549621 | 0.003588777 | RBFOX2/MEIS1 |
| GO:0016241 | **regulation of macroautophagy** | 4/55 | 171/18670 | 0.001610588 | 0.007698875 | 0.003659726 | MTOR/MAPK3/AKT1/SIRT1 |
| GO:0071356 | **cellular response to tumor necrosis factor** | 5/55 | 291/18670 | 0.001635623 | 0.007767193 | 0.003692202 | SFRP1/MAPK3/AKT1/CCL2/SIRT1 |
| GO:0030728 | **ovulation** | 2/55 | 21/18670 | 0.001726257 | 0.008052153 | 0.00382766 | NOS3/SIRT1 |
| GO:0036499 | **PERK-mediated unfolded protein response** | 2/55 | 21/18670 | 0.001726257 | 0.008052153 | 0.00382766 | HSPA5/CCL2 |
| GO:2000738 | **positive regulation of stem cell differentiation** | 2/55 | 21/18670 | 0.001726257 | 0.008052153 | 0.00382766 | FOXC1/SOX9 |
| GO:0051222 | **positive regulation of protein transport** | 6/55 | 440/18670 | 0.001808606 | 0.008342088 | 0.003965482 | ERBB2/TP73/EGFR/NOD2/MAPK3/TWIST1 |
| GO:0006094 | **gluconeogenesis** | 3/55 | 82/18670 | 0.001817309 | 0.008342314 | 0.00396559 | NLN/PPARA/SIRT1 |
| GO:0010155 | **regulation of proton transport** | 2/55 | 22/18670 | 0.001895302 | 0.008524438 | 0.004052164 | ATP7A/TWIST1 |
| GO:0031281 | **positive regulation of cyclase activity** | 2/55 | 22/18670 | 0.001895302 | 0.008524438 | 0.004052164 | MAPK3/NOS3 |
| GO:0044321 | **response to leptin** | 2/55 | 22/18670 | 0.001895302 | 0.008524438 | 0.004052164 | SIRT1/CCND1 |
| GO:0010507 | **negative regulation of autophagy** | 3/55 | 84/18670 | 0.001947193 | 0.008663513 | 0.004118275 | MTOR/AKT1/PTK2 |
| GO:0021953 | **central nervous system neuron differentiation** | 4/55 | 183/18670 | 0.002063077 | 0.009092438 | 0.004322168 | SFRP1/DLX5/ATP7A/PTK2 |
| GO:0032331 | **negative regulation of chondrocyte differentiation** | 2/55 | 23/18670 | 0.002071894 | 0.009092438 | 0.004322168 | GREM1/SOX9 |
| GO:0048714 | **positive regulation of oligodendrocyte differentiation** | 2/55 | 23/18670 | 0.002071894 | 0.009092438 | 0.004322168 | MTOR/TP73 |
| GO:0001942 | **hair follicle development** | 3/55 | 86/18670 | 0.002082729 | 0.009126141 | 0.004338189 | EGFR/ATP7A/SOX9 |
| GO:0006112 | **energy reserve metabolic process** | 3/55 | 87/18670 | 0.002152643 | 0.0093335 | 0.004436759 | MTOR/MYC/AKT1 |
| GO:0044346 | **fibroblast apoptotic process** | 2/55 | 24/18670 | 0.002255987 | 0.009608712 | 0.004567583 | SFRP1/MYC |
| GO:0071674 | **mononuclear cell migration** | 3/55 | 90/18670 | 0.002371096 | 0.00998138 | 0.004744734 | GREM1/MAPK3/CCL2 |

Table S13: Result table of overrepresentation analysis for the abdominal aneurysm group compared to control group. GeneRatio: involved targeted genes/all targeted genes; BgRatio: number of genes in that GOterm/total number of genes in all GOterms; geneID: involved genes.

| **ID** | **Description** | **GeneRatio** | **BgRatio** | **p-value** | **p-adjusted-value** | **q-value** | **geneID** |
| --- | --- | --- | --- | --- | --- | --- | --- |
| GO:1901342 | **regulation of vasculature development** | 17/108 | 422/18670 | 3.19229E-10 | 1.53125E-07 | 9.192E-08 | ADAM10/RHOA/HMOX1/IL1A/MECP2/PKM/VEGFC/AKT3/SPRY2/ERBB2/WT1/SFRP1/XBP1/NOS3/TWIST1/HMGA2/SIRT1 |
| GO:0031100 | **animal organ regeneration** | 9/108 | 73/18670 | 3.68762E-10 | 1.53125E-07 | 9.192E-08 | AXL/CDK4/EGFR/HMOX1/PKM/WNT1/LPIN1/EZH2/CCND1 |
| GO:0031667 | **response to nutrient levels** | 18/108 | 499/18670 | 5.39666E-10 | 1.53125E-07 | 9.192E-08 | CREB1/EGFR/HMOX1/PDK4/PKM/SRF/NOD2/AACS/ACVR1C/MTOR/PPARA/SFRP1/TXN2/XBP1/MAPK3/MMP7/SIRT1/CCND1 |
| GO:1901653 | **cellular response to peptide** | 16/108 | 385/18670 | 7.09817E-10 | 1.76389E-07 | 1.05885E-07 | BCL2L2/CDK4/CREB1/IGF1R/PDK4/PKM/PTPN1/WNT1/SOCS1/LPIN1/NOD2/PIK3R3/SMARCC1/XBP1/PTK2/SIRT1 |
| GO:0071902 | **positive regulation of protein serine/threonine kinase activity** | 15/108 | 334/18670 | 8.77133E-10 | 2.03436E-07 | 1.22122E-07 | RHOA/EGFR/MAPK11/PTPN1/ADAM17/SPRY2/NOD2/ERBB2/TP73/EZH2/MAPK3/KRAS/HMGA2/SIRT1/CCND1 |
| GO:0007178 | **transmembrane receptor protein serine/threonine kinase signaling pathway** | 15/108 | 349/18670 | 1.60129E-09 | 3.27699E-07 | 1.96716E-07 | RHOA/CREB1/FUT8/ADAM17/WNT1/PEG10/ACVR1C/SFRP1/GREM1/DLX5/MAPK3/PTK2/SIRT1/HNF4A/TRIM33 |
| GO:0050708 | **regulation of protein secretion** | 17/108 | 472/18670 | 1.75473E-09 | 3.39151E-07 | 2.0359E-07 | EGFR/IL1A/MAPK11/RAC1/VEGFC/SOCS1/FOXP1/NOD2/AACS/RAB11FIP1/ACVR1C/SFRP1/SNAP25/XBP1/MAPK3/TWIST1/HNF4A |
| GO:1902105 | **regulation of leukocyte differentiation** | 13/108 | 272/18670 | 6.00879E-09 | 8.36184E-07 | 5.01956E-07 | RHOA/AXL/CREB1/SOCS1/TRIB1/FOXP1/ERBB2/MTOR/SFRP1/MYC/PRDM1/IRF4/XBP1 |
| GO:0030098 | **lymphocyte differentiation** | 14/108 | 353/18670 | 1.62032E-08 | 1.56586E-06 | 9.39976E-07 | RHOA/AXL/BAX/SRF/ADAM17/WNT1/SOCS1/FOXP1/ERBB2/MTOR/SFRP1/PRDM1/IRF4/XBP1 |
| GO:0051147 | **regulation of muscle cell differentiation** | 10/108 | 181/18670 | 9.74553E-08 | 7.70561E-06 | 4.62563E-06 | MECP2/MAPK11/SRF/MTOR/NLN/PPARA/GREM1/EZH2/XBP1/SIRT1 |
| GO:0048871 | **multicellular organismal homeostasis** | 15/108 | 486/18670 | 1.31005E-07 | 9.90795E-06 | 5.94768E-06 | BAX/EGFR/IGF1R/IL1A/PDK4/PRKAB1/RAC1/SRF/ADAM17/AKT3/LPIN1/NOD2/IRF4/KRAS/NOS3 |
| GO:0018108 | **peptidyl-tyrosine phosphorylation** | 13/108 | 363/18670 | 1.78501E-07 | 1.287E-05 | 7.72579E-06 | AXL/EGFR/IGF1R/PTPN1/ADAM17/SOCS1/DSTYK/ERBB2/MTOR/SFRP1/GREM1/MAPK3/PTK2 |
| GO:0001933 | **negative regulation of protein phosphorylation** | 14/108 | 429/18670 | 1.81268E-07 | 1.287E-05 | 7.72579E-06 | BAX/DUSP2/IGF1R/PTPN1/SOCS1/TRIB1/SPRY2/MTOR/PEBP1/SFRP1/GREM1/MYC/TWIST1/SIRT1 |
| GO:0048872 | **homeostasis of number of cells** | 11/108 | 246/18670 | 1.86194E-07 | 1.29553E-05 | 7.77701E-06 | AXL/BAX/HMOX1/SRF/ADAM17/AKT3/SLC7A11/EZH2/RBFOX2/KRAS/NOS3 |
| GO:0006352 | **DNA-templated transcription, initiation** | 11/108 | 249/18670 | 2.10297E-07 | 1.33022E-05 | 7.98525E-06 | BAX/CDK4/CREB1/MECP2/SRF/PPARA/NOTCH3/MAPK3/TWIST1/CCND1/HNF4A |
| GO:0071560 | **cellular response to transforming growth factor beta stimulus** | 11/108 | 249/18670 | 2.10297E-07 | 1.33022E-05 | 7.98525E-06 | RHOA/CREB1/FUT8/ADAM17/WNT1/PEG10/ACVR1C/SFRP1/PTK2/SIRT1/TRIM33 |
| GO:2000027 | **regulation of animal organ morphogenesis** | 11/108 | 253/18670 | 2.46719E-07 | 1.45481E-05 | 8.73311E-06 | RHOA/BAX/RAC1/WNT1/TFAP2A/WT1/SFRP1/GREM1/MYC/XBP1/TWIST1 |
| GO:0090288 | **negative regulation of cellular response to growth factor stimulus** | 9/108 | 166/18670 | 5.14903E-07 | 2.52022E-05 | 1.51287E-05 | PTPN1/ADAM17/WNT1/SPRY2/PEG10/SFRP1/GREM1/SIRT1/TRIM33 |
| GO:0048145 | **regulation of fibroblast proliferation** | 7/108 | 83/18670 | 5.1524E-07 | 2.52022E-05 | 1.51287E-05 | BAX/CDK4/CREB1/EGFR/WNT1/SFRP1/MYC |
| GO:2000106 | **regulation of leukocyte apoptotic process** | 7/108 | 83/18670 | 5.1524E-07 | 2.52022E-05 | 1.51287E-05 | AXL/BAX/ADAM17/SLC7A11/FOXP1/NOD2/SIRT1 |
| GO:0090344 | **negative regulation of cell aging** | 5/108 | 28/18670 | 5.21574E-07 | 2.52022E-05 | 1.51287E-05 | WNT1/AKT3/TWIST1/HMGA2/SIRT1 |
| GO:2001233 | **regulation of apoptotic signaling pathway** | 13/108 | 406/18670 | 6.37607E-07 | 2.95765E-05 | 1.77546E-05 | BAX/BCL2L1/BCL2L2/HMOX1/IL1A/PTPN1/PRKRA/TP73/ING5/SFRP1/XBP1/NOS3/SIRT1 |
| GO:0010594 | **regulation of endothelial cell migration** | 10/108 | 229/18670 | 8.58744E-07 | 3.78173E-05 | 2.27015E-05 | RHOA/HMOX1/MECP2/ADAM17/VEGFC/AKT3/FOXP1/NOS3/PTK2/SIRT1 |
| GO:0090068 | **positive regulation of cell cycle process** | 11/108 | 298/18670 | 1.24518E-06 | 5.09644E-05 | 3.05936E-05 | RHOA/BAX/CDK4/EGFR/IL1A/MECP2/ADAM17/TP73/EZH2/HMGA2/CCND1 |
| GO:0048010 | **vascular endothelial growth factor receptor signaling pathway** | 7/108 | 96/18670 | 1.39084E-06 | 5.56177E-05 | 3.3387E-05 | RHOA/AXL/MAPK11/PTPN1/RAC1/VEGFC/PTK2 |
| GO:0034599 | **cellular response to oxidative stress** | 11/108 | 302/18670 | 1.41804E-06 | 5.60611E-05 | 3.36531E-05 | AXL/EGFR/FUT8/HMOX1/WNT1/PRKRA/SLC7A11/EZH2/MAPK3/NOS3/SIRT1 |
| GO:1901861 | **regulation of muscle tissue development** | 8/108 | 155/18670 | 3.28465E-06 | 0.000113888 | 6.83664E-05 | CREB1/MAPK11/MTOR/TP73/NLN/PPARA/GREM1/TWIST1 |
| GO:0031668 | **cellular response to extracellular stimulus** | 10/108 | 268/18670 | 3.53548E-06 | 0.000113888 | 6.83664E-05 | AXL/HMOX1/PDK4/SRF/MTOR/SFRP1/TXN2/XBP1/MAPK3/SIRT1 |
| GO:0043523 | **regulation of neuron apoptotic process** | 9/108 | 210/18670 | 3.62819E-06 | 0.00011558 | 6.93818E-05 | RHOA/AXL/BAX/BCL2L1/HMOX1/MECP2/EGLN3/TFAP2A/KRAS |
| GO:0043491 | **protein kinase B signaling** | 10/108 | 269/18670 | 3.65444E-06 | 0.00011558 | 6.93818E-05 | AXL/EGFR/IGF1R/RAC1/SPRY2/ERBB2/MTOR/PIK3R3/PTK2/SIRT1 |
| GO:0007548 | **sex differentiation** | 10/108 | 270/18670 | 3.77685E-06 | 0.000117318 | 7.04255E-05 | AXL/BAX/BCL2L1/BCL2L2/WT1/SFRP1/NOS3/SIRT1/CCND1/HNF4A |
| GO:0045927 | **positive regulation of growth** | 10/108 | 270/18670 | 3.77685E-06 | 0.000117318 | 7.04255E-05 | ADAM10/RHOA/CREB1/EGFR/SRF/ADAM17/ERBB2/MTOR/WT1/SFRP1 |
| GO:0043200 | **response to amino acid** | 7/108 | 113/18670 | 4.15826E-06 | 0.000126022 | 7.56503E-05 | RHOA/BCL2L1/CREB1/EGFR/SOCS1/MTOR/XBP1 |
| GO:0061448 | **connective tissue development** | 10/108 | 273/18670 | 4.16572E-06 | 0.000126022 | 7.56503E-05 | CDK4/MEF2D/SRF/AACS/WT1/GREM1/XBP1/MAPK3/HMGA2/SIRT1 |
| GO:0060348 | **bone development** | 9/108 | 217/18670 | 4.74007E-06 | 0.000138577 | 8.31871E-05 | RHOA/MEF2D/SRF/WNT1/FOXP1/TFAP2A/GREM1/DLX5/TWIST1 |
| GO:0060485 | **mesenchyme development** | 10/108 | 279/18670 | 5.0489E-06 | 0.000143976 | 8.6428E-05 | MTOR/WT1/SFRP1/GREM1/MYC/EZH2/MAPK3/NOS3/TWIST1/HMGA2 |
| GO:0001558 | **regulation of cell growth** | 12/108 | 416/18670 | 5.18039E-06 | 0.000146525 | 8.79579E-05 | ADAM10/RHOA/EGFR/SRF/ADAM17/ERBB2/MTOR/WT1/PPARA/SFRP1/SIRT1/HNF4A |
| GO:0042471 | **ear morphogenesis** | 7/108 | 118/18670 | 5.54395E-06 | 0.000155543 | 9.33717E-05 | WNT1/PRKRA/SPRY2/TFAP2A/DLX5/MAPK3/TWIST1 |
| GO:0090398 | **cellular senescence** | 6/108 | 78/18670 | 5.9676E-06 | 0.000163475 | 9.81327E-05 | SRF/AKT3/KRAS/TWIST1/HMGA2/SIRT1 |
| GO:0060249 | **anatomical structure homeostasis** | 12/108 | 439/18670 | 8.92503E-06 | 0.000215626 | 0.000129439 | ALDOA/BAX/EGFR/PDK4/RAC1/SRF/AKT3/NOD2/MYC/MAPK3/KRAS/NOS3 |
| GO:0051222 | **positive regulation of protein transport** | 12/108 | 440/18670 | 9.13139E-06 | 0.000218959 | 0.00013144 | EGFR/IL1A/MAPK11/RAC1/VEGFC/NOD2/AACS/ERBB2/TP73/XBP1/MAPK3/TWIST1 |
| GO:0035094 | **response to nicotine** | 5/108 | 49/18670 | 9.18885E-06 | 0.000218959 | 0.00013144 | ATP1A2/CREB1/HMOX1/SLC7A11/PPARA |
| GO:0044282 | **small molecule catabolic process** | 12/108 | 445/18670 | 1.02272E-05 | 0.000236571 | 0.000142012 | ALDOA/CYP7A1/FUT8/PKM/ENTPD4/LPIN1/NT5C3A/MTOR/PPARA/TXN2/NOS3/TWIST1 |
| GO:0002040 | **sprouting angiogenesis** | 8/108 | 183/18670 | 1.11664E-05 | 0.000252259 | 0.000151429 | RHOA/HMOX1/PKM/SRF/VEGFC/AKT3/PIK3R3/GREM1 |
| GO:0045926 | **negative regulation of growth** | 9/108 | 249/18670 | 1.43381E-05 | 0.000300496 | 0.000180386 | MAPK11/TP73/WT1/ING5/PPARA/SFRP1/PTK2/SIRT1/HNF4A |
| GO:1901655 | **cellular response to ketone** | 6/108 | 93/18670 | 1.65104E-05 | 0.000333953 | 0.00020047 | CDK4/EGFR/AACS/SFRP1/GNAI1/SIRT1 |
| GO:0001763 | **morphogenesis of a branching structure** | 8/108 | 196/18670 | 1.83657E-05 | 0.000360985 | 0.000216697 | SRF/WNT1/SPRY2/WT1/SFRP1/MYC/PRDM1/KRAS |
| GO:0002685 | **regulation of leukocyte migration** | 8/108 | 196/18670 | 1.83657E-05 | 0.000360985 | 0.000216697 | ADAM10/RHOA/HMOX1/RAC1/ADAM17/VEGFC/GREM1/MAPK3 |
| GO:0019217 | **regulation of fatty acid metabolic process** | 6/108 | 96/18670 | 1.98006E-05 | 0.000382702 | 0.000229734 | CYP7A1/PDK4/MTOR/PPARA/TWIST1/SIRT1 |
| GO:0055081 | **anion homeostasis** | 5/108 | 58/18670 | 2.11868E-05 | 0.000407231 | 0.000244458 | SLC7A11/XBP1/CKB/SIRT1/HNF4A |
| GO:0046883 | **regulation of hormone secretion** | 9/108 | 262/18670 | 2.14591E-05 | 0.000410199 | 0.00024624 | CREB1/EGFR/RAC1/AACS/RAB11FIP1/ACVR1C/SFRP1/SNAP25/HNF4A |
| GO:0002573 | **myeloid leukocyte differentiation** | 8/108 | 204/18670 | 2.44886E-05 | 0.000448398 | 0.000269171 | CREB1/TRIB1/FOXP1/MTOR/SFRP1/MYC/IRF4/SIRT1 |
| GO:0048661 | **positive regulation of smooth muscle cell proliferation** | 6/108 | 101/18670 | 2.6444E-05 | 0.000464639 | 0.00027892 | EGFR/HMOX1/MEF2D/FOXP1/MTOR/NOTCH3 |
| GO:0016051 | **carbohydrate biosynthetic process** | 8/108 | 208/18670 | 2.81409E-05 | 0.000491971 | 0.000295327 | ALDOA/FUT8/GYS1/G6PC3/MTOR/NLN/PPARA/SIRT1 |
| GO:0070059 | **intrinsic apoptotic signaling pathway in response to endoplasmic reticulum stress** | 5/108 | 62/18670 | 2.93767E-05 | 0.000508465 | 0.000305228 | BAX/BCL2L1/PTPN1/XBP1/SIRT1 |
| GO:0048638 | **regulation of developmental growth** | 10/108 | 347/18670 | 3.33406E-05 | 0.000563068 | 0.000338006 | CDK4/CREB1/MAPK11/SRF/MTOR/TP73/WT1/PPARA/SFRP1/PTK2 |
| GO:0033028 | **myeloid cell apoptotic process** | 4/108 | 33/18670 | 3.8076E-05 | 0.000621908 | 0.000373327 | ADAM17/SLC7A11/NOD2/SIRT1 |
| GO:0042063 | **gliogenesis** | 9/108 | 290/18670 | 4.74525E-05 | 0.000746511 | 0.000448125 | RHOA/CREB1/EGFR/ERBB2/MTOR/TP73/EZH2/MAPK3/KRAS |
| GO:0042698 | **ovulation cycle** | 5/108 | 69/18670 | 4.94088E-05 | 0.000763969 | 0.000458606 | AXL/EGFR/MMP7/NOS3/SIRT1 |
| GO:1900103 | **positive regulation of endoplasmic reticulum unfolded protein response** | 3/108 | 13/18670 | 5.1615E-05 | 0.000791051 | 0.000474863 | BAX/PTPN1/XBP1 |
| GO:0042752 | **regulation of circadian rhythm** | 6/108 | 114/18670 | 5.23706E-05 | 0.000795622 | 0.000477606 | CREB1/MTOR/PPARA/EZH2/MTA1/HNF4A |
| GO:0071216 | **cellular response to biotic stimulus** | 8/108 | 236/18670 | 6.87688E-05 | 0.000996861 | 0.000598409 | RHOA/AXL/CDK4/TRIB1/NOD2/XBP1/MAPK3/NOS3 |
| GO:0007050 | **cell cycle arrest** | 8/108 | 237/18670 | 7.08337E-05 | 0.001022532 | 0.000613819 | BAX/CDK4/PRKAB1/MTOR/TP73/MYC/HMGA2/CCND1 |
| GO:0050663 | **cytokine secretion** | 8/108 | 240/18670 | 7.734E-05 | 0.001107267 | 0.000664685 | IL1A/MAPK11/SOCS1/FOXP1/NOD2/XBP1/MAPK3/TWIST1 |
| GO:0008361 | **regulation of cell size** | 7/108 | 179/18670 | 8.17785E-05 | 0.001147207 | 0.000688661 | RHOA/CDK4/CREB1/RAC1/SRF/AKT3/MTOR |
| GO:0060173 | **limb development** | 7/108 | 179/18670 | 8.17785E-05 | 0.001147207 | 0.000688661 | BAX/PRKAB1/SLC7A11/TFAP2A/GREM1/DLX5/TWIST1 |
| GO:0007409 | **axonogenesis** | 11/108 | 468/18670 | 8.45675E-05 | 0.00116915 | 0.000701833 | RHOA/CREB1/NCAM1/RAC1/SRF/NUMBL/ERBB2/DLX5/ALCAM/MAPK3/PTK2 |
| GO:0008217 | **regulation of blood pressure** | 7/108 | 182/18670 | 9.07573E-05 | 0.001238214 | 0.000743292 | RHOA/ATP1A2/HMOX1/MECP2/VEGFC/PPARA/NOS3 |
| GO:0043280 | **positive regulation of cysteine-type endopeptidase activity involved in apoptotic process** | 6/108 | 132/18670 | 0.000118216 | 0.001540356 | 0.000924666 | RHOA/BAX/EGLN3/ACVR1C/MYC/SIRT1 |
| GO:0045598 | **regulation of fat cell differentiation** | 6/108 | 132/18670 | 0.000118216 | 0.001540356 | 0.000924666 | CREB1/WNT1/MTOR/SFRP1/XBP1/SIRT1 |
| GO:0046677 | **response to antibiotic** | 9/108 | 327/18670 | 0.00011887 | 0.001543097 | 0.000926311 | RHOA/AXL/BCL2L1/HMOX1/AACS/EZH2/GNAI1/SIRT1/CCND1 |
| GO:0071333 | **cellular response to glucose stimulus** | 6/108 | 133/18670 | 0.000123226 | 0.001581928 | 0.000949621 | CYP7A1/IGF1R/RAC1/SRF/AACS/XBP1 |
| GO:0090257 | **regulation of muscle system process** | 8/108 | 259/18670 | 0.000131126 | 0.001652858 | 0.0009922 | ANK2/RHOA/ATP1A2/SRF/MTOR/PPARA/NOS3/PTK2 |
| GO:0030336 | **negative regulation of cell migration** | 9/108 | 334/18670 | 0.000139433 | 0.001726293 | 0.001036283 | RHOA/HMOX1/MECP2/SRF/TRIB1/CLIC4/ACVR1C/SFRP1/GREM1 |
| GO:0034103 | **regulation of tissue remodeling** | 5/108 | 88/18670 | 0.000157883 | 0.001855653 | 0.001113937 | BAX/EGFR/PDK4/SFRP1/GREM1 |
| GO:0035265 | **organ growth** | 7/108 | 204/18670 | 0.000184192 | 0.002108664 | 0.001265817 | MAPK11/SPRY2/MTOR/TP73/WT1/PPARA/PTK2 |
| GO:0071456 | **cellular response to hypoxia** | 7/108 | 207/18670 | 0.000201453 | 0.002232021 | 0.001339868 | HMOX1/EGLN3/MTOR/SFRP1/MYC/TWIST1/SIRT1 |
| GO:0051496 | **positive regulation of stress fiber assembly** | 4/108 | 51/18670 | 0.00021466 | 0.002348436 | 0.001409751 | RHOA/RAC1/MTOR/SFRP1 |
| GO:1990830 | **cellular response to leukemia inhibitory factor** | 5/108 | 96/18670 | 0.000237489 | 0.00252668 | 0.00151675 | CREB1/VEGFC/SPRY2/XBP1/SIRT1 |
| GO:0051348 | **negative regulation of transferase activity** | 8/108 | 285/18670 | 0.000251643 | 0.002669108 | 0.001602248 | DUSP2/IGF1R/PTPN1/SOCS1/TRIB1/SPRY2/SFRP1/SIRT1 |
| GO:0031331 | **positive regulation of cellular catabolic process** | 9/108 | 362/18670 | 0.0002539 | 0.002676726 | 0.001606821 | BAX/HMOX1/PTPN1/TRIB1/PPARA/MAPK3/PTK2/TWIST1/SIRT1 |
| GO:0006925 | **inflammatory cell apoptotic process** | 3/108 | 22/18670 | 0.000267585 | 0.002781805 | 0.001669899 | SLC7A11/NOD2/SIRT1 |
| GO:0043551 | **regulation of phosphatidylinositol 3-kinase activity** | 4/108 | 55/18670 | 0.000287809 | 0.002936329 | 0.001762659 | SOCS1/NOD2/PIK3R3/PTK2 |
| GO:0016573 | **histone acetylation** | 6/108 | 156/18670 | 0.000293246 | 0.002974348 | 0.001785482 | MECP2/ING5/IRF4/MAPK3/TWIST1/SIRT1 |
| GO:0001764 | **neuron migration** | 6/108 | 157/18670 | 0.000303482 | 0.003049231 | 0.001830433 | AXL/BAX/RAC1/SRF/PTK2/TWIST1 |
| GO:0089718 | **amino acid import across plasma membrane** | 3/108 | 23/18670 | 0.000306431 | 0.003054652 | 0.001833688 | ATP1A2/SLC7A1/SLC7A11 |
| GO:0071398 | **cellular response to fatty acid** | 4/108 | 57/18670 | 0.000330398 | 0.003247049 | 0.001949182 | CDK4/CREB1/PDK4/SFRP1 |
| GO:0060343 | **trabecula formation** | 3/108 | 24/18670 | 0.000348737 | 0.003398473 | 0.002040081 | SRF/SFRP1/GREM1 |
| GO:0001819 | **positive regulation of cytokine production** | 10/108 | 464/18670 | 0.000359714 | 0.003485922 | 0.002092577 | CREB1/HMOX1/IL1A/MAPK11/ADAM17/NOD2/IRF4/XBP1/MAPK3/TWIST1 |
| GO:0000302 | **response to reactive oxygen species** | 7/108 | 232/18670 | 0.00040226 | 0.003754018 | 0.002253512 | AXL/EGFR/HMOX1/EZH2/MAPK3/NOS3/SIRT1 |
| GO:0071695 | **anatomical structure maturation** | 7/108 | 232/18670 | 0.00040226 | 0.003754018 | 0.002253512 | RHOA/AXL/MECP2/WNT1/MTOR/GREM1/XBP1 |
| GO:0051893 | **regulation of focal adhesion assembly** | 4/108 | 60/18670 | 0.000402486 | 0.003754018 | 0.002253512 | RHOA/RAC1/SFRP1/PTK2 |
| GO:0009895 | **negative regulation of catabolic process** | 8/108 | 308/18670 | 0.000422929 | 0.003897798 | 0.002339823 | EGFR/HMOX1/WNT1/MTOR/IGF2BP1/PPARA/SMARCC1/PTK2 |
| GO:0003170 | **heart valve development** | 4/108 | 61/18670 | 0.000428827 | 0.003915716 | 0.002350579 | MTOR/PRDM1/NOS3/TWIST1 |
| GO:0072330 | **monocarboxylic acid biosynthetic process** | 7/108 | 237/18670 | 0.000457041 | 0.004066615 | 0.002441163 | CYP7A1/PDK4/PKM/PRKAB1/SRR/XBP1/SIRT1 |
| GO:0050863 | **regulation of T cell activation** | 8/108 | 314/18670 | 0.000480512 | 0.004211212 | 0.002527963 | RHOA/RAC1/SOCS1/NOD2/ERBB2/PRDM1/IRF4/XBP1 |
| GO:0010906 | **regulation of glucose metabolic process** | 5/108 | 112/18670 | 0.000484509 | 0.004219697 | 0.002533057 | PDK4/MTOR/NLN/PPARA/SIRT1 |
| GO:0022407 | **regulation of cell-cell adhesion** | 9/108 | 402/18670 | 0.000544274 | 0.004607124 | 0.002765626 | RHOA/RAC1/WNT1/SOCS1/NOD2/ERBB2/PPARA/XBP1/PTK2 |
| GO:0033688 | **regulation of osteoblast proliferation** | 3/108 | 28/18670 | 0.000555044 | 0.004637668 | 0.002783962 | RHOA/SFRP1/GREM1 |
| GO:2000045 | **regulation of G1/S transition of mitotic cell cycle** | 6/108 | 184/18670 | 0.000703647 | 0.005679785 | 0.003409538 | BAX/CDK4/EGFR/ADAM17/EZH2/CCND1 |
| GO:0044319 | **wound healing, spreading of cells** | 3/108 | 31/18670 | 0.000752038 | 0.005919323 | 0.003553331 | RHOA/ADAM17/MTOR |
| GO:1905475 | **regulation of protein localization to membrane** | 6/108 | 187/18670 | 0.000765643 | 0.005999265 | 0.00360132 | ADAM10/BCL2L1/EGFR/SLC7A11/ERBB2/TP73 |
| GO:0071248 | **cellular response to metal ion** | 6/108 | 188/18670 | 0.000787221 | 0.006154475 | 0.003694491 | CREB1/EGFR/HMOX1/CLIC4/TFAP2A/MAPK3 |
| GO:0045727 | **positive regulation of translation** | 5/108 | 127/18670 | 0.000857488 | 0.006542109 | 0.003927186 | RHOA/CDK4/ERBB2/MTOR/MAPK3 |
| GO:0048844 | **artery morphogenesis** | 4/108 | 76/18670 | 0.000986567 | 0.007317949 | 0.004392917 | SRF/AKT3/PRDM1/NOTCH3 |
| GO:0043276 | **anoikis** | 3/108 | 34/18670 | 0.000988628 | 0.007317949 | 0.004392917 | PDK4/MTOR/PTK2 |
| GO:0055007 | **cardiac muscle cell differentiation** | 5/108 | 132/18670 | 0.001019801 | 0.007516714 | 0.004512235 | SRF/MTOR/WT1/PPARA/GREM1 |
| GO:0050866 | **negative regulation of cell activation** | 6/108 | 199/18670 | 0.001056937 | 0.007773961 | 0.004666658 | AXL/HMOX1/SOCS1/ERBB2/SFRP1/NOS3 |
| GO:0050921 | **positive regulation of chemotaxis** | 5/108 | 135/18670 | 0.001127539 | 0.008189368 | 0.004916024 | ADAM10/RAC1/ADAM17/VEGFC/MAPK3 |
| GO:0051973 | **positive regulation of telomerase activity** | 3/108 | 36/18670 | 0.001169757 | 0.008416182 | 0.005052179 | GREM1/MYC/MAPK3 |
| GO:0030278 | **regulation of ossification** | 6/108 | 203/18670 | 0.001170863 | 0.008416182 | 0.005052179 | TFAP2A/SFRP1/GREM1/MAPK3/PTK2/TWIST1 |
| GO:0051193 | **regulation of cofactor metabolic process** | 3/108 | 37/18670 | 0.001267644 | 0.0088914 | 0.005337449 | PDK4/RAC1/SLC7A11 |
| GO:0072593 | **reactive oxygen species metabolic process** | 7/108 | 284/18670 | 0.001319726 | 0.009182657 | 0.005512289 | RHOA/EGFR/PDK4/RAC1/MTOR/TFAP2A/NOS3 |
| GO:0009612 | **response to mechanical stimulus** | 6/108 | 210/18670 | 0.001392641 | 0.009594059 | 0.005759251 | RHOA/ATP1A2/EGFR/RAC1/MAPK3/MMP7 |
| GO:0007220 | **Notch receptor processing** | 2/108 | 10/18670 | 0.001447431 | 0.00964674 | 0.005790875 | ADAM10/ADAM17 |
| GO:0045348 | **positive regulation of MHC class II biosynthetic process** | 2/108 | 10/18670 | 0.001447431 | 0.00964674 | 0.005790875 | XBP1/SIRT1 |
| GO:0097746 | **regulation of blood vessel diameter** | 5/108 | 143/18670 | 0.001456046 | 0.00964674 | 0.005790875 | RHOA/ATP1A2/EGFR/HMOX1/NOS3 |
| GO:0007162 | **negative regulation of cell adhesion** | 7/108 | 289/18670 | 0.001458519 | 0.00964674 | 0.005790875 | ADAM10/RHOA/WNT1/SOCS1/ERBB2/PPARA/PTK2 |
| GO:0070328 | **triglyceride homeostasis** | 3/108 | 39/18670 | 0.001478544 | 0.009687105 | 0.005815106 | XBP1/SIRT1/HNF4A |
| GO:0009798 | **axis specification** | 4/108 | 85/18670 | 0.001496326 | 0.009785182 | 0.005873981 | SRF/WNT1/WT1/SFRP1 |

Table S14: Result table of overrepresentation analysis for the group coronary heart disease. GeneRatio: involved targeted genes/all targeted genes; BgRatio: number of genes in that GOterm/total number of genes in all GOterms; geneID: involved genes.

| **ID** | **Description** | **GeneRatio** | **BgRatio** | **p-value** | **p-adjusted-value** | **q-value** | **geneID** |
| --- | --- | --- | --- | --- | --- | --- | --- |
| GO:0071902 | **positive regulation of protein serine/threonine kinase activity** | 13/74 | 334/18670 | 5.97736E-10 | 4.58314E-07 | 2.45701E-07 | ERBB2/TP73/NOD2/MAP3K8/GADD45A/HMGA2/AKT1/IFNG/MAP2K7/FZD4/DKK1/KRAS/SIRT1 |
| GO:0110110 | **positive regulation of animal organ morphogenesis** | 8/74 | 81/18670 | 1.05365E-09 | 6.46308E-07 | 3.46484E-07 | TFAP2A/WT1/MYC/CTNNB1/TGFBR2/DKK1/SOX9/TWIST1 |
| GO:0043491 | **protein kinase B signaling** | 11/74 | 269/18670 | 8.4844E-09 | 3.55593E-06 | 1.90633E-06 | ERBB2/MTOR/PIK3R3/LIN28A/AKT1/GAB1/MET/CCL2/SOX9/PTK2/SIRT1 |
| GO:0010001 | **glial cell differentiation** | 10/74 | 218/18670 | 1.43488E-08 | 3.87346E-06 | 2.07655E-06 | ERBB2/MTOR/TP73/CTNNB1/NF1/LIN28A/AKT1/IFNG/SOX9/KRAS |
| GO:0046620 | **regulation of organ growth** | 8/74 | 113/18670 | 1.51554E-08 | 3.87346E-06 | 2.07655E-06 | MTOR/TP73/WT1/TGFBR2/AKT1/FOXC1/MEIS1/PTK2 |
| GO:1903706 | **regulation of hemopoiesis** | 13/74 | 475/18670 | 4.04541E-08 | 7.68371E-06 | 4.11921E-06 | ERBB2/MTOR/TP73/MYC/RBFOX2/CTNNB1/NF1/TGFBR2/IFNG/ZEB1/RUNX3/FOXC1/MEIS1 |
| GO:2000679 | **positive regulation of transcription regulatory region DNA binding** | 5/74 | 25/18670 | 4.25898E-08 | 7.68371E-06 | 4.11921E-06 | CTNNB1/HMGA2/IFNG/FOXC1/TWIST1 |
| GO:0043010 | **camera-type eye development** | 11/74 | 319/18670 | 4.89208E-08 | 8.18083E-06 | 4.38572E-06 | TFAP2A/WT1/CTNNB1/NF1/TGFBR2/ZEB1/FZD4/FOXC1/MEIS1/SOX9/TWIST1 |
| GO:0072132 | **mesenchyme morphogenesis** | 6/74 | 52/18670 | 5.55729E-08 | 8.18083E-06 | 4.38572E-06 | MYC/TGFBR2/FOXC1/SOX9/NOS3/TWIST1 |
| GO:1901342 | **regulation of vasculature development** | 12/74 | 422/18670 | 9.41679E-08 | 1.11082E-05 | 5.95507E-06 | ERBB2/WT1/CTNNB1/NF1/TGFBR2/HMGA2/GAB1/RECK/FOXC1/NOS3/TWIST1/SIRT1 |
| GO:0008585 | **female gonad development** | 7/74 | 96/18670 | 1.03452E-07 | 1.17514E-05 | 6.29987E-06 | WT1/ANG/FZD4/FOXC1/HSPA5/NOS3/SIRT1 |
| GO:1901861 | **regulation of muscle tissue development** | 8/74 | 155/18670 | 1.78417E-07 | 1.43742E-05 | 7.70596E-06 | MTOR/TP73/NLN/CTNNB1/TGFBR2/DKK1/MEIS1/TWIST1 |
| GO:0010464 | **regulation of mesenchymal cell proliferation** | 5/74 | 33/18670 | 1.85618E-07 | 1.43742E-05 | 7.70596E-06 | MYC/CTNNB1/TGFBR2/ZEB1/SOX9 |
| GO:0030198 | **extracellular matrix organization** | 11/74 | 368/18670 | 2.06903E-07 | 1.5066E-05 | 8.07686E-06 | WT1/NF1/FGA/FGB/FGG/RECK/ATP7A/FOXC1/SOX9/MMP7/PTK2 |
| GO:0032872 | **regulation of stress-activated MAPK cascade** | 9/74 | 237/18670 | 3.96745E-07 | 2.38116E-05 | 1.27654E-05 | MYC/NOD2/GADD45A/FOXM1/FOXO1/AKT1/MAP2K7/FZD4/DKK1 |
| GO:0031998 | **regulation of fatty acid beta-oxidation** | 4/74 | 18/18670 | 6.67068E-07 | 3.40983E-05 | 1.828E-05 | MTOR/CPT1A/AKT1/TWIST1 |
| GO:0001933 | **negative regulation of protein phosphorylation** | 11/74 | 429/18670 | 9.43914E-07 | 4.34282E-05 | 2.32817E-05 | MTOR/MYC/GADD45A/FOXM1/FOXO1/NF1/AKT1/IFNG/DKK1/TWIST1/SIRT1 |
| GO:0007219 | **Notch signaling pathway** | 8/74 | 193/18670 | 9.48709E-07 | 4.34282E-05 | 2.32817E-05 | MYC/NOD2/TGFBR2/AKT1/FOXC1/SOX9/PTP4A3/NOS3 |
| GO:0062013 | **positive regulation of small molecule metabolic process** | 7/74 | 141/18670 | 1.42377E-06 | 5.52746E-05 | 2.96325E-05 | CPT1A/FOXO1/AKT1/IFNG/NOS3/TWIST1/SIRT1 |
| GO:0045834 | **positive regulation of lipid metabolic process** | 7/74 | 146/18670 | 1.79817E-06 | 6.72561E-05 | 3.60558E-05 | MTOR/NOD2/CPT1A/AKT1/IFNG/PTK2/TWIST1 |
| GO:0003179 | **heart valve morphogenesis** | 5/74 | 52/18670 | 1.91707E-06 | 6.86379E-05 | 3.67966E-05 | MTOR/TGFBR2/SOX9/NOS3/TWIST1 |
| GO:0035108 | **limb morphogenesis** | 7/74 | 148/18670 | 1.9694E-06 | 6.86379E-05 | 3.67966E-05 | TFAP2A/DLX5/CTNNB1/RECK/DKK1/SOX9/TWIST1 |
| GO:0051353 | **positive regulation of oxidoreductase activity** | 5/74 | 54/18670 | 2.31844E-06 | 7.56454E-05 | 4.05533E-05 | NOD2/AKT1/IFNG/ATP7A/KRAS |
| GO:0090342 | **regulation of cell aging** | 5/74 | 60/18670 | 3.93058E-06 | 0.000113727 | 6.09688E-05 | FOXM1/HMGA2/KRAS/TWIST1/SIRT1 |
| GO:2001233 | **regulation of apoptotic signaling pathway** | 10/74 | 406/18670 | 4.39952E-06 | 0.000121561 | 6.51687E-05 | TP73/ING5/CTNNB1/NF1/AKT1/FGA/FGB/FGG/NOS3/SIRT1 |
| GO:0030217 | **T cell differentiation** | 8/74 | 240/18670 | 4.8156E-06 | 0.000129557 | 6.9455E-05 | ERBB2/MTOR/CTNNB1/TGFBR2/IFNG/ZEB1/ATP7A/RUNX3 |
| GO:0009266 | **response to temperature stimulus** | 8/74 | 243/18670 | 5.27645E-06 | 0.000139508 | 7.47897E-05 | MTOR/FOXO1/NF1/AKT1/MAP2K7/HSPA5/NOS3/SIRT1 |
| GO:0060070 | **canonical Wnt signaling pathway** | 9/74 | 335/18670 | 6.86635E-06 | 0.00016239 | 8.70567E-05 | DLX5/CTNNB1/FOXO1/CTNND1/RECK/FRAT1/FZD4/DKK1/SOX9 |
| GO:0045792 | **negative regulation of cell size** | 3/74 | 10/18670 | 7.03112E-06 | 0.000163367 | 8.75806E-05 | MTOR/AKT1/RDX |
| GO:0071559 | **response to transforming growth factor beta** | 8/74 | 255/18670 | 7.51252E-06 | 0.000172044 | 9.22324E-05 | TGFBR2/ZEB1/NLK/RUNX3/HSPA5/SOX9/PTK2/SIRT1 |
| GO:0045907 | **positive regulation of vasoconstriction** | 4/74 | 32/18670 | 7.51676E-06 | 0.000172044 | 9.22324E-05 | AKT1/FGA/FGB/FGG |
| GO:0042698 | **ovulation cycle** | 5/74 | 69/18670 | 7.86727E-06 | 0.000174847 | 9.37352E-05 | FZD4/HSPA5/MMP7/NOS3/SIRT1 |
| GO:0050870 | **positive regulation of T cell activation** | 7/74 | 202/18670 | 1.52457E-05 | 0.000304688 | 0.000163343 | NOD2/MAP3K8/TGFBR2/AKT1/IFNG/RUNX3/CCL2 |
| GO:0001570 | **vasculogenesis** | 5/74 | 79/18670 | 1.5299E-05 | 0.000304688 | 0.000163343 | WT1/CTNNB1/TGFBR2/FZD4/PTK2 |
| GO:0002573 | **myeloid leukocyte differentiation** | 7/74 | 204/18670 | 1.62489E-05 | 0.000319457 | 0.00017126 | MTOR/MYC/CTNNB1/NF1/TGFBR2/IFNG/SIRT1 |
| GO:0014745 | **negative regulation of muscle adaptation** | 3/74 | 13/18670 | 1.66147E-05 | 0.000324568 | 0.000174 | MTOR/FOXO1/NOS3 |
| GO:0051216 | **cartilage development** | 7/74 | 209/18670 | 1.89979E-05 | 0.00035313 | 0.000189312 | CTNNB1/TGFBR2/HMGA2/ZEB1/ATP7A/RUNX3/SOX9 |
| GO:0071229 | **cellular response to acid chemical** | 7/74 | 209/18670 | 1.89979E-05 | 0.00035313 | 0.000189312 | MTOR/CPT1A/AKT1/ZEB1/FZD4/ATP7A/SOX9 |
| GO:0090287 | **regulation of cellular response to growth factor stimulus** | 8/74 | 292/18670 | 2.00456E-05 | 0.000370361 | 0.00019855 | CTNNB1/TGFBR2/ZEB1/FZD4/DKK1/HSPA5/PTP4A3/SIRT1 |
| GO:0001942 | **hair follicle development** | 5/74 | 86/18670 | 2.31375E-05 | 0.00040092 | 0.000214932 | CTNNB1/NF1/DKK1/ATP7A/SOX9 |
| GO:0071364 | **cellular response to epidermal growth factor stimulus** | 4/74 | 43/18670 | 2.49601E-05 | 0.000422943 | 0.000226739 | ERBB2/AKT1/FOXC1/SOX9 |
| GO:0035635 | **entry of bacterium into host cell** | 3/74 | 15/18670 | 2.62821E-05 | 0.000426399 | 0.000228591 | CTNNB1/CTNND1/MET |
| GO:0051770 | **positive regulation of nitric-oxide synthase biosynthetic process** | 3/74 | 15/18670 | 2.62821E-05 | 0.000426399 | 0.000228591 | NOD2/IFNG/CCL2 |
| GO:0043255 | **regulation of carbohydrate biosynthetic process** | 5/74 | 90/18670 | 2.88418E-05 | 0.000451315 | 0.000241949 | MTOR/NLN/FOXO1/AKT1/SIRT1 |
| GO:0030902 | **hindbrain development** | 6/74 | 152/18670 | 3.09669E-05 | 0.000479674 | 0.000257152 | RBFOX2/CTNNB1/FZD4/ATP7A/FOXC1/HSPA5 |
| GO:0048704 | **embryonic skeletal system morphogenesis** | 5/74 | 93/18670 | 3.37932E-05 | 0.000508058 | 0.000272368 | TFAP2A/CTNNB1/TGFBR2/ZEB1/TWIST1 |
| GO:0001894 | **tissue homeostasis** | 7/74 | 229/18670 | 3.41293E-05 | 0.000508128 | 0.000272406 | NOD2/CTNNB1/NF1/FOXC1/SOX9/KRAS/NOS3 |
| GO:0051961 | **negative regulation of nervous system development** | 8/74 | 316/18670 | 3.52213E-05 | 0.000514399 | 0.000275768 | TP73/CTNNB1/NF1/LIN28A/DKK1/MEIS1/SOX9/PTK2 |
| GO:1903320 | **regulation of protein modification by small protein conjugation or removal** | 7/74 | 231/18670 | 3.60732E-05 | 0.000521871 | 0.000279774 | MTOR/NOD2/CTNNB1/AKT1/BMI1/HSPA5/MTA1 |
| GO:0019827 | **stem cell population maintenance** | 6/74 | 157/18670 | 3.71418E-05 | 0.000534806 | 0.000286708 | IGF2BP1/CTNNB1/FOXO1/HMGA2/LIN28A/SOX9 |
| GO:0071453 | **cellular response to oxygen levels** | 7/74 | 234/18670 | 3.91593E-05 | 0.000556026 | 0.000298084 | MTOR/MYC/FOXO1/AKT1/ATP7A/TWIST1/SIRT1 |
| GO:2001022 | **positive regulation of response to DNA damage stimulus** | 5/74 | 97/18670 | 4.13927E-05 | 0.000574441 | 0.000307956 | MYC/FOXM1/MGMT/HMGA2/SIRT1 |
| GO:0007050 | **cell cycle arrest** | 7/74 | 237/18670 | 4.24592E-05 | 0.000583957 | 0.000313058 | MTOR/TP73/MYC/GADD45A/FOXM1/HMGA2/IFNG |
| GO:1900024 | **regulation of substrate adhesion-dependent cell spreading** | 4/74 | 52/18670 | 5.32995E-05 | 0.000683973 | 0.000366676 | FGA/FGB/FGG/PTK2 |
| GO:0048660 | **regulation of smooth muscle cell proliferation** | 6/74 | 169/18670 | 5.60475E-05 | 0.000698771 | 0.000374609 | MTOR/CTNNB1/TGFBR2/AKT1/ANG/IFNG |
| GO:0030195 | **negative regulation of blood coagulation** | 4/74 | 53/18670 | 5.74782E-05 | 0.000706254 | 0.000378621 | FGA/FGB/FGG/NOS3 |
| GO:0071496 | **cellular response to external stimulus** | 8/74 | 339/18670 | 5.78181E-05 | 0.000706254 | 0.000378621 | MTOR/GADD45A/FOXO1/AKT1/HSPA5/SOX9/MMP7/SIRT1 |
| GO:0045926 | **negative regulation of growth** | 7/74 | 249/18670 | 5.80293E-05 | 0.000706254 | 0.000378621 | TP73/WT1/ING5/TGFBR2/MEIS1/PTK2/SIRT1 |
| GO:0010721 | **negative regulation of cell development** | 8/74 | 344/18670 | 6.40644E-05 | 0.000755713 | 0.000405136 | TP73/CTNNB1/NF1/LIN28A/DKK1/MEIS1/SOX9/PTK2 |
| GO:1903202 | **negative regulation of oxidative stress-induced cell death** | 4/74 | 56/18670 | 7.14506E-05 | 0.000814643 | 0.000436728 | CTNNB1/AKT1/MET/SIRT1 |
| GO:0071347 | **cellular response to interleukin-1** | 6/74 | 179/18670 | 7.70942E-05 | 0.000847484 | 0.000454334 | NOD2/MAP3K8/FGB/FGG/CCL2/SOX9 |
| GO:0051147 | **regulation of muscle cell differentiation** | 6/74 | 181/18670 | 8.19747E-05 | 0.000876283 | 0.000469773 | MTOR/NLN/CTNNB1/ZEB1/DKK1/SIRT1 |
| GO:0010906 | **regulation of glucose metabolic process** | 5/74 | 112/18670 | 8.22854E-05 | 0.000876283 | 0.000469773 | MTOR/NLN/FOXO1/AKT1/SIRT1 |
| GO:0031331 | **positive regulation of cellular catabolic process** | 8/74 | 362/18670 | 9.14016E-05 | 0.000950267 | 0.000509435 | CPT1A/FOXO1/AKT1/IFNG/RDX/PTK2/TWIST1/SIRT1 |
| GO:0050714 | **positive regulation of protein secretion** | 7/74 | 268/18670 | 9.20285E-05 | 0.000953552 | 0.000511197 | NOD2/ANG/FGA/FGB/FGG/IFNG/TWIST1 |
| GO:0007565 | **female pregnancy** | 6/74 | 192/18670 | 0.000113407 | 0.001136663 | 0.000609362 | MTOR/TGFBR2/AKT1/RECK/KRAS/MMP7 |
| GO:0034332 | **adherens junction organization** | 4/74 | 65/18670 | 0.00012821 | 0.001245337 | 0.000667622 | CTNNB1/ANG/CTNND1/RDX |
| GO:0051258 | **protein polymerization** | 7/74 | 283/18670 | 0.000129081 | 0.001245337 | 0.000667622 | MTOR/ANG/FGA/FGB/FGG/MET/RDX |
| GO:0031639 | **plasminogen activation** | 3/74 | 25/18670 | 0.000129122 | 0.001245337 | 0.000667622 | FGA/FGB/FGG |
| GO:1904888 | **cranial skeletal system development** | 4/74 | 66/18670 | 0.000136074 | 0.001288086 | 0.000690539 | TFAP2A/CTNNB1/TGFBR2/TWIST1 |
| GO:1903959 | **regulation of anion transmembrane transport** | 3/74 | 26/18670 | 0.000145549 | 0.001336523 | 0.000716506 | MTOR/AKT1/ABCB1 |
| GO:0031667 | **response to nutrient levels** | 9/74 | 499/18670 | 0.000153111 | 0.001393449 | 0.000747024 | MTOR/NOD2/FOXO1/MGMT/TGFBR2/AKT1/HSPA5/MMP7/SIRT1 |
| GO:0030278 | **regulation of ossification** | 6/74 | 203/18670 | 0.000153747 | 0.001395093 | 0.000747906 | TFAP2A/CTNNB1/DKK1/SOX9/PTK2/TWIST1 |
| GO:0072378 | **blood coagulation, fibrin clot formation** | 3/74 | 27/18670 | 0.000163277 | 0.001459973 | 0.000782688 | FGA/FGB/FGG |
| GO:0018105 | **peptidyl-serine phosphorylation** | 7/74 | 299/18670 | 0.000181131 | 0.001593359 | 0.000854196 | MTOR/TGFBR2/HMGA2/AKT1/IFNG/NLK/DKK1 |
| GO:0045732 | **positive regulation of protein catabolic process** | 6/74 | 214/18670 | 0.000204702 | 0.001734309 | 0.000929758 | FOXO1/AKT1/IFNG/RDX/SOX9/PTK2 |
| GO:0008286 | **insulin receptor signaling pathway** | 5/74 | 141/18670 | 0.000242584 | 0.001957908 | 0.001049629 | PIK3R3/FOXO1/AKT1/GAB1/SIRT1 |
| GO:0038127 | **ERBB signaling pathway** | 5/74 | 142/18670 | 0.000250668 | 0.001991705 | 0.001067748 | ERBB2/AKT1/GAB1/SOX9/PTK2 |
| GO:0090398 | **cellular senescence** | 4/74 | 78/18670 | 0.00025983 | 0.002038103 | 0.001092621 | HMGA2/KRAS/TWIST1/SIRT1 |
| GO:0001649 | **osteoblast differentiation** | 6/74 | 225/18670 | 0.000268152 | 0.002087716 | 0.001119219 | DLX5/CTNNB1/NF1/AKT1/PTK2/TWIST1 |
| GO:0051090 | **regulation of DNA-binding transcription factor activity** | 8/74 | 432/18670 | 0.000305372 | 0.002284329 | 0.001224623 | NOD2/CTNNB1/AKT1/MXI1/FZD4/KRAS/TWIST1/SIRT1 |
| GO:0000302 | **response to reactive oxygen species** | 6/74 | 232/18670 | 0.000315969 | 0.002340767 | 0.001254879 | FOXO1/AKT1/MET/ATP7A/NOS3/SIRT1 |
| GO:0071695 | **anatomical structure maturation** | 6/74 | 232/18670 | 0.000315969 | 0.002340767 | 0.001254879 | MTOR/CTNNB1/NF1/ANG/FGG/RECK |
| GO:0010506 | **regulation of autophagy** | 7/74 | 328/18670 | 0.000318143 | 0.002351191 | 0.001260467 | MTOR/FOXO1/AKT1/IFNG/MET/PTK2/SIRT1 |
| GO:0031128 | **developmental induction** | 3/74 | 34/18670 | 0.000327447 | 0.002391146 | 0.001281887 | CTNNB1/DKK1/SOX9 |
| GO:0043276 | **anoikis** | 3/74 | 34/18670 | 0.000327447 | 0.002391146 | 0.001281887 | MTOR/AKT1/PTK2 |
| GO:0048145 | **regulation of fibroblast proliferation** | 4/74 | 83/18670 | 0.000329772 | 0.002402399 | 0.00128792 | MYC/CTNNB1/NF1/BMI1 |
| GO:0051973 | **positive regulation of telomerase activity** | 3/74 | 36/18670 | 0.000388489 | 0.002751722 | 0.001475191 | MYC/CTNNB1/MAP2K7 |
| GO:0043271 | **negative regulation of ion transport** | 5/74 | 157/18670 | 0.000398269 | 0.002808022 | 0.001505373 | MTOR/AKT1/ATP7A/NOS3/TWIST1 |
| GO:0045921 | **positive regulation of exocytosis** | 4/74 | 88/18670 | 0.000412274 | 0.002886862 | 0.001547639 | FGA/FGB/FGG/IFNG |
| GO:0006476 | **protein deacetylation** | 4/74 | 93/18670 | 0.000508564 | 0.003435605 | 0.001841819 | IFNG/MTA3/MTA1/SIRT1 |
| GO:1901655 | **cellular response to ketone** | 4/74 | 93/18670 | 0.000508564 | 0.003435605 | 0.001841819 | GNAI1/FOXO1/AKT1/SIRT1 |
| GO:0110149 | **regulation of biomineralization** | 4/74 | 94/18670 | 0.000529585 | 0.00355413 | 0.00190536 | TFAP2A/SOX9/NOS3/TWIST1 |
| GO:0048863 | **stem cell differentiation** | 6/74 | 257/18670 | 0.000543663 | 0.003632708 | 0.001947485 | TP73/HMGA2/LIN28A/FOXC1/SOX9/TWIST1 |
| GO:0032231 | **regulation of actin filament bundle assembly** | 4/74 | 97/18670 | 0.000596357 | 0.003908174 | 0.002095162 | MTOR/MET/RDX/RHOC |
| GO:0007281 | **germ cell development** | 6/74 | 262/18670 | 0.000601562 | 0.003933882 | 0.002108944 | MTOR/WT1/CTNNB1/LIN28A/AKT1/ANG |
| GO:0043087 | **regulation of GTPase activity** | 8/74 | 479/18670 | 0.000605482 | 0.003942703 | 0.002113673 | ERBB2/MTOR/NF1/RDX/RSU1/CCL2/TBC1D9/PTK2 |
| GO:0031641 | **regulation of myelination** | 3/74 | 42/18670 | 0.000614071 | 0.003981723 | 0.002134591 | MTOR/CTNNB1/AKT1 |
| GO:0016049 | **cell growth** | 8/74 | 484/18670 | 0.00064804 | 0.004158031 | 0.00222911 | ERBB2/MTOR/WT1/CTNNB1/TGFBR2/AKT1/SOX9/SIRT1 |
| GO:0045429 | **positive regulation of nitric oxide biosynthetic process** | 3/74 | 43/18670 | 0.000658254 | 0.00421475 | 0.002259516 | MTOR/AKT1/IFNG |
| GO:0060923 | **cardiac muscle cell fate commitment** | 2/74 | 10/18670 | 0.000683228 | 0.004302793 | 0.002306716 | WT1/DKK1 |
| GO:0071281 | **cellular response to iron ion** | 2/74 | 10/18670 | 0.000683228 | 0.004302793 | 0.002306716 | TFAP2A/ATP7A |
| GO:2000109 | **regulation of macrophage apoptotic process** | 2/74 | 10/18670 | 0.000683228 | 0.004302793 | 0.002306716 | NOD2/SIRT1 |
| GO:0038084 | **vascular endothelial growth factor signaling pathway** | 3/74 | 44/18670 | 0.000704416 | 0.004391146 | 0.002354082 | GAB1/FOXC1/PTP4A3 |
| GO:0048771 | **tissue remodeling** | 5/74 | 179/18670 | 0.000723033 | 0.004488951 | 0.002406515 | CTNNB1/NF1/ATP7A/FOXC1/NOS3 |
| GO:0035987 | **endodermal cell differentiation** | 3/74 | 45/18670 | 0.000752592 | 0.004543699 | 0.002435865 | CTNNB1/HMGA2/DKK1 |
| GO:0021953 | **central nervous system neuron differentiation** | 5/74 | 183/18670 | 0.000798682 | 0.004765676 | 0.002554866 | DLX5/CTNNB1/DKK1/ATP7A/PTK2 |
| GO:0060856 | **establishment of blood-brain barrier** | 2/74 | 11/18670 | 0.000832914 | 0.004865805 | 0.002608545 | CTNNB1/RECK |
| GO:1905941 | **positive regulation of gonad development** | 2/74 | 11/18670 | 0.000832914 | 0.004865805 | 0.002608545 | WT1/SOX9 |
| GO:2000767 | **positive regulation of cytoplasmic translation** | 2/74 | 11/18670 | 0.000832914 | 0.004865805 | 0.002608545 | MTOR/LIN28A |
| GO:0008542 | **visual learning** | 3/74 | 48/18670 | 0.000909547 | 0.005214168 | 0.002795302 | MTOR/NF1/KRAS |
| GO:0045446 | **endothelial cell differentiation** | 4/74 | 114/18670 | 0.001091808 | 0.006110536 | 0.003275842 | CTNNB1/MET/RDX/ZEB1 |
| GO:0090068 | **positive regulation of cell cycle process** | 6/74 | 298/18670 | 0.001172889 | 0.006494156 | 0.0034815 | TP73/GADD45A/HMGA2/AKT1/RDX/MTA3 |
| GO:0003306 | **Wnt signaling pathway involved in heart development** | 2/74 | 13/18670 | 0.001175173 | 0.006494156 | 0.0034815 | CTNNB1/DKK1 |
| GO:1902947 | **regulation of tau-protein kinase activity** | 2/74 | 13/18670 | 0.001175173 | 0.006494156 | 0.0034815 | IFNG/DKK1 |
| GO:0072089 | **stem cell proliferation** | 4/74 | 120/18670 | 0.001320277 | 0.007192345 | 0.003855797 | CTNNB1/NF1/HMGA2/ABCB1 |
| GO:0043551 | **regulation of phosphatidylinositol 3-kinase activity** | 3/74 | 55/18670 | 0.001352506 | 0.007306948 | 0.003917235 | PIK3R3/NOD2/PTK2 |
| GO:0035020 | **regulation of Rac protein signal transduction** | 2/74 | 14/18670 | 0.001367521 | 0.007306948 | 0.003917235 | NF1/KRAS |
| GO:0035358 | **regulation of peroxisome proliferator activated receptor signaling pathway** | 2/74 | 14/18670 | 0.001367521 | 0.007306948 | 0.003917235 | TWIST1/SIRT1 |
| GO:1905477 | **positive regulation of protein localization to membrane** | 4/74 | 122/18670 | 0.001403328 | 0.00744628 | 0.003991931 | ERBB2/TP73/AKT1/IFNG |
| GO:0043414 | **macromolecule methylation** | 6/74 | 309/18670 | 0.001411475 | 0.00744628 | 0.003991931 | WT1/MYC/CTNNB1/MGMT/SIRT1/FBXO11 |
| GO:0090276 | **regulation of peptide hormone secretion** | 5/74 | 208/18670 | 0.001413021 | 0.00744628 | 0.003991931 | CPT1A/FGA/FGB/FGG/IFNG |
| GO:0009612 | **response to mechanical stimulus** | 5/74 | 210/18670 | 0.001473932 | 0.007727436 | 0.004142658 | GADD45A/TGFBR2/AKT1/SOX9/MMP7 |
| GO:0043523 | **regulation of neuron apoptotic process** | 5/74 | 210/18670 | 0.001473932 | 0.007727436 | 0.004142658 | TFAP2A/CTNNB1/NF1/CCL2/KRAS |
| GO:0035067 | **negative regulation of histone acetylation** | 2/74 | 15/18670 | 0.001573866 | 0.008037565 | 0.004308917 | TWIST1/SIRT1 |
| GO:0043518 | **negative regulation of DNA damage response, signal transduction by p53 class mediator** | 2/74 | 15/18670 | 0.001573866 | 0.008037565 | 0.004308917 | TWIST1/SIRT1 |
| GO:2000402 | **negative regulation of lymphocyte migration** | 2/74 | 15/18670 | 0.001573866 | 0.008037565 | 0.004308917 | AKT1/CCL2 |
| GO:0070527 | **platelet aggregation** | 3/74 | 59/18670 | 0.001657065 | 0.008372682 | 0.004488573 | FGA/FGB/FGG |
| GO:0032886 | **regulation of microtubule-based process** | 5/74 | 218/18670 | 0.001737104 | 0.008702403 | 0.004665335 | ERBB2/GNAI1/CTNNB1/MET/STAG2 |
| GO:0090335 | **regulation of brown fat cell differentiation** | 2/74 | 16/18670 | 0.001794096 | 0.008877604 | 0.00475926 | MTOR/SIRT1 |
| GO:1900451 | **positive regulation of glutamate receptor signaling pathway** | 2/74 | 16/18670 | 0.001794096 | 0.008877604 | 0.00475926 | IFNG/CCL2 |
| GO:0002287 | **alpha-beta T cell activation involved in immune response** | 3/74 | 61/18670 | 0.001824139 | 0.008965758 | 0.004806519 | MTOR/IFNG/ATP7A |
| GO:0034113 | **heterotypic cell-cell adhesion** | 3/74 | 61/18670 | 0.001824139 | 0.008965758 | 0.004806519 | FGA/FGB/FGG |
| GO:0007292 | **female gamete generation** | 4/74 | 136/18670 | 0.002089516 | 0.009940994 | 0.00532934 | CTNNB1/ANG/NOS3/SIRT1 |

Table S15: Result table of overrepresentation analysis for the group carotid stenosis. GeneRatio: involved targeted genes/all targeted genes; BgRatio: number of genes in that GOterm/total number of genes in all GOterms; geneID: involved genes.

| **ID** | **Description** | **GeneRatio** | **BgRatio** | **p-value** | **p-adjusted-value** | **q-value** | **geneID** |
| --- | --- | --- | --- | --- | --- | --- | --- |
| GO:0016202 | **regulation of striated muscle tissue development** | 10/62 | 152/18670 | 7.10314E-11 | 4.52106E-08 | 2.33598E-08 | MTOR/TP73/NLN/PPARA/GREM1/GJA1/TWIST1/HDAC4/DKK1/MEIS1 |
| GO:0046620 | **regulation of organ growth** | 9/62 | 113/18670 | 1.22393E-10 | 5.16148E-08 | 2.66687E-08 | MTOR/TP73/WT1/PPARA/GJA1/AKT1/FOXC1/MEIS1/PTK2 |
| GO:0008585 | **female gonad development** | 8/62 | 96/18670 | 9.77529E-10 | 2.2994E-07 | 1.18807E-07 | WT1/SFRP1/ESR1/NOS3/FZD4/FOXC1/HSPA5/SIRT1 |
| GO:0042698 | **ovulation cycle** | 7/62 | 69/18670 | 2.88495E-09 | 4.24422E-07 | 2.19294E-07 | EGFR/ESR1/NOS3/FZD4/HSPA5/MMP7/SIRT1 |
| GO:0071364 | **cellular response to epidermal growth factor stimulus** | 6/62 | 43/18670 | 5.79715E-09 | 7.13049E-07 | 3.68424E-07 | ERBB2/EGFR/ID1/AKT1/FOXC1/SOX9 |
| GO:0045926 | **negative regulation of growth** | 10/62 | 249/18670 | 8.6983E-09 | 1.02709E-06 | 5.30688E-07 | TP73/WT1/ING5/PPARA/SFRP1/GJA1/MEIS1/PTK2/SIRT1/CDKN1A |
| GO:0043491 | **protein kinase B signaling** | 10/62 | 269/18670 | 1.81454E-08 | 1.67391E-06 | 8.6489E-07 | ERBB2/MTOR/PIK3R3/EGFR/ESR1/AKT1/CCL2/SOX9/PTK2/SIRT1 |
| GO:0043010 | **camera-type eye development** | 10/62 | 319/18670 | 9.02179E-08 | 6.04667E-06 | 3.12424E-06 | TFAP2A/WT1/EGFR/PAX6/GJA1/TWIST1/FZD4/FOXC1/MEIS1/SOX9 |
| GO:0009266 | **response to temperature stimulus** | 9/62 | 243/18670 | 1.02882E-07 | 6.46185E-06 | 3.33876E-06 | MTOR/NOS3/NFKBIA/ANO1/MAP2K7/AKT1/HSPA5/SIRT1/CDKN1A |
| GO:0071902 | **positive regulation of protein serine/threonine kinase activity** | 10/62 | 334/18670 | 1.38355E-07 | 8.33516E-06 | 4.30668E-06 | ERBB2/TP73/EGFR/MAP2K7/FZD4/DKK1/AKT1/HMGA2/KRAS/SIRT1 |
| GO:0051052 | **regulation of DNA metabolic process** | 10/62 | 351/18670 | 2.19076E-07 | 1.21769E-05 | 6.29164E-06 | WT1/EGFR/GREM1/GJA1/TWIST1/MAP2K7/AKT1/HMGA2/SIRT1/CDKN1A |
| GO:0031998 | **regulation of fatty acid beta-oxidation** | 4/62 | 18/18670 | 3.25748E-07 | 1.52636E-05 | 7.88653E-06 | MTOR/PPARA/TWIST1/AKT1 |
| GO:0060485 | **mesenchyme development** | 9/62 | 279/18670 | 3.31573E-07 | 1.52938E-05 | 7.90212E-06 | MTOR/WT1/SFRP1/GREM1/NOS3/TWIST1/FOXC1/SOX9/HMGA2 |
| GO:0042063 | **gliogenesis** | 9/62 | 290/18670 | 4.58769E-07 | 1.96273E-05 | 1.01412E-05 | ERBB2/MTOR/TP73/EGFR/PAX6/AKT1/CCL2/SOX9/KRAS |
| GO:0110149 | **regulation of biomineralization** | 6/62 | 94/18670 | 6.78999E-07 | 2.63738E-05 | 1.3627E-05 | TFAP2A/GREM1/NOS3/GJA1/TWIST1/SOX9 |
| GO:0045668 | **negative regulation of osteoblast differentiation** | 5/62 | 51/18670 | 7.15624E-07 | 2.74353E-05 | 1.41755E-05 | SFRP1/GREM1/ID1/TWIST1/HDAC4 |
| GO:1901214 | **regulation of neuron death** | 9/62 | 313/18670 | 8.67415E-07 | 3.24128E-05 | 1.67473E-05 | MTOR/TFAP2A/PPARA/HDAC4/DKK1/AKT1/CCL2/KRAS/SIRT1 |
| GO:0001558 | **regulation of cell growth** | 10/62 | 416/18670 | 1.03514E-06 | 3.81966E-05 | 1.97357E-05 | ERBB2/MTOR/WT1/EGFR/PPARA/SFRP1/GJA1/AKT1/SIRT1/CDKN1A |
| GO:2000679 | **positive regulation of transcription regulatory region DNA binding** | 4/62 | 25/18670 | 1.3234E-06 | 4.5961E-05 | 2.37475E-05 | PAX6/TWIST1/FOXC1/HMGA2 |
| GO:0001933 | **negative regulation of protein phosphorylation** | 10/62 | 429/18670 | 1.3665E-06 | 4.69058E-05 | 2.42357E-05 | MTOR/PEBP1/SFRP1/GREM1/PAX6/TWIST1/DKK1/AKT1/SIRT1/CDKN1A |
| GO:0048863 | **stem cell differentiation** | 8/62 | 257/18670 | 2.057E-06 | 6.67282E-05 | 3.44777E-05 | TP73/SFRP1/GREM1/ESR1/TWIST1/FOXC1/SOX9/HMGA2 |
| GO:0051147 | **regulation of muscle cell differentiation** | 7/62 | 181/18670 | 2.24809E-06 | 6.91286E-05 | 3.57179E-05 | MTOR/NLN/PPARA/GREM1/HDAC4/DKK1/SIRT1 |
| GO:0009612 | **response to mechanical stimulus** | 7/62 | 210/18670 | 6.00027E-06 | 0.000151497 | 7.82765E-05 | EGFR/GJA1/NFKBIA/HDAC4/AKT1/SOX9/MMP7 |
| GO:0060348 | **bone development** | 7/62 | 217/18670 | 7.43752E-06 | 0.000182963 | 9.45348E-05 | TFAP2A/GREM1/GJA1/TWIST1/FOXC1/MEIS1/SOX9 |
| GO:0048145 | **regulation of fibroblast proliferation** | 5/62 | 83/18670 | 8.15368E-06 | 0.000197292 | 0.000101939 | EGFR/SFRP1/ESR1/BMI1/CDKN1A |
| GO:0045665 | **negative regulation of neuron differentiation** | 7/62 | 225/18670 | 9.41964E-06 | 0.000218951 | 0.000113129 | TP73/PAX6/ID1/DKK1/MEIS1/SOX9/PTK2 |
| GO:1901342 | **regulation of vasculature development** | 9/62 | 422/18670 | 9.93585E-06 | 0.00022562 | 0.000116575 | ERBB2/WT1/SFRP1/NOS3/ID1/TWIST1/FOXC1/HMGA2/SIRT1 |
| GO:0061614 | **pri-miRNA transcription by RNA polymerase II** | 4/62 | 47/18670 | 1.76675E-05 | 0.000366639 | 0.000189438 | WT1/PPARA/HDAC4/SOX9 |
| GO:0046850 | **regulation of bone remodeling** | 4/62 | 48/18670 | 1.92259E-05 | 0.000391413 | 0.000202238 | EGFR/SFRP1/GREM1/GJA1 |
| GO:2000379 | **positive regulation of reactive oxygen species metabolic process** | 5/62 | 102/18670 | 2.22879E-05 | 0.00044157 | 0.000228154 | MTOR/EGFR/HDAC4/AKT1/CDKN1A |
| GO:0050999 | **regulation of nitric-oxide synthase activity** | 4/62 | 51/18670 | 2.45087E-05 | 0.000460827 | 0.000238104 | EGFR/NOS3/AKT1/KRAS |
| GO:0018108 | **peptidyl-tyrosine phosphorylation** | 8/62 | 363/18670 | 2.55884E-05 | 0.000469174 | 0.000242417 | ERBB2/MTOR/EGFR/SFRP1/GREM1/WEE1/MAP2K7/PTK2 |
| GO:0003179 | **heart valve morphogenesis** | 4/62 | 52/18670 | 2.64853E-05 | 0.000473846 | 0.000244831 | MTOR/NOS3/TWIST1/SOX9 |
| GO:2000772 | **regulation of cellular senescence** | 4/62 | 52/18670 | 2.64853E-05 | 0.000473846 | 0.000244831 | TWIST1/HMGA2/KRAS/SIRT1 |
| GO:0043502 | **regulation of muscle adaptation** | 5/62 | 107/18670 | 2.80879E-05 | 0.0004885 | 0.000252402 | MTOR/PPARA/NOS3/HDAC4/PTK2 |
| GO:0010975 | **regulation of neuron projection development** | 9/62 | 499/18670 | 3.73023E-05 | 0.000601147 | 0.000310605 | MTOR/SFRP1/SNAP25/ID1/FZD4/DKK1/AKT1/HSPA5/PTK2 |
| GO:0032233 | **positive regulation of actin filament bundle assembly** | 4/62 | 61/18670 | 4.99263E-05 | 0.000740615 | 0.000382667 | MTOR/SFRP1/ID1/RHOC |
| GO:0090287 | **regulation of cellular response to growth factor stimulus** | 7/62 | 292/18670 | 5.02003E-05 | 0.000740957 | 0.000382844 | SFRP1/GREM1/FZD4/DKK1/HSPA5/PTP4A3/SIRT1 |
| GO:0090101 | **negative regulation of transmembrane receptor protein serine/threonine kinase signaling pathway** | 5/62 | 126/18670 | 6.14867E-05 | 0.000876853 | 0.00045306 | SFRP1/GREM1/DKK1/HSPA5/SIRT1 |
| GO:0071456 | **cellular response to hypoxia** | 6/62 | 207/18670 | 6.33382E-05 | 0.000894614 | 0.000462236 | MTOR/SFRP1/TWIST1/AKT1/ATP7A/SIRT1 |
| GO:0007254 | **JNK cascade** | 6/62 | 214/18670 | 7.61311E-05 | 0.001040778 | 0.000537758 | EGFR/SFRP1/MAP2K7/FZD4/DKK1/AKT1 |
| GO:0055007 | **cardiac muscle cell differentiation** | 5/62 | 132/18670 | 7.66985E-05 | 0.001043383 | 0.000539103 | MTOR/WT1/PPARA/GREM1/DKK1 |
| GO:1903959 | **regulation of anion transmembrane transport** | 3/62 | 26/18670 | 8.58632E-05 | 0.001126525 | 0.000582062 | MTOR/AKT1/ABCB1 |
| GO:0048520 | **positive regulation of behavior** | 3/62 | 28/18670 | 0.000107677 | 0.001305138 | 0.000674349 | MTOR/GJA1/HDAC4 |
| GO:0038127 | **ERBB signaling pathway** | 5/62 | 142/18670 | 0.000108319 | 0.001305138 | 0.000674349 | ERBB2/EGFR/AKT1/SOX9/PTK2 |
| GO:0097746 | **regulation of blood vessel diameter** | 5/62 | 143/18670 | 0.00011196 | 0.001332679 | 0.00068858 | EGFR/NOS3/GJA1/AKT1/FOXC1 |
| GO:1903320 | **regulation of protein modification by small protein conjugation or removal** | 6/62 | 231/18670 | 0.000115894 | 0.001368479 | 0.000707077 | MTOR/HDAC4/AKT1/BMI1/HSPA5/MTA1 |
| GO:0048873 | **homeostasis of number of cells within a tissue** | 3/62 | 30/18670 | 0.000132815 | 0.001502198 | 0.000776168 | NOS3/SOX9/KRAS |
| GO:0031669 | **cellular response to nutrient levels** | 6/62 | 237/18670 | 0.000133325 | 0.001502198 | 0.000776168 | MTOR/SFRP1/TXN2/HSPA5/SIRT1/CDKN1A |
| GO:0042110 | **T cell activation** | 8/62 | 464/18670 | 0.000142822 | 0.00157907 | 0.000815886 | ERBB2/MTOR/CD1C/GJA1/AKT1/ATP7A/RUNX3/CCL2 |
| GO:0003401 | **axis elongation** | 3/62 | 31/18670 | 0.000146698 | 0.001609863 | 0.000831797 | SFRP1/ESR1/SOX9 |
| GO:0030902 | **hindbrain development** | 5/62 | 152/18670 | 0.000149114 | 0.001630312 | 0.000842363 | RBFOX2/FZD4/ATP7A/FOXC1/HSPA5 |
| GO:0019318 | **hexose metabolic process** | 6/62 | 244/18670 | 0.000156226 | 0.001683137 | 0.000869657 | MTOR/NLN/PPARA/FUT4/AKT1/SIRT1 |
| GO:0043271 | **negative regulation of ion transport** | 5/62 | 157/18670 | 0.000173468 | 0.001822338 | 0.00094158 | MTOR/NOS3/TWIST1/AKT1/ATP7A |
| GO:2000241 | **regulation of reproductive process** | 5/62 | 160/18670 | 0.000189472 | 0.001962531 | 0.001014016 | WT1/SFRP1/ESR1/HDAC4/SOX9 |
| GO:0001942 | **hair follicle development** | 4/62 | 86/18670 | 0.000190957 | 0.001970997 | 0.001018391 | EGFR/DKK1/ATP7A/SOX9 |
| GO:0043276 | **anoikis** | 3/62 | 34/18670 | 0.000193912 | 0.0019876 | 0.001026969 | MTOR/AKT1/PTK2 |
| GO:0030198 | **extracellular matrix organization** | 7/62 | 368/18670 | 0.000211453 | 0.002110186 | 0.001090308 | WT1/GREM1/ATP7A/FOXC1/SOX9/MMP7/PTK2 |
| GO:0045185 | **maintenance of protein location** | 4/62 | 90/18670 | 0.000227507 | 0.002231229 | 0.001152849 | MXI1/NFKBIA/AKT1/HSPA5 |
| GO:0034614 | **cellular response to reactive oxygen species** | 5/62 | 168/18670 | 0.000237683 | 0.002308031 | 0.001192532 | EGFR/NOS3/AKT1/ATP7A/SIRT1 |
| GO:0048660 | **regulation of smooth muscle cell proliferation** | 5/62 | 169/18670 | 0.00024431 | 0.002349193 | 0.0012138 | MTOR/EGFR/HDAC4/AKT1/CDKN1A |
| GO:0034405 | **response to fluid shear stress** | 3/62 | 37/18670 | 0.000250008 | 0.002360432 | 0.001219607 | NOS3/GJA1/AKT1 |
| GO:0045927 | **positive regulation of growth** | 6/62 | 270/18670 | 0.000269906 | 0.002467256 | 0.001274802 | ERBB2/MTOR/WT1/EGFR/SFRP1/AKT1 |
| GO:1990830 | **cellular response to leukemia inhibitory factor** | 4/62 | 96/18670 | 0.000291418 | 0.00263078 | 0.001359293 | PAX6/FZD4/PTP4A3/SIRT1 |
| GO:0001657 | **ureteric bud development** | 4/62 | 97/18670 | 0.000303201 | 0.002704073 | 0.001397162 | WT1/SFRP1/FOXC1/SOX9 |
| GO:1903034 | **regulation of response to wounding** | 5/62 | 179/18670 | 0.000318585 | 0.002807354 | 0.001450526 | MTOR/NOS3/GJA1/ATP7A/CDKN1A |
| GO:0022408 | **negative regulation of cell-cell adhesion** | 5/62 | 180/18670 | 0.000326858 | 0.002845956 | 0.001470472 | ERBB2/PPARA/AKT1/RUNX3/PTK2 |
| GO:0021953 | **central nervous system neuron differentiation** | 5/62 | 183/18670 | 0.000352658 | 0.003009589 | 0.001555019 | SFRP1/PAX6/DKK1/ATP7A/PTK2 |
| GO:2000045 | **regulation of G1/S transition of mitotic cell cycle** | 5/62 | 184/18670 | 0.000361591 | 0.003067289 | 0.001584832 | EGFR/WEE1/AKT1/CCL2/CDKN1A |
| GO:0019233 | **sensory perception of pain** | 4/62 | 104/18670 | 0.000395464 | 0.003251839 | 0.001680186 | MTOR/ANO1/CCL2/SMR3B |
| GO:0098732 | **macromolecule deacylation** | 4/62 | 104/18670 | 0.000395464 | 0.003251839 | 0.001680186 | HDAC4/MTA3/MTA1/SIRT1 |
| GO:0071356 | **cellular response to tumor necrosis factor** | 6/62 | 291/18670 | 0.000402479 | 0.003282091 | 0.001695817 | SFRP1/NFKBIA/HDAC4/AKT1/CCL2/SIRT1 |
| GO:0003300 | **cardiac muscle hypertrophy** | 4/62 | 106/18670 | 0.000425141 | 0.003419659 | 0.001766897 | MTOR/PPARA/HDAC4/PTK2 |
| GO:0042771 | **intrinsic apoptotic signaling pathway in response to DNA damage by p53 class mediator** | 3/62 | 45/18670 | 0.000448029 | 0.003536314 | 0.001827171 | TP73/SIRT1/CDKN1A |
| GO:0032526 | **response to retinoic acid** | 4/62 | 108/18670 | 0.000456375 | 0.003564828 | 0.001841904 | GJA1/FZD4/DKK1/SOX9 |
| GO:0090068 | **positive regulation of cell cycle process** | 6/62 | 298/18670 | 0.000456472 | 0.003564828 | 0.001841904 | TP73/EGFR/AKT1/HMGA2/MTA3/CDKN1A |
| GO:0010883 | **regulation of lipid storage** | 3/62 | 46/18670 | 0.000478156 | 0.003661234 | 0.001891716 | PPARA/NFKBIA/SIRT1 |
| GO:0045792 | **negative regulation of cell size** | 2/62 | 10/18670 | 0.000479979 | 0.003661234 | 0.001891716 | MTOR/AKT1 |
| GO:0009896 | **positive regulation of catabolic process** | 7/62 | 425/18670 | 0.000503979 | 0.003812361 | 0.001969801 | PPARA/GJA1/TWIST1/AKT1/SOX9/PTK2/SIRT1 |
| GO:0046879 | **hormone secretion** | 6/62 | 308/18670 | 0.000543246 | 0.004049653 | 0.002092407 | EGFR/SFRP1/SNAP25/GJA1/ANO1/FZD4 |
| GO:0002686 | **negative regulation of leukocyte migration** | 3/62 | 49/18670 | 0.000576243 | 0.004251292 | 0.002196592 | GREM1/AKT1/CCL2 |
| GO:0070555 | **response to interleukin-1** | 5/62 | 207/18670 | 0.000618509 | 0.00441024 | 0.002278718 | SFRP1/NFKBIA/HDAC4/CCL2/SOX9 |
| GO:0016051 | **carbohydrate biosynthetic process** | 5/62 | 208/18670 | 0.000632141 | 0.004464308 | 0.002306655 | MTOR/NLN/PPARA/AKT1/SIRT1 |
| GO:0051216 | **cartilage development** | 5/62 | 209/18670 | 0.000645997 | 0.004551276 | 0.00235159 | GREM1/ATP7A/RUNX3/SOX9/HMGA2 |
| GO:0071107 | **response to parathyroid hormone** | 2/62 | 12/18670 | 0.000700961 | 0.004789902 | 0.002474885 | GJA1/HDAC4 |
| GO:1905477 | **positive regulation of protein localization to membrane** | 4/62 | 122/18670 | 0.000722546 | 0.00490335 | 0.002533502 | ERBB2/TP73/EGFR/AKT1 |
| GO:1904951 | **positive regulation of establishment of protein localization** | 7/62 | 456/18670 | 0.000764091 | 0.005132125 | 0.002651708 | ERBB2/TP73/EGFR/GJA1/TWIST1/ANO1/PTP4A3 |
| GO:0045620 | **negative regulation of lymphocyte differentiation** | 3/62 | 55/18670 | 0.000809011 | 0.005366741 | 0.002772931 | ERBB2/SFRP1/RUNX3 |
| GO:0006338 | **chromatin remodeling** | 5/62 | 220/18670 | 0.00081391 | 0.005386832 | 0.002783312 | ESR1/HDAC4/SOX9/HMGA2/SIRT1 |
| GO:0010745 | **negative regulation of macrophage derived foam cell differentiation** | 2/62 | 13/18670 | 0.000826638 | 0.005386832 | 0.002783312 | PPARA/NFKBIA |
| GO:0045820 | **negative regulation of glycolytic process** | 2/62 | 13/18670 | 0.000826638 | 0.005386832 | 0.002783312 | PPARA/HDAC4 |
| GO:0060688 | **regulation of morphogenesis of a branching structure** | 3/62 | 56/18670 | 0.00085279 | 0.005508613 | 0.002846234 | SFRP1/ESR1/SOX9 |
| GO:0045930 | **negative regulation of mitotic cell cycle** | 6/62 | 338/18670 | 0.000882423 | 0.005662851 | 0.002925927 | EGFR/WEE1/FOXC1/CCL2/HMGA2/CDKN1A |
| GO:0071391 | **cellular response to estrogen stimulus** | 2/62 | 14/18670 | 0.00096235 | 0.006018763 | 0.003109823 | SFRP1/ESR1 |
| GO:0045598 | **regulation of fat cell differentiation** | 4/62 | 132/18670 | 0.00096972 | 0.006026553 | 0.003113848 | MTOR/SFRP1/AKT1/SIRT1 |
| GO:0000098 | **sulfur amino acid catabolic process** | 2/62 | 15/18670 | 0.001108031 | 0.006581301 | 0.00340048 | TXN2/MAT1A |
| GO:0033689 | **negative regulation of osteoblast proliferation** | 2/62 | 15/18670 | 0.001108031 | 0.006581301 | 0.00340048 | SFRP1/GREM1 |
| GO:0035067 | **negative regulation of histone acetylation** | 2/62 | 15/18670 | 0.001108031 | 0.006581301 | 0.00340048 | TWIST1/SIRT1 |
| GO:0043518 | **negative regulation of DNA damage response, signal transduction by p53 class mediator** | 2/62 | 15/18670 | 0.001108031 | 0.006581301 | 0.00340048 | TWIST1/SIRT1 |
| GO:2000095 | **regulation of Wnt signaling pathway, planar cell polarity pathway** | 2/62 | 15/18670 | 0.001108031 | 0.006581301 | 0.00340048 | SFRP1/DKK1 |
| GO:0032922 | **circadian regulation of gene expression** | 3/62 | 62/18670 | 0.001147168 | 0.006745894 | 0.003485523 | PPARA/MTA1/SIRT1 |
| GO:0051402 | **neuron apoptotic process** | 5/62 | 239/18670 | 0.001178072 | 0.006900138 | 0.003565219 | TFAP2A/HDAC4/HSPA5/CCL2/KRAS |
| GO:0022412 | **cellular process involved in reproduction in multicellular organism** | 6/62 | 361/18670 | 0.001238386 | 0.007168068 | 0.003703655 | MTOR/WT1/SFRP1/FZD4/AKT1/FOXC1 |
| GO:0010875 | **positive regulation of cholesterol efflux** | 2/62 | 16/18670 | 0.001263615 | 0.007243093 | 0.00374242 | NFKBIA/SIRT1 |
| GO:0071380 | **cellular response to prostaglandin E stimulus** | 2/62 | 16/18670 | 0.001263615 | 0.007243093 | 0.00374242 | SFRP1/AKT1 |
| GO:0000086 | **G2/M transition of mitotic cell cycle** | 5/62 | 247/18670 | 0.001362951 | 0.007737367 | 0.003997805 | WEE1/ABCB1/HMGA2/MTA3/CDKN1A |
| GO:0007612 | **learning** | 4/62 | 145/18670 | 0.001373069 | 0.00776494 | 0.004012052 | MTOR/SNAP25/DKK1/KRAS |
| GO:0097501 | **stress response to metal ion** | 2/62 | 17/18670 | 0.001429039 | 0.008004787 | 0.004135978 | ATP7A/HSPA5 |
| GO:0035924 | **cellular response to vascular endothelial growth factor stimulus** | 3/62 | 68/18670 | 0.001498797 | 0.008269998 | 0.004273009 | AKT1/FOXC1/PTP4A3 |
| GO:0001756 | **somitogenesis** | 3/62 | 70/18670 | 0.001629393 | 0.008810249 | 0.00455215 | SFRP1/DKK1/FOXC1 |
| GO:0042133 | **neurotransmitter metabolic process** | 4/62 | 153/18670 | 0.001672531 | 0.008993284 | 0.004646723 | MTOR/NOS3/AKT1/ATP7A |
| GO:0060193 | **positive regulation of lipase activity** | 3/62 | 72/18670 | 0.001766908 | 0.009397769 | 0.004855715 | EGFR/ESR1/RHOC |
| GO:0032095 | **regulation of response to food** | 2/62 | 19/18670 | 0.001789142 | 0.009397769 | 0.004855715 | MTOR/PPARA |
| GO:1902176 | **negative regulation of oxidative stress-induced intrinsic apoptotic signaling pathway** | 2/62 | 19/18670 | 0.001789142 | 0.009397769 | 0.004855715 | AKT1/SIRT1 |
| GO:1903798 | **regulation of production of miRNAs involved in gene silencing by miRNA** | 2/62 | 19/18670 | 0.001789142 | 0.009397769 | 0.004855715 | EGFR/ESR1 |
| GO:0001764 | **neuron migration** | 4/62 | 157/18670 | 0.001838089 | 0.00950379 | 0.004910495 | PAX6/GJA1/TWIST1/PTK2 |
| GO:0019827 | **stem cell population maintenance** | 4/62 | 157/18670 | 0.001838089 | 0.00950379 | 0.004910495 | IGF2BP1/SFRP1/SOX9/HMGA2 |
| GO:0010959 | **regulation of metal ion transport** | 6/62 | 394/18670 | 0.001930194 | 0.009892245 | 0.005111205 | NOS3/GJA1/P2RX5/AKT1/ATP7A/CCL2 |
| GO:0022898 | **regulation of transmembrane transporter activity** | 5/62 | 268/18670 | 0.001949943 | 0.009976136 | 0.00515455 | GJA1/TWIST1/ATP7A/ABCB1/CCL2 |
| GO:0010042 | **response to manganese ion** | 2/62 | 20/18670 | 0.001983692 | 0.009989711 | 0.005161564 | ATP7A/HSPA5 |
| GO:0060216 | **definitive hemopoiesis** | 2/62 | 20/18670 | 0.001983692 | 0.009989711 | 0.005161564 | RBFOX2/MEIS1 |

Table S16: Result table of overrepresentation analysis for the group peripheral artery disease. GeneRatio: involved targeted genes/all targeted genes; BgRatio: number of genes in that GOterm/total number of genes in all GOterms; geneID: involved genes.

| **ID** | **Description** | **GeneRatio** | **BgRatio** | **p-value** | **p-adjusted-value** | **q-value** | **geneID** |
| --- | --- | --- | --- | --- | --- | --- | --- |
| GO:0030099 | **myeloid cell differentiation** | 21/102 | 416/18670 | 7.61303E-15 | 1.3909E-11 | 7.74125E-12 | MTOR/ACVR1B/JAG1/CDKN1C/CCR7/ETS1/HIF1A/JUNB/LIF/PIK3CD/VEGFA/MAFB/PIAS3/SIRT1/RUNX1/RB1/CTNNBIP1/MYC/IRF4/RBFOX2/SFRP1 |
| GO:0110110 | **positive regulation of animal organ morphogenesis** | 12/102 | 81/18670 | 1.88162E-14 | 1.71886E-11 | 9.56655E-12 | TFAP2A/WT1/JAG1/EDN1/LIF/SMAD4/TGFB2/TGFBR1/VEGFA/WNT2/MYC/XBP1 |
| GO:0071560 | **cellular response to transforming growth factor beta stimulus** | 16/102 | 249/18670 | 4.22741E-13 | 1.0298E-10 | 5.73148E-11 | ACVR1B/CAV1/CDKN1C/EDN1/HSPA5/ITGA3/SMAD3/SMAD4/TGFB2/TGFBR1/WNT2/SIRT1/ACVR2B/SFRP1/DKK3/TRIM33 |
| GO:0001558 | **regulation of cell growth** | 19/102 | 416/18670 | 9.98248E-13 | 1.84959E-10 | 1.02941E-10 | ERBB2/MTOR/WT1/ACVR1B/APOE/DDR1/EDN1/GSK3B/SMAD3/SMAD4/SMARCA2/TGFB2/TGFBR1/VEGFA/RAB21/SIRT1/RB1/SFRP1/HNF4A |
| GO:1903706 | **regulation of hemopoiesis** | 20/102 | 475/18670 | 1.05205E-12 | 1.84959E-10 | 1.02941E-10 | ERBB2/MTOR/TP73/ACVR1B/JAG1/ETS1/HIF1A/LIF/PSMD9/MAFB/PIAS3/RUNX1/RB1/CTNNBIP1/MYC/PRDM1/IRF4/XBP1/RBFOX2/SFRP1 |
| GO:0030111 | **regulation of Wnt signaling pathway** | 18/102 | 363/18670 | 1.06298E-12 | 1.84959E-10 | 1.02941E-10 | APOE/CAV1/CDH2/GSK3B/ITGA3/SMAD3/NFKB1/PSMD9/FZD6/WNK1/XIAP/RUNX1/ZEB2/CTNNBIP1/DLX5/SFRP1/AXIN2/DKK3 |
| GO:0071902 | **positive regulation of protein serine/threonine kinase activity** | 16/102 | 334/18670 | 3.63119E-11 | 5.10322E-09 | 2.84027E-09 | ERBB2/TP73/EDN1/ERN1/EZH2/KRAS/MAP3K11/TGFBR1/VEGFA/FZD4/MAP4K3/SIRT1/SLC27A1/ZEB2/NOD2/CCND1 |
| GO:0001666 | **response to hypoxia** | 16/102 | 359/18670 | 1.05942E-10 | 1.13856E-08 | 6.33684E-09 | MTOR/CAV1/EDN1/ETS1/HIF1A/HK2/SMAD3/SMAD4/PSMD9/PTGS2/TGFB2/VEGFA/BECN1/SIRT1/MYC/SFRP1 |
| GO:0001654 | **eye development** | 16/102 | 362/18670 | 1.19784E-10 | 1.25055E-08 | 6.9601E-09 | TFAP2A/WT1/JAG1/CDKN1C/HIF1A/SMAD3/NECTIN1/TGFB2/TGFBR1/VEGFA/WNT2/FZD4/ATF6/ACVR2B/ZEB2/PRDM1 |
| GO:0051403 | **stress-activated MAPK cascade** | 14/102 | 286/18670 | 5.07838E-10 | 4.41819E-08 | 2.45901E-08 | CCR7/EDN1/ERN1/IKBKB/MAP3K11/NFKB1/TGFB2/VEGFA/FZD4/MAP4K3/ZEB2/MYC/NOD2/SFRP1 |
| GO:2001233 | **regulation of apoptotic signaling pathway** | 16/102 | 406/18670 | 6.41759E-10 | 5.45346E-08 | 3.0352E-08 | TP73/ING5/CAV1/CD44/CTSC/GSK3B/HIF1A/SMAD3/PTGS2/SNAI1/TGFBR1/BECN1/SIRT1/RB1/XBP1/SFRP1 |
| GO:0001763 | **morphogenesis of a branching structure** | 12/102 | 196/18670 | 7.56859E-10 | 6.1457E-08 | 3.42047E-08 | WT1/DDR1/EDN1/KRAS/SMAD4/VEGFA/WNT2/SETD2/CTNNBIP1/MYC/PRDM1/SFRP1 |
| GO:0007595 | **lactation** | 7/102 | 48/18670 | 7.22615E-09 | 4.55247E-07 | 2.53374E-07 | DDR1/CAV1/HIF1A/HK2/VEGFA/XBP1/CCND1 |
| GO:0048872 | **homeostasis of number of cells** | 12/102 | 246/18670 | 9.92299E-09 | 5.94403E-07 | 3.30823E-07 | ACVR1B/CDH2/CCR7/ETS1/EZH2/HIF1A/KRAS/PIK3CD/VEGFA/MAFB/RB1/RBFOX2 |
| GO:0045926 | **negative regulation of growth** | 12/102 | 249/18670 | 1.13611E-08 | 6.58942E-07 | 3.66744E-07 | TP73/WT1/ING5/ACVR1B/HIF1A/SMAD3/SMAD4/SMARCA2/TGFB2/SIRT1/SFRP1/HNF4A |
| GO:0051090 | **regulation of DNA-binding transcription factor activity** | 15/102 | 432/18670 | 1.27218E-08 | 7.26337E-07 | 4.04253E-07 | CAV1/EDN1/EZH2/IKBKB/KRAS/SMAD3/NFKB1/VEGFA/WNT2/FZD4/FZD6/SIRT1/RB1/CTNNBIP1/NOD2 |
| GO:0048863 | **stem cell differentiation** | 12/102 | 257/18670 | 1.61562E-08 | 8.81113E-07 | 4.90396E-07 | TP73/JAG1/EDN1/HIF1A/LIF/SMAD4/PSMD9/TGFB2/SETD2/RUNX1/ZEB2/SFRP1 |
| GO:0007548 | **sex differentiation** | 12/102 | 270/18670 | 2.79068E-08 | 1.41627E-06 | 7.88246E-07 | WT1/ACVR1B/HSPA5/SMAD4/TGFB2/TGFBR1/VEGFA/FZD4/SIRT1/CCND1/SFRP1/HNF4A |
| GO:0003170 | **heart valve development** | 7/102 | 61/18670 | 4.04055E-08 | 1.89284E-06 | 1.05349E-06 | MTOR/JAG1/SMAD4/SNAI1/TGFB2/RB1/PRDM1 |
| GO:0060840 | **artery development** | 8/102 | 100/18670 | 7.38839E-08 | 3.1392E-06 | 1.74717E-06 | JAG1/APOE/LDLR/TGFB2/TGFBR1/VEGFA/ACVR2B/PRDM1 |
| GO:0018105 | **peptidyl-serine phosphorylation** | 12/102 | 299/18670 | 8.54012E-08 | 3.46729E-06 | 1.92977E-06 | MTOR/CAV1/CD44/ERN1/GSK3B/IKBKB/LIF/PDE4D/PTGS2/TGFBR1/VEGFA/WNK1 |
| GO:0007565 | **female pregnancy** | 10/102 | 192/18670 | 9.82017E-08 | 3.81733E-06 | 2.12459E-06 | MTOR/ACVR1B/DDR1/ETS1/ITGA3/JUNB/KRAS/LIF/PTGS2/PRDM1 |
| GO:1901522 | **positive regulation of transcription from RNA polymerase II promoter involved in cellular response to chemical stimulus** | 5/102 | 22/18670 | 1.07842E-07 | 4.10473E-06 | 2.28455E-06 | HIF1A/SMAD4/VEGFA/DLX5/XBP1 |
| GO:1901861 | **regulation of muscle tissue development** | 9/102 | 155/18670 | 1.76039E-07 | 6.12615E-06 | 3.40959E-06 | MTOR/TP73/NLN/EDN1/ERBB3/SMAD3/SMAD4/TGFBR1/WNT2 |
| GO:0061614 | **pri-miRNA transcription by RNA polymerase II** | 6/102 | 47/18670 | 2.05277E-07 | 6.88149E-06 | 3.82999E-06 | WT1/ETS1/HIF1A/SMAD3/SMAD4/TGFB2 |
| GO:0001933 | **negative regulation of protein phosphorylation** | 13/102 | 429/18670 | 6.07915E-07 | 1.70918E-05 | 9.51267E-06 | MTOR/APOE/CAV1/CDKN1C/LIF/SMAD4/PDE4D/SIRT1/WNK1/RB1/PTPRT/MYC/SFRP1 |
| GO:0071453 | **cellular response to oxygen levels** | 10/102 | 234/18670 | 6.13794E-07 | 1.71206E-05 | 9.52873E-06 | MTOR/CAV1/EDN1/HIF1A/PSMD9/PTGS2/VEGFA/SIRT1/MYC/SFRP1 |
| GO:0045598 | **regulation of fat cell differentiation** | 8/102 | 132/18670 | 6.33422E-07 | 1.74024E-05 | 9.68557E-06 | MTOR/JAG1/SMAD3/PTGS2/SULT1E1/SIRT1/XBP1/SFRP1 |
| GO:0007050 | **cell cycle arrest** | 10/102 | 237/18670 | 6.89558E-07 | 1.85268E-05 | 1.03113E-05 | MTOR/TP73/CDKN1C/ERN1/SMAD3/TGFB2/TGFBR1/RB1/MYC/CCND1 |
| GO:0007369 | **gastrulation** | 9/102 | 185/18670 | 7.8619E-07 | 2.05196E-05 | 1.14204E-05 | ITGA3/SMAD3/SMAD4/SNAI1/SETD2/WNK1/ACVR2B/SFRP1/HNF4A |
| GO:0048545 | **response to steroid hormone** | 12/102 | 383/18670 | 1.20073E-06 | 2.96451E-05 | 1.64994E-05 | CAV1/EDN1/KRAS/PTGS2/TGFB2/SIRT1/RUNX1/RB1/RBFOX2/CCND1/SFRP1/HNF4A |
| GO:0007568 | **aging** | 11/102 | 321/18670 | 1.44916E-06 | 3.35656E-05 | 1.86814E-05 | MTOR/SRR/CDKN1C/CTSC/EDN1/KRAS/PDE4D/PTGS2/BECN1/KL/SIRT1 |
| GO:0071634 | **regulation of transforming growth factor beta production** | 5/102 | 36/18670 | 1.45299E-06 | 3.35656E-05 | 1.86814E-05 | HIF1A/SMAD3/SMAD4/PTGS2/TGFB2 |
| GO:0071542 | **dopaminergic neuron differentiation** | 5/102 | 37/18670 | 1.67277E-06 | 3.74987E-05 | 2.08704E-05 | GSK3B/HIF1A/VEGFA/WNT2/SFRP1 |
| GO:1905898 | **positive regulation of response to endoplasmic reticulum stress** | 5/102 | 38/18670 | 1.9179E-06 | 4.22168E-05 | 2.34964E-05 | CAV1/ERN1/ATF6/SIRT1/XBP1 |
| GO:0051341 | **regulation of oxidoreductase activity** | 7/102 | 107/18670 | 1.96755E-06 | 4.29872E-05 | 2.39251E-05 | APOE/CAV1/EDN1/HIF1A/KRAS/NFKB1/NOD2 |
| GO:0048638 | **regulation of developmental growth** | 11/102 | 347/18670 | 3.07651E-06 | 6.17139E-05 | 3.43477E-05 | MTOR/TP73/WT1/APOE/EDN1/GSK3B/TGFBR1/VEGFA/WNT2/RAB21/SFRP1 |
| GO:1901342 | **regulation of vasculature development** | 12/102 | 422/18670 | 3.27874E-06 | 6.44114E-05 | 3.5849E-05 | ERBB2/WT1/ETS1/HIF1A/HK2/PTGS2/TGFB2/VEGFA/SIRT1/RUNX1/XBP1/SFRP1 |
| GO:0097193 | **intrinsic apoptotic signaling pathway** | 10/102 | 289/18670 | 4.108E-06 | 7.81803E-05 | 4.35123E-05 | TP73/CAV1/CD44/ERN1/HIF1A/PTGS2/SNAI1/BECN1/SIRT1/XBP1 |
| GO:0042063 | **gliogenesis** | 10/102 | 290/18670 | 4.2356E-06 | 8.01912E-05 | 4.46315E-05 | ERBB2/MTOR/TP73/CDH2/ERBB3/EZH2/KRAS/LDLR/LIF/TGFB2 |
| GO:0001570 | **vasculogenesis** | 6/102 | 79/18670 | 4.61511E-06 | 8.56021E-05 | 4.7643E-05 | WT1/CAV1/JUNB/VEGFA/FZD4/SETD2 |
| GO:0048588 | **developmental cell growth** | 9/102 | 234/18670 | 5.45099E-06 | 9.90942E-05 | 5.51523E-05 | MTOR/APOE/DDR1/EDN1/GSK3B/VEGFA/RAB21/ALCAM/ZEB2 |
| GO:0051098 | **regulation of binding** | 11/102 | 373/18670 | 6.12759E-06 | 0.00010869 | 6.04931E-05 | APOE/CAV1/GSK3B/HSPA5/LIF/SMAD3/SMAD4/TGFBR1/RB1/CTNNBIP1/IRF4 |
| GO:0050727 | **regulation of inflammatory response** | 11/102 | 374/18670 | 6.28492E-06 | 0.000110942 | 6.17465E-05 | APOE/CTSC/CCR7/ETS1/LDLR/SMAD3/NFKB1/PTGS2/XIAP/RB1/NOD2 |
| GO:0030217 | **T cell differentiation** | 9/102 | 240/18670 | 6.69015E-06 | 0.00011531 | 6.41776E-05 | ERBB2/MTOR/CCR7/PIK3CD/MAFB/RUNX1/PRDM1/IRF4/XBP1 |
| GO:0051147 | **regulation of muscle cell differentiation** | 8/102 | 181/18670 | 6.7452E-06 | 0.000115713 | 6.44019E-05 | MTOR/NLN/CDH2/EDN1/EZH2/SMAD4/SIRT1/XBP1 |
| GO:0031346 | **positive regulation of cell projection organization** | 11/102 | 383/18670 | 7.86734E-06 | 0.000133089 | 7.40726E-05 | MTOR/APOE/CCR7/EZH2/HSPA5/ITGA3/TGFBR1/VEGFA/FZD4/RAB21/ZEB2 |
| GO:0071248 | **cellular response to metal ion** | 8/102 | 188/18670 | 8.90818E-06 | 0.000145966 | 8.12396E-05 | TFAP2A/CDH1/EDN1/HSPA5/JUNB/PTGS2/BECN1/WNK1 |
| GO:0007409 | **axonogenesis** | 12/102 | 468/18670 | 9.37182E-06 | 0.000152198 | 8.47081E-05 | ERBB2/APOE/CDH2/GSK3B/SMAD4/PIK3CD/NECTIN1/VEGFA/RAB21/ALCAM/ZEB2/DLX5 |
| GO:1900101 | **regulation of endoplasmic reticulum unfolded protein response** | 4/102 | 25/18670 | 9.72464E-06 | 0.000156537 | 8.71227E-05 | ERN1/HSPA5/ATF6/XBP1 |
| GO:0043255 | **regulation of carbohydrate biosynthetic process** | 6/102 | 90/18670 | 9.84889E-06 | 0.000157152 | 8.74652E-05 | MTOR/NLN/CLTC/GSK3B/NFKB1/SIRT1 |
| GO:0050769 | **positive regulation of neurogenesis** | 12/102 | 474/18670 | 1.06482E-05 | 0.000168435 | 9.37446E-05 | MTOR/TP73/APOE/EZH2/HIF1A/HSPA5/ITGA3/LIF/VEGFA/FZD4/RAB21/ZEB2 |
| GO:1901654 | **response to ketone** | 8/102 | 193/18670 | 1.07891E-05 | 0.000169928 | 9.45758E-05 | CAV1/CCR7/EDN1/TGFB2/SIRT1/GNAI1/CCND1/SFRP1 |
| GO:0042632 | **cholesterol homeostasis** | 6/102 | 96/18670 | 1.42826E-05 | 0.000217452 | 0.000121026 | APOE/CAV1/LDLR/SIRT1/XBP1/HNF4A |
| GO:0035265 | **organ growth** | 8/102 | 204/18670 | 1.61313E-05 | 0.000236722 | 0.000131751 | MTOR/TP73/WT1/EDN1/TGFB2/TGFBR1/WNT2/ACVR2B |
| GO:0045927 | **positive regulation of growth** | 9/102 | 270/18670 | 1.7166E-05 | 0.000246947 | 0.000137442 | ERBB2/MTOR/WT1/EDN1/TGFB2/TGFBR1/VEGFA/WNT2/SFRP1 |
| GO:0061351 | **neural precursor cell proliferation** | 7/102 | 150/18670 | 1.83278E-05 | 0.00025659 | 0.000142809 | IGF2BP1/CDH2/HIF1A/VEGFA/WNT2/FZD6/ZEB2 |
| GO:0043966 | **histone H3 acetylation** | 5/102 | 60/18670 | 1.89742E-05 | 0.000263619 | 0.000146721 | ING5/LIF/SMAD4/SIRT1/IRF4 |
| GO:0071229 | **cellular response to acid chemical** | 8/102 | 209/18670 | 1.92106E-05 | 0.000265892 | 0.000147986 | MTOR/EDN1/LDLR/VEGFA/WNT2/FZD4/XBP1/SFRP1 |
| GO:0051348 | **negative regulation of transferase activity** | 9/102 | 285/18670 | 2.62933E-05 | 0.000343127 | 0.000190972 | APOE/CAV1/CDKN1C/GSK3B/SIRT1/WNK1/RB1/PTPRT/SFRP1 |
| GO:0045428 | **regulation of nitric oxide biosynthetic process** | 5/102 | 66/18670 | 3.0254E-05 | 0.000379353 | 0.000211134 | MTOR/CAV1/EDN1/SMAD3/PTGS2 |
| GO:1904888 | **cranial skeletal system development** | 5/102 | 66/18670 | 3.0254E-05 | 0.000379353 | 0.000211134 | TFAP2A/SMAD3/TGFB2/TGFBR1/SETD2 |
| GO:0010464 | **regulation of mesenchymal cell proliferation** | 4/102 | 33/18670 | 3.04189E-05 | 0.000379353 | 0.000211134 | VEGFA/WNT2/CTNNBIP1/MYC |
| GO:0042698 | **ovulation cycle** | 5/102 | 69/18670 | 3.75559E-05 | 0.00044555 | 0.000247977 | ETS1/HSPA5/TGFB2/FZD4/SIRT1 |
| GO:0001756 | **somitogenesis** | 5/102 | 70/18670 | 4.02706E-05 | 0.000462732 | 0.00025754 | SMAD3/SMAD4/ZEB2/SFRP1/AXIN2 |
| GO:0060560 | **developmental growth involved in morphogenesis** | 8/102 | 235/18670 | 4.43589E-05 | 0.00049568 | 0.000275877 | APOE/DDR1/GSK3B/VEGFA/RAB21/ALCAM/ZEB2/SFRP1 |
| GO:0001819 | **positive regulation of cytokine production** | 11/102 | 464/18670 | 4.62343E-05 | 0.00051194 | 0.000284927 | CCR7/HIF1A/SMAD3/NFKB1/PDE4D/PTGS2/SETD2/RUNX1/NOD2/IRF4/XBP1 |
| GO:0046879 | **hormone secretion** | 9/102 | 308/18670 | 4.81402E-05 | 0.000528187 | 0.00029397 | EDN1/HIF1A/LIF/SMAD4/PSMD9/FZD4/ACVR2B/SFRP1/HNF4A |
| GO:1901653 | **cellular response to peptide** | 10/102 | 385/18670 | 4.88403E-05 | 0.000532724 | 0.000296495 | PIK3R3/CAV1/EDN1/GSK3B/NFKB1/KL/SIRT1/NOD2/SMARCC1/XBP1 |
| GO:1903524 | **positive regulation of blood circulation** | 5/102 | 73/18670 | 4.93344E-05 | 0.00053492 | 0.000297717 | CAV1/EDN1/PDE4D/PTGS2/TGFB2 |
| GO:0048469 | **cell maturation** | 7/102 | 177/18670 | 5.29308E-05 | 0.000563876 | 0.000313833 | MTOR/CDKN1C/HIF1A/VEGFA/RND1/RB1/XBP1 |
| GO:0010611 | **regulation of cardiac muscle hypertrophy** | 5/102 | 75/18670 | 5.62015E-05 | 0.000593527 | 0.000330335 | MTOR/EDN1/SMAD3/SMAD4/BECN1 |
| GO:0071347 | **cellular response to interleukin-1** | 7/102 | 179/18670 | 5.68329E-05 | 0.000598465 | 0.000333084 | EDN1/HIF1A/IKBKB/NFKB1/PSMD9/NOD2/SFRP1 |
| GO:0030501 | **positive regulation of bone mineralization** | 4/102 | 39/18670 | 5.96249E-05 | 0.000617193 | 0.000343508 | TFAP2A/SMAD3/KL/ACVR2B |
| GO:0045667 | **regulation of osteoblast differentiation** | 6/102 | 126/18670 | 6.65059E-05 | 0.000669456 | 0.000372595 | JAG1/SMAD3/ACVR2B/CTNNBIP1/SFRP1/AXIN2 |
| GO:0043518 | **negative regulation of DNA damage response, signal transduction by p53 class mediator** | 3/102 | 15/18670 | 6.86791E-05 | 0.000678253 | 0.000377491 | CD44/SNAI1/SIRT1 |
| GO:0055089 | **fatty acid homeostasis** | 3/102 | 15/18670 | 6.86791E-05 | 0.000678253 | 0.000377491 | APOE/SIRT1/XBP1 |
| GO:1903053 | **regulation of extracellular matrix organization** | 4/102 | 41/18670 | 7.27996E-05 | 0.000707472 | 0.000393753 | DDR1/ETS1/SMAD3/RB1 |
| GO:0007043 | **cell-cell junction assembly** | 6/102 | 129/18670 | 7.5804E-05 | 0.000732772 | 0.000407834 | CAV1/CDH1/CDH2/IKBKB/SNAI1/RUNX1 |
| GO:0010506 | **regulation of autophagy** | 9/102 | 328/18670 | 7.80838E-05 | 0.000752819 | 0.000418992 | MTOR/ERN1/GSK3B/HIF1A/HK2/BECN1/SIRT1/DRAM1/XBP1 |
| GO:0003158 | **endothelium development** | 6/102 | 132/18670 | 8.61109E-05 | 0.000807684 | 0.000449528 | JAG1/IKBKB/SMAD4/PDE4D/VEGFA/ACVR2B |
| GO:2000106 | **regulation of leukocyte apoptotic process** | 5/102 | 83/18670 | 9.13374E-05 | 0.000836458 | 0.000465542 | CCR7/HIF1A/PIK3CD/SIRT1/NOD2 |
| GO:0007159 | **leukocyte cell-cell adhesion** | 9/102 | 337/18670 | 9.59645E-05 | 0.000873773 | 0.000486311 | ERBB2/CAV1/CD44/CCR7/ETS1/WNK1/RUNX1/NOD2/XBP1 |
| GO:1902931 | **negative regulation of alcohol biosynthetic process** | 3/102 | 17/18670 | 0.000101829 | 0.000913966 | 0.00050868 | APOE/NFKB1/DKK3 |
| GO:0001942 | **hair follicle development** | 5/102 | 86/18670 | 0.00010815 | 0.000959173 | 0.000533841 | ACVR1B/SMAD4/SNAI1/TGFB2/FZD6 |
| GO:0070542 | **response to fatty acid** | 5/102 | 86/18670 | 0.00010815 | 0.000959173 | 0.000533841 | CCR7/EDN1/LDLR/PTGS2/SFRP1 |
| GO:1903725 | **regulation of phospholipid metabolic process** | 5/102 | 88/18670 | 0.000120612 | 0.001032126 | 0.000574444 | PIK3R3/CCR7/LDLR/SLC27A1/NOD2 |
| GO:0050852 | **T cell receptor signaling pathway** | 7/102 | 202/18670 | 0.00012125 | 0.001035157 | 0.000576131 | CCR7/IKBKB/NFKB1/PDE4D/PIK3CD/PSMD9/WNK1 |
| GO:0060977 | **coronary vasculature morphogenesis** | 3/102 | 18/18670 | 0.00012171 | 0.001036662 | 0.000576969 | TGFBR1/VEGFA/SETD2 |
| GO:0010862 | **positive regulation of pathway-restricted SMAD protein phosphorylation** | 4/102 | 48/18670 | 0.000135838 | 0.001135813 | 0.000632152 | ACVR1B/SMAD4/TGFB2/TGFBR1 |
| GO:0051216 | **cartilage development** | 7/102 | 209/18670 | 0.000149728 | 0.00123222 | 0.000685809 | CD44/EDN1/HIF1A/SMAD3/SNAI1/TGFBR1/AXIN2 |
| GO:0043523 | **regulation of neuron apoptotic process** | 7/102 | 210/18670 | 0.000154205 | 0.001266216 | 0.00070473 | TFAP2A/APOE/ERBB3/HIF1A/KRAS/MAP3K11/TGFB2 |
| GO:0061041 | **regulation of wound healing** | 6/102 | 148/18670 | 0.000161501 | 0.001312328 | 0.000730395 | MTOR/APOE/CAV1/EDN1/SMAD3/XBP1 |
| GO:0016579 | **protein deubiquitination** | 8/102 | 283/18670 | 0.000161617 | 0.001312328 | 0.000730395 | HIF1A/SMAD3/SMAD4/PSMD9/TGFBR1/BECN1/KDM1B/MYC |
| GO:0009267 | **cellular response to starvation** | 6/102 | 149/18670 | 0.000167541 | 0.001339593 | 0.000745569 | MTOR/HSPA5/BECN1/SIRT1/XBP1/SFRP1 |
| GO:0060039 | **pericardium development** | 3/102 | 20/18670 | 0.000168691 | 0.00134291 | 0.000747415 | WT1/SMAD3/SETD2 |
| GO:0051496 | **positive regulation of stress fiber assembly** | 4/102 | 51/18670 | 0.000172281 | 0.001359643 | 0.000756728 | MTOR/SMAD3/TGFBR1/SFRP1 |
| GO:0030301 | **cholesterol transport** | 5/102 | 99/18670 | 0.000210011 | 0.001608761 | 0.000895378 | APOE/CAV1/LDLR/NFKB1/SIRT1 |
| GO:0019827 | **stem cell population maintenance** | 6/102 | 157/18670 | 0.000222562 | 0.001694252 | 0.000942959 | IGF2BP1/JAG1/CDH2/LIF/SMAD4/SFRP1 |
| GO:0044321 | **response to leptin** | 3/102 | 22/18670 | 0.000226078 | 0.001706797 | 0.000949942 | EDN1/SIRT1/CCND1 |
| GO:1903320 | **regulation of protein modification by small protein conjugation or removal** | 7/102 | 231/18670 | 0.000276334 | 0.002011406 | 0.001119476 | MTOR/CAV1/HSPA5/TGFBR1/PIAS3/XIAP/NOD2 |
| GO:0009755 | **hormone-mediated signaling pathway** | 7/102 | 233/18670 | 0.000291164 | 0.002102598 | 0.00117023 | SIRT1/SLC27A1/RUNX1/RB1/RBFOX2/SFRP1/HNF4A |
| GO:0050863 | **regulation of T cell activation** | 8/102 | 314/18670 | 0.000326449 | 0.002320713 | 0.001291625 | ERBB2/CAV1/CCR7/RUNX1/NOD2/PRDM1/IRF4/XBP1 |
| GO:0051048 | **negative regulation of secretion** | 7/102 | 238/18670 | 0.000331055 | 0.002335281 | 0.001299733 | APOE/EDN1/ERBB3/LIF/PSMD9/WNK1/SFRP1 |
| GO:0051894 | **positive regulation of focal adhesion assembly** | 3/102 | 25/18670 | 0.000333654 | 0.002340063 | 0.001302394 | SMAD3/VEGFA/SFRP1 |
| GO:1990778 | **protein localization to cell periphery** | 8/102 | 319/18670 | 0.000362766 | 0.002514884 | 0.001399693 | CAV1/CDH1/CDH2/CLTC/IKBKB/ITGA3/LIN7A/GNAI1 |
| GO:0009266 | **response to temperature stimulus** | 7/102 | 243/18670 | 0.000375221 | 0.002561084 | 0.001425407 | MTOR/GSK3B/HSPA5/PTGS2/SIRT1/DNAJA4/SLC27A1 |
| GO:0010921 | **regulation of phosphatase activity** | 6/102 | 175/18670 | 0.000398473 | 0.002681438 | 0.001492392 | MTOR/GSK3B/IKBKB/SMAD3/TGFB2/WNK1 |
| GO:0090316 | **positive regulation of intracellular protein transport** | 6/102 | 176/18670 | 0.000410735 | 0.002743738 | 0.001527065 | ERBB2/TP73/CDH1/GSK3B/SMAD3/PTGS2 |
| GO:0043550 | **regulation of lipid kinase activity** | 4/102 | 64/18670 | 0.000414869 | 0.002751235 | 0.001531238 | PIK3R3/CCR7/RB1/NOD2 |
| GO:0046885 | **regulation of hormone biosynthetic process** | 3/102 | 27/18670 | 0.00042097 | 0.002781596 | 0.001548136 | HIF1A/NFKB1/DKK3 |
| GO:0006304 | **DNA modification** | 5/102 | 116/18670 | 0.000437731 | 0.002861303 | 0.001592498 | WT1/EZH2/UNG/KDM1B/MYC |
| GO:0008361 | **regulation of cell size** | 6/102 | 179/18670 | 0.000449297 | 0.002905718 | 0.001617218 | MTOR/APOE/EDN1/GSK3B/VEGFA/RAB21 |
| GO:0048771 | **tissue remodeling** | 6/102 | 179/18670 | 0.000449297 | 0.002905718 | 0.001617218 | JAG1/CAV1/HIF1A/LIF/ACVR2B/SFRP1 |
| GO:0007613 | **memory** | 5/102 | 117/18670 | 0.000455311 | 0.00293941 | 0.001635969 | MTOR/APOE/ITGA3/LDLR/PTGS2 |
| GO:0001516 | **prostaglandin biosynthetic process** | 3/102 | 28/18670 | 0.000469621 | 0.002989537 | 0.001663868 | EDN1/PTGS2/SIRT1 |
| GO:0071168 | **protein localization to chromatin** | 3/102 | 28/18670 | 0.000469621 | 0.002989537 | 0.001663868 | EZH2/SETD2/RB1 |
| GO:0007164 | **establishment of tissue polarity** | 5/102 | 124/18670 | 0.000593708 | 0.003597696 | 0.002002347 | CLTC/PSMD9/FZD4/FZD6/SFRP1 |
| GO:0030326 | **embryonic limb morphogenesis** | 5/102 | 125/18670 | 0.000615796 | 0.003719202 | 0.002069973 | TFAP2A/SMAD4/TGFB2/FZD6/DLX5 |
| GO:0033687 | **osteoblast proliferation** | 3/102 | 31/18670 | 0.000636753 | 0.003789407 | 0.002109047 | JUNB/SMAD3/SFRP1 |
| GO:0044319 | **wound healing, spreading of cells** | 3/102 | 31/18670 | 0.000636753 | 0.003789407 | 0.002109047 | MTOR/DDR1/CD44 |
| GO:0098742 | **cell-cell adhesion via plasma-membrane adhesion molecules** | 7/102 | 273/18670 | 0.000749368 | 0.004312113 | 0.002399966 | CDH1/CDH2/NECTIN1/TGFB2/WNK1/ALCAM/PTPRT |
| GO:0045995 | **regulation of embryonic development** | 5/102 | 132/18670 | 0.000788115 | 0.004492626 | 0.002500433 | SNAI1/WNT2/WNK1/SFRP1/HNF4A |
| GO:0055007 | **cardiac muscle cell differentiation** | 5/102 | 132/18670 | 0.000788115 | 0.004492626 | 0.002500433 | MTOR/WT1/EDN1/SMAD4/VEGFA |
| GO:0022412 | **cellular process involved in reproduction in multicellular organism** | 8/102 | 361/18670 | 0.000817492 | 0.004624016 | 0.00257356 | MTOR/WT1/SMARCA2/TGFBR1/FZD4/KDM1B/PRDM1/SFRP1 |
| GO:0048332 | **mesoderm morphogenesis** | 4/102 | 77/18670 | 0.000836988 | 0.004709012 | 0.002620866 | ITGA3/SMAD3/SNAI1/SETD2 |
| GO:0000271 | **polysaccharide biosynthetic process** | 4/102 | 78/18670 | 0.000878562 | 0.004878824 | 0.002715377 | MTOR/CLTC/GSK3B/NFKB1 |
| GO:0032801 | **receptor catabolic process** | 3/102 | 35/18670 | 0.000912586 | 0.005037146 | 0.002803493 | APOE/CLTC/BECN1 |
| GO:0097242 | **amyloid-beta clearance** | 3/102 | 36/18670 | 0.000991618 | 0.005391922 | 0.003000949 | APOE/CLTC/LDLR |
| GO:1903364 | **positive regulation of cellular protein catabolic process** | 5/102 | 140/18670 | 0.00102654 | 0.005532417 | 0.003079143 | APOE/CAV1/CTSC/GSK3B/LDLR |
| GO:1905954 | **positive regulation of lipid localization** | 4/102 | 82/18670 | 0.001059481 | 0.005644665 | 0.003141616 | APOE/EDN1/NFKB1/SIRT1 |
| GO:0030212 | **hyaluronan metabolic process** | 3/102 | 37/18670 | 0.001074853 | 0.005700312 | 0.003172587 | CD44/CLTC/NFKB1 |
| GO:0071356 | **cellular response to tumor necrosis factor** | 7/102 | 291/18670 | 0.001087262 | 0.005757759 | 0.00320456 | EDN1/IKBKB/NFKB1/PSMD9/PIAS3/SIRT1/SFRP1 |
| GO:0050708 | **regulation of protein secretion** | 9/102 | 472/18670 | 0.001120467 | 0.005899402 | 0.003283394 | APOE/CCR7/HIF1A/PSMD9/TGFB2/NOD2/XBP1/SFRP1/HNF4A |
| GO:0043154 | **negative regulation of cysteine-type endopeptidase activity involved in apoptotic process** | 4/102 | 84/18670 | 0.001159091 | 0.006007538 | 0.003343578 | CD44/PTGS2/VEGFA/XIAP |
| GO:0006298 | **mismatch repair** | 3/102 | 38/18670 | 0.001162378 | 0.006007538 | 0.003343578 | TP73/SETD2/AXIN2 |
| GO:0045682 | **regulation of epidermis development** | 4/102 | 85/18670 | 0.001211278 | 0.006216306 | 0.003459771 | EZH2/SMAD4/TGFB2/RUNX1 |
| GO:0043114 | **regulation of vascular permeability** | 3/102 | 39/18670 | 0.001254274 | 0.006397235 | 0.00356047 | APOE/VEGFA/CTNNBIP1 |
| GO:0090559 | **regulation of membrane permeability** | 4/102 | 86/18670 | 0.001265089 | 0.006438211 | 0.003583275 | MTOR/TP73/GSK3B/HK2 |
| GO:0033483 | **gas homeostasis** | 2/102 | 10/18670 | 0.001292578 | 0.006478848 | 0.003605892 | CAV1/HIF1A |
| GO:0045348 | **positive regulation of MHC class II biosynthetic process** | 2/102 | 10/18670 | 0.001292578 | 0.006478848 | 0.003605892 | SIRT1/XBP1 |
| GO:0070099 | **regulation of chemokine-mediated signaling pathway** | 2/102 | 10/18670 | 0.001292578 | 0.006478848 | 0.003605892 | EDN1/HIF1A |
| GO:0002690 | **positive regulation of leukocyte chemotaxis** | 4/102 | 87/18670 | 0.001320551 | 0.006556106 | 0.003648891 | CCR7/EDN1/VEGFA/WNK1 |
| GO:0006112 | **energy reserve metabolic process** | 4/102 | 87/18670 | 0.001320551 | 0.006556106 | 0.003648891 | MTOR/GSK3B/KL/MYC |
| GO:0022604 | **regulation of cell morphogenesis** | 9/102 | 484/18670 | 0.001333562 | 0.006611716 | 0.003679842 | APOE/CD44/CDH2/GSK3B/VEGFA/FZD4/RAB21/RND1/ZEB2 |
| GO:0090207 | **regulation of triglyceride metabolic process** | 3/102 | 40/18670 | 0.001350623 | 0.006670976 | 0.003712824 | APOE/LDLR/SLC27A1 |
| GO:0001678 | **cellular glucose homeostasis** | 5/102 | 152/18670 | 0.001479505 | 0.007169907 | 0.003990511 | ERN1/HIF1A/HK2/SIRT1/XBP1 |
| GO:0031058 | **positive regulation of histone modification** | 4/102 | 90/18670 | 0.001497112 | 0.007226482 | 0.004021999 | LIF/SMAD4/VEGFA/SIRT1 |
| GO:0043414 | **macromolecule methylation** | 7/102 | 309/18670 | 0.001534975 | 0.007360629 | 0.00409666 | WT1/EZH2/SMAD4/SIRT1/SETD2/KDM1B/MYC |
| GO:1990845 | **adaptive thermogenesis** | 5/102 | 154/18670 | 0.001567329 | 0.007441275 | 0.004141545 | CAV1/VEGFA/ACVR2B/RB1/IRF4 |
| GO:0002604 | **regulation of dendritic cell antigen processing and presentation** | 2/102 | 11/18670 | 0.001574194 | 0.007441275 | 0.004141545 | CCR7/NOD2 |
| GO:0031652 | **positive regulation of heat generation** | 2/102 | 11/18670 | 0.001574194 | 0.007441275 | 0.004141545 | PTGS2/SLC27A1 |
| GO:0061314 | **Notch signaling involved in heart development** | 2/102 | 11/18670 | 0.001574194 | 0.007441275 | 0.004141545 | JAG1/SNAI1 |
| GO:0044331 | **cell-cell adhesion mediated by cadherin** | 3/102 | 44/18670 | 0.001782107 | 0.008221995 | 0.004576065 | CDH1/CDH2/VEGFA |
| GO:2000241 | **regulation of reproductive process** | 5/102 | 160/18670 | 0.001853864 | 0.008522884 | 0.004743529 | WT1/ACVR1B/LIF/SNAI1/SFRP1 |
| GO:0071216 | **cellular response to biotic stimulus** | 6/102 | 236/18670 | 0.001877948 | 0.008522884 | 0.004743529 | GSK3B/HSPA5/NFKB1/PDE4D/NOD2/XBP1 |
| GO:0010649 | **regulation of cell communication by electrical coupling** | 2/102 | 12/18670 | 0.001882312 | 0.008522884 | 0.004743529 | CAV1/PDE4D |
| GO:0042118 | **endothelial cell activation** | 2/102 | 12/18670 | 0.001882312 | 0.008522884 | 0.004743529 | SMAD4/TGFBR1 |
| GO:0042368 | **vitamin D biosynthetic process** | 2/102 | 12/18670 | 0.001882312 | 0.008522884 | 0.004743529 | NFKB1/SNAI1 |
| GO:0071888 | **macrophage apoptotic process** | 2/102 | 12/18670 | 0.001882312 | 0.008522884 | 0.004743529 | SIRT1/NOD2 |
| GO:0019217 | **regulation of fatty acid metabolic process** | 4/102 | 96/18670 | 0.001898427 | 0.008530978 | 0.004748034 | MTOR/CAV1/PTGS2/SIRT1 |
| GO:1990830 | **cellular response to leukemia inhibitory factor** | 4/102 | 96/18670 | 0.001898427 | 0.008530978 | 0.004748034 | HK2/FZD4/SIRT1/XBP1 |
| GO:0048255 | **mRNA stabilization** | 3/102 | 45/18670 | 0.001901877 | 0.008530978 | 0.004748034 | MTOR/IGF2BP1/AXIN2 |
| GO:0071354 | **cellular response to interleukin-6** | 3/102 | 46/18670 | 0.002026552 | 0.008986674 | 0.005001658 | SMAD4/NFKB1/PTPRT |
| GO:0070301 | **cellular response to hydrogen peroxide** | 4/102 | 99/18670 | 0.002124616 | 0.009342172 | 0.005199515 | ETS1/EZH2/BECN1/SIRT1 |
| GO:0007183 | **SMAD protein complex assembly** | 2/102 | 13/18670 | 0.002216639 | 0.009573993 | 0.005328538 | SMAD3/SMAD4 |
| GO:0010870 | **positive regulation of receptor biosynthetic process** | 2/102 | 13/18670 | 0.002216639 | 0.009573993 | 0.005328538 | EDN1/HIF1A |
| GO:0048661 | **positive regulation of smooth muscle cell proliferation** | 4/102 | 101/18670 | 0.002285347 | 0.009812662 | 0.005461373 | MTOR/EDN1/ERN1/PTGS2 |
| GO:0008542 | **visual learning** | 3/102 | 48/18670 | 0.002290891 | 0.009812662 | 0.005461373 | MTOR/HIF1A/KRAS |
| GO:0051302 | **regulation of cell division** | 5/102 | 168/18670 | 0.002293381 | 0.009812662 | 0.005461373 | TGFB2/VEGFA/BECN1/SETD2/MYC |
